# Role of GPRC6A in Regulating Hepatic Energy Metabolism in Mice

**DOI:** 10.1038/s41598-020-64384-8

**Published:** 2020-04-29

**Authors:** Min Pi, Fuyi Xu, Ruisong Ye, Satoru K. Nishimoto, Robert W. Williams, Lu Lu, L. Darryl Quarles

**Affiliations:** 1 Department of Medicine, , University of Tennessee Health Science Center, 19S Manassas St, Memphis, TN 38163 USA; 2 Department of Genetics, Genomics and Informatics, University of Tennessee Health Science Center, 19S Manassas St, Memphis, TN 38163 USA; 3 Department of Microbiology, Immunology and Biochemistry, University of Tennessee Health Science Center, 19S Manassas St, Memphis, TN 38163 USA

**Keywords:** Molecular biology, Endocrinology

## Abstract

GPRC6A is a widely expressed G-protein coupled receptor that regulates energy metabolism. Global deletion of *Gprc6a* in mice is reported to result in a metabolic syndrome-like phenotype and conditional deletion of *Gprc6a* in pancreatic β-cell and skeletal muscle respectively impair insulin secretion and glucose uptake. In the current study, we explore the hepatic functions of GPRC6A by conditionally deleting *Gprc6a* in hepatocytes by cross breeding *Alb-Cre* and *Gprc6a*^*flox/flox*^ mice to obtain *Gprc6a*^*Liver-cko*^ mice. *Gprc6a*^*Liver-cko*^ mice on a normal diet showed excessive hepatic fat accumulation and glycogen depletion. These mice also exhibit impaired glucose and pyruvate tolerance, but normal insulin sensitivity. Decreased circulating FGF-21 levels and FGF-21 message expression in the liver were found in *Gprc6a*^*Liver-cko*^ mice. Hepatic transcriptome analysis identified alterations in multiple pathways regulating glucose, fat and glycogen metabolism in *Gprc6a*^*Liver-cko*^ mice. Taken together, our studies suggest that GPRC6A directly regulates hepatic metabolism as well as regulates the production and release of FGF-21 to control systemic energy homeostasis. GPRC6A’s unique regulation of β-cell, skeletal muscle and hepatic function may represent a new therapeutic target for treating disordered energy metabolism metabolic syndrome and type 2 diabetes.

## Introduction

GPRC6A is a family C G-protein coupled receptor (GPCR) that consists of an N-terminal venus fly trap motif (VFTM) homologous to the bacterial periplasmic L-amino acid sensor fused with a classical 7 transmembrane domain (7-TM). This structure allows GPRC6A to sense multiple ligands, including the bone-derived peptide, osteocalcin (Ocn), amino acids, L-Arginine, L-Ornithine, and L-Lysine^1–3^, testosterone (T)^1,4^, and natural products in green tea^5^. The bone-derived ligand Ocn regulates energy metabolism by regulating insulin secretion from pancreatic β-cells and testosterone production in Leydig cells^6–9^. *Gprc6a*^*−/−*^ mice exhibit complex metabolic derangements that resemble metabolic syndrome (MetS), including glucose intolerance, insulin resistance, and fatty liver^1,10–14^. The similar phenotypes of *Gprc6a*^*−/−*^ and *Ocn*^*−/−*^ mice and the additive phenotypic effects in compound *Gprc6a*^*+/−*^*/Ocn*^*+/−*^ mice support the presence of this endocrine network in mice^15^.

GPRC6A is expressed in key metabolic tissues, including β-cells, liver hepatocytes, skeletal muscle, fat, and Leydig cells. GPRC6A’s organ-specific functions have been partially examined in mice. Conditional deletion of *Gprc6a* in Leydig cells attenuates Ocn induced testosterone production by the testes^6^, in pancreatic β-cells selective deletion of *Gprc6a* impairs cell proliferation and insulin secretion^11,13^, and selective *Gprc6a* loss-of-function skeletal muscle impairs muscle glucose and fatty acid utilization and IL-6 production^9,14,16^. A potential function of GPRC6A in the liver is suggested by the presence of hepatosteatosis in *Gprc6a*^*−/−*^ mice^10^ and pharmacological actions of Ocn administration to prevent high fat diet induced fatty liver disease in mice^10,17–19^. The direct functions of GPRC6A in the liver, an organ essential for regulating glucose and fat metabolism has not been determined.

In the current study, we advance our understanding of the metabolic functions of GPRC6A by conditionally deleting *Gprc6a* in hepatocytes in male mice. The phenotype in the hepatocyte loss-of-function mouse model establishes a key role of GPRC6A in regulating hepatic glucose and fat metabolism in mice.

## Results

### *In vitro* functions of GPRC6A in muscle and hepatic cells *in vitro*

**Figure 1:**
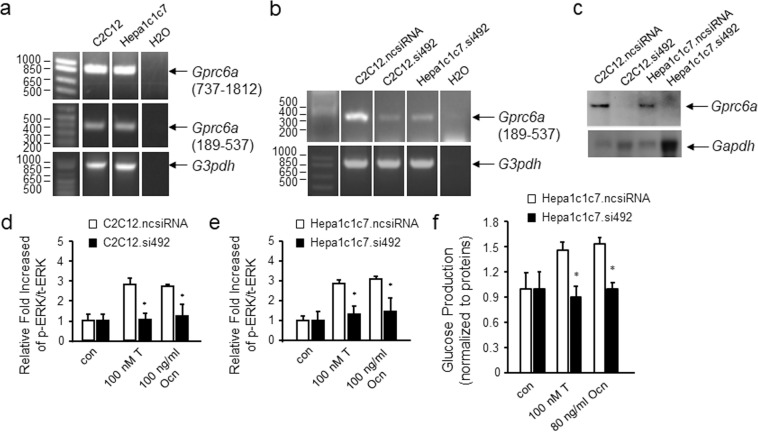
Expression and function of GPRC6A in myocytes and hepatocytes *in vitro*. (**a,b**) Assessment of endogenous GPRC *Gprc6a* 6A message expression by RT-PCR in C2C12 and Hepa1c1c7 cells before (**a**) and after siRNA knockdown of *Gprc6a* (**b**). C2C12.ncsiRNA and Hepa1c1c7.ncsiRNA are C2C12 and Hepa1c1c7 cells transfected with negative control siRNA. (**c**) Western blot of GPRC6A protein in C2C12 and Hepa1c1c7 cells transfected with negative control siRNA or siRNA to GPRC6A. (d and e) ERK activation in response to testosterone (T) and osteocalcin (Ocn) stimulation. siRNA knockdown of *Gprc6a* inhibited these responses in both C2C12 (**d**) and Hepa1c1c7 cells (**e**). Quantification by ERK Elisa assay indicated in Methods section. Values represent the mean ± SEM. *P < 0.05 significant difference between control group and treated group (n = 3). (**f**) Glucose production in mouse Hepa1C1C7 hepatocytes with negative control siRNA or siRNA to knockdown of *Gprc6a*. Values represent the mean ± SEM. *significant difference between Hepa1C1C7 control and siRNA knockdown *Gprc6a* Hepa1C1C7 cells (*P* < 0.05; n = 3).

**Figure 2:**
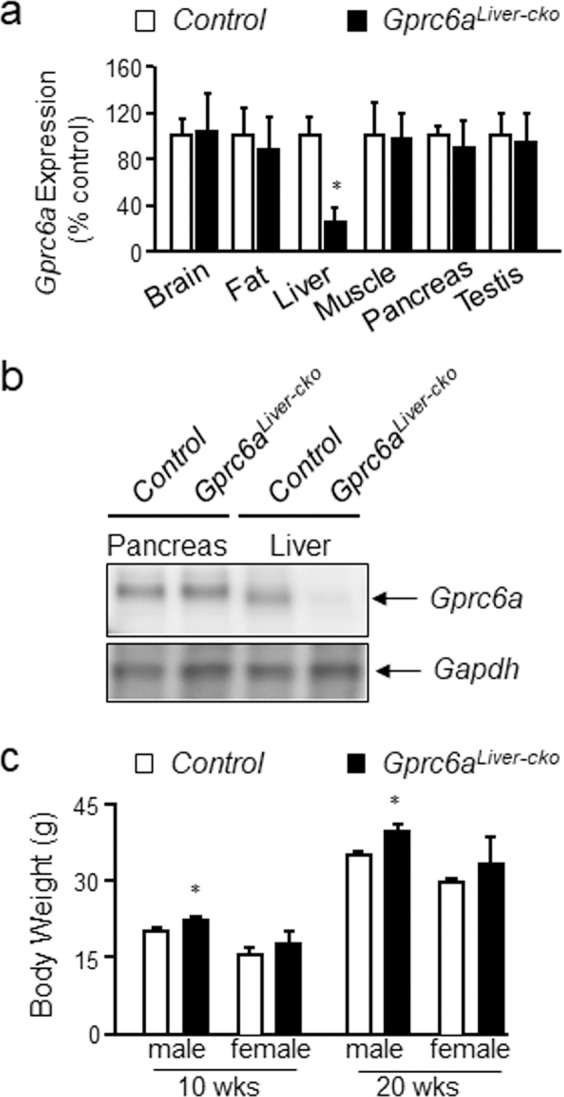
Generation and characterization of *Gprc6a*^*Liver-cko*^ mice. (**a**) Efficiency of *Gprc6a* deletion by *Alb-Cre* in liver was tested by real-time PCR using specific *Gprc6a* primers as described in Materials and Methods. Expression was assessed by real-time PCR using total RNA derived from control wild type mice with unaltered phenotype and *Gprc6a*^*Liver-cko*^ mouse tissues as indicated. *Gprc6a* expression is relative to the level of the *cyclophilin A* control gene. Values represent the mean ± SEM. *significant difference between control group and *Gprc6a*^*Liver-cko*^ mice (*P* < 0.05; n = 5). (**b**) Western blot of GPRC6A protein in liver and pancreas from control and *Gprc6a*^*Liver-cko*^ mice. (**c**) Comparison of the body weight in control and *Gprc6a*^*Liver-cko*^ mice at age of 10 and 20 weeks. Values represent the mean ± SEM. *significant difference between control group and *Gprc6a*^*Liver-cko*^ mice (*P* < 0.05; n = 6).

First, we compared GPRC6A signaling in mouse muscle and hepatic cells *in vitro*. Because conditional knockout of the mouse *Gprc6a* in skeletal muscle has been shown to regulate muscle glucose and fat metabolism^14,16^, we tested the expression, effects of GPRC6A ligands, and knock-down of *Gprc6a* in C2C12 cells. To establish the function of GPRC6A in hepatocytes we performed similar studies the Hepa1c1c7 cell line. Both C2C12 and Hepa1c1c7 express endogenous *Gprc6a* (Fig. 1a). siRNA treatment successfully reduced *Gprc6a* message and protein expression in both cell lines (Fig. 2b,c). T and Ocn, agonists for GPRC6A, also stimulated ERK activity in both cell lines (Fig. 1d,e). siRNA mediated knock down of *Gprc6a* resulted in an attenuation of T and Ocn-induced ERK activation in both muscle and hepatic cell lines, respectively (Fig. 1d,e). T and Ocn also stimulated glucose production in Hepa1c1c7 cells and this response was blocked by siRNA treatment of these cells (Fig. 1f).

### Conditional deletion of *Gprc6a* in hepatocytes in *Gprc6a*^*Liver-cko*^ mice

Next, to test the *in vivo* hepatic function of GPRC6A, we mated *Alb-Cre/+;Gprc6a*^*+/-*^ mice with homozygous *Gprc6a*^*flox/flox*^ mice to generate *Alb- Cre/+;Gprc6a*^*flox/-*^ (*Gprc6a*^*Liver-cko*^), harboring the *liver tissue specific-Cre* transgene. Deletion of *Gprc6a* occurred in the liver in *Gprc6a*^*Liver-cko*^ mice, but not in other tissues tested, including testes, pancreas and muscle (Fig. 2a). Hepatic *Gprc6a* message levels were decreased by>70% in *Gprc6a*^*Liver-cko*^ mice by quantitative RT-PCR (Fig. 2a). Prior studies that targeted *Gprc6a* in muscle found that a 50% reduction of *Gprc6a* was sufficient to achieve a measurable phenotype^14^. Western blot analysis shows Gprc6a protein expression was selectively lost in the liver of *Gprc6a*^*Liver-cko*^ mice (Fig. 2b).

*Gprc6a*^*Liver-cko*^ mice were born with the expected Mendelian frequency and had a normal gross appearance and survival. 10 and 20-week-old male *Gprc6a*^*Liver-cko*^ mice exhibited a significant (p < 0.05) increase in body weight compared to control mice (Fig. 2b), but the body weight of female *Gprc6a*^*Liver-cko*^ mice did not differ from the wild type control group (Fig. 2c).

The wild-type mice (*Gprc6a*^*+/+*^) did not differ from the various intermediate gene construct bearing mice, (i.e.,*Gprc6a*^*flox/*+^*, Gprc6a*^*flox/*-^, *Alb-Cre/*+;*Gprc6a*^*+/*+^and *Alb-Cre/*+;*Gprc6a*^*flox/*+^) with regards to fasting glucose and body weight and were combined to create the control group (Figure S1).

### Serum biochemical alterations in *Gprc6a*^*Liver-cko*^ mice

**Figure 3:**
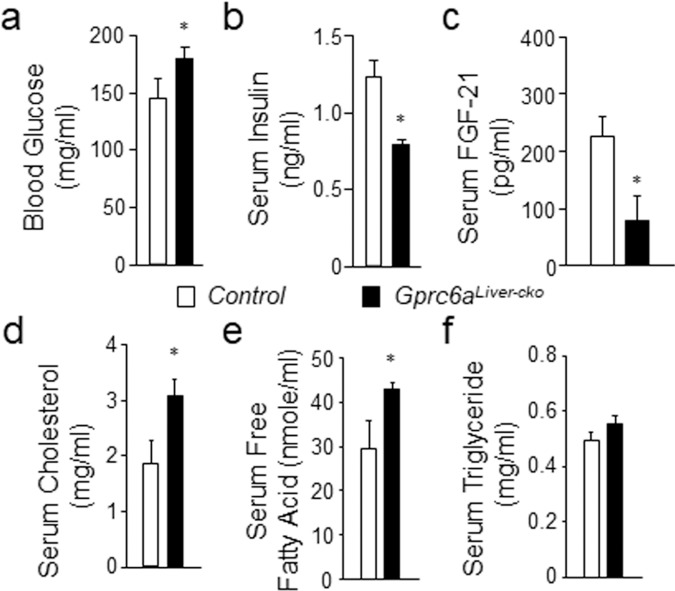
Serum parameters in *Gprc6a*^*Liver-cko*^ mice. Comparison of blood glucose (**a**), serum insulin (**b**) FGF-21 (**c**), cholesterol (**d**), free fatty acid (**e**) and triglyceride levels (**f**) in control group and *Gprc6a*^*Liver-cko*^ male mice at age of 10 weeks. Data represent the mean ± SEM from more than 6 male mice in each group. *difference from control group and *Gprc6a*^*Liver-cko*^ mice at *P* < 0.05.

*Gprc6a*^*Liver-cko*^ male mice had increased fasting blood glucose (Fig. 3a), and decreased serum insulin levels (Fig. 3b) compared to the control mice. We found that the serum level of the liver-derived hepatokine, fibroblast growth factor 21 (FGF-21), was significantly decreased in *Gprc6a*^*Liver-cko*^ mice (80.7 ± 19.9 pg/ml) compared to control mice (225.9 ± 15.4 pg/ml) (Fig. 3c). To investigate effects of hepatic GPRC6A deficiency on lipid metabolism, we measured cholesterol, free fatty acid and triglyceride levels in serum from *Gprc6a*^*Liver-cko*^ mice. The serum cholesterol and free fatty acid levels were increased in *Gprc6a*^*Liver-cko*^ mice (Fig. 3d,e), but serum triglyceride levels were not changed (Fig. 3f).

### Glucose and pyruvate intolerance, but not insulin resistance in *Gprc6a*^*Liver-cko*^ mice

**Figure 4:**
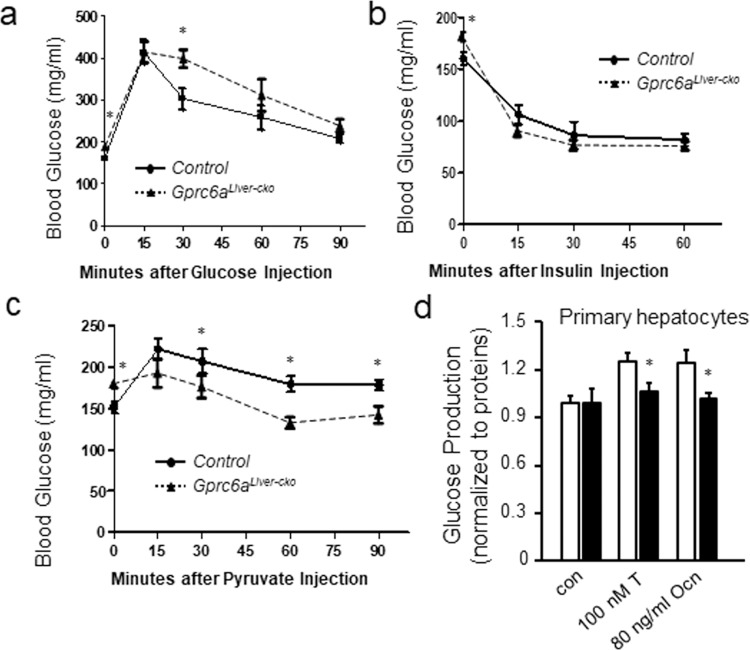
*Gprc6a* deficiency results in glucose and pyruvate intolerance. (**a**–**c**) Shown is blood glucose (mg/dL) during glucose tolerance test (GTT) **(a**), insulin tolerance test (ITT) (**b**) and pyruvate tolerance test (PTT) (**c**) in 10-week-old control and *Gprc6a*^*Liver-cko*^ male mice. Data represent the mean ± SEM from 6 male mice in each group. *difference from control group and *Gprc6a*^*Liver-cko*^ mice at *P* < 0.05. (**d**) Glucose production in hepatocytes isolated from liver of the controls and *Gprc6a*^*Liver-cko*^ mice. Glucose production was measured 12 hours after 100 nM testosterone (T) or 80 ng/ml osteocalcin (Ocn) treatment. Values represent the mean ± SEM. *significant difference between the liver cell of wild type and *Gprc6a*^*Liver-cko*^ mice (*P* < 0.05; n = 6).

To determine the mechanisms for changes in serum glucose, we performed tolerance tests for glucose (GTT) (Fig. 4a) and insulin (ITT) (Fig. 4b) in *Gprc6a*^*Liver-cko*^ and control mice. After injection of glucose (2 g/kg), *Gprc6a*^*Liver-cko*^ male mice had a significantly higher (p < 0.05) serum glucose levels than controls, consistent with impaired glucose tolerance (Fig. 4a). In contrast, *Gprc6a*^*Liver-cko*^ and control mice exhibited a similar sensitivity to insulin (0.75 U/kg) administration (Fig. 4b). In spite of higher fasting glucose, *Gprc6a*^*Liver-cko*^ lowered serum glucose in response to insulin to the same levels as in control mice. This contrast to insulin resistance by ITT in G*prc6a*^*−/−*^ mice^10^, which indicates that loss of *Gprc6a* in multiple sites is required to impart insulin resistance.

To further investigate glucose metabolism in the liver, we performed pyruvate tolerance test (PTT) to investigate GPRC6A’s role in regulating gluconeogenesis (Fig. 4c). PTT was performed a by IP injection of pyruvate sodium (2 g/kg body weight) to *Gprc6a*^*Liver-cko*^ and control mice after fasting for 5 hours. *Gprc6a*^*Liver-cko*^ mice had a significantly lower serum glucose levels during the PTT, consistent with impaired gluconeogenesis (Fig. 4c).

To confirm that GPRC6A regulates hepatic gluconeogenesis *in vitro*, we studied glucose production in the isolated hepatocytes from control and *Gprc6a*^*Liver-cko*^ mice. We found that the GPRC6A ligands Ocn and T increased glucose production in primary hepatocytes from wild type mice (Fig. 4d). This response was significantly attenuated in isolated hepatocytes from *Gprc6a*^*Liver-cko*^ mice. The results, which are like the above studies in Hepa1c1c7 cells expressing endogenous *Gprc6a* before and after siRNA knockdown (Fig. 1), show GPRC6A-dependent increases in glucose production by Ocn and T. Thus, the fasting hyperglycemia in *Gprc6a*^*Liver-cko*^ is not due to enhanced gluconeogenesis or insulin resistance.

### *Gprc6a*^*Liver-cko*^ mice exhibit decreased glycogen content

**Figure 5:**
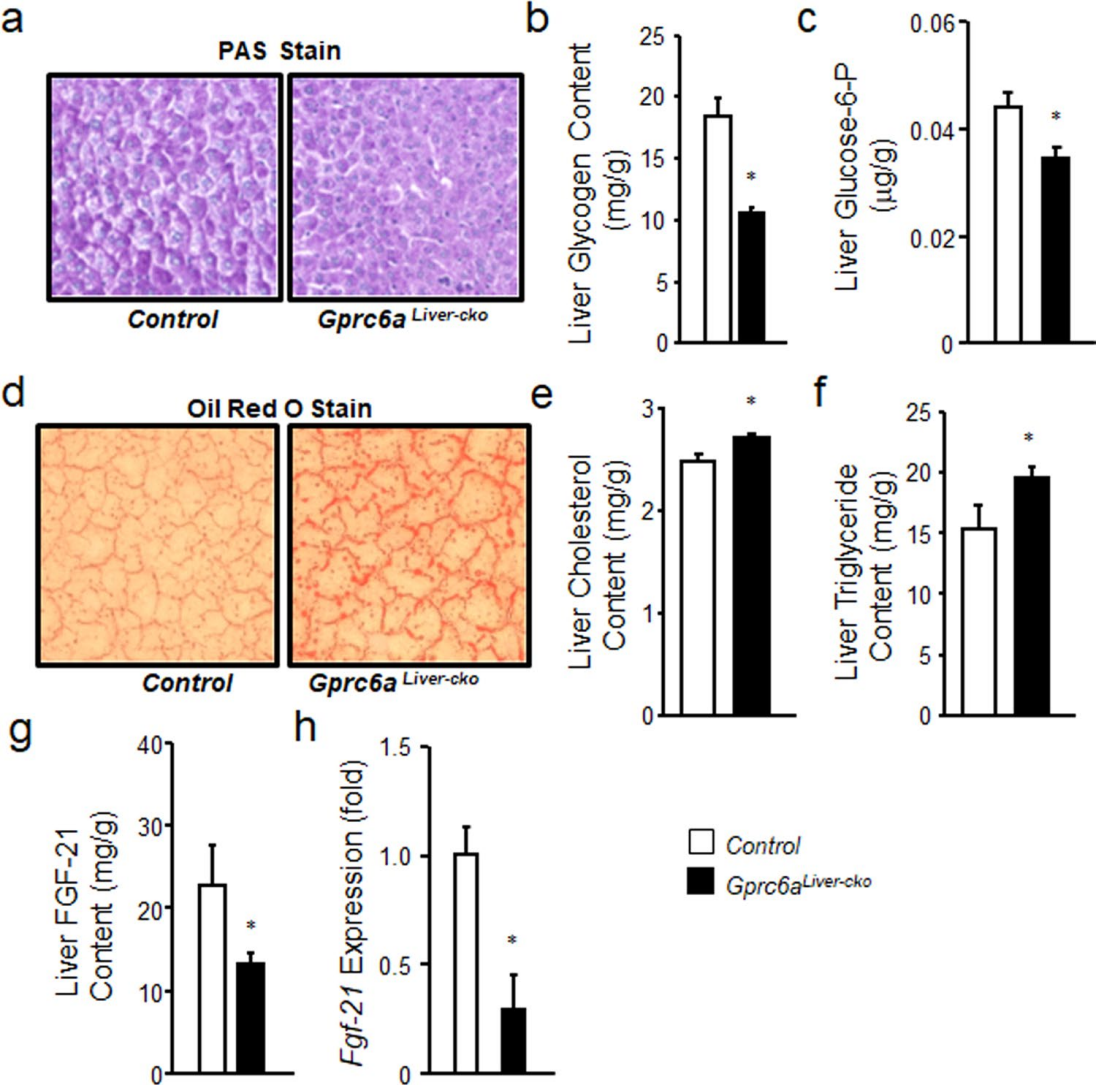
*Gprc6a* deficiency in liver is associated with fatty liver and steatohepatitis. **(a**) PAS staining for glycogen in liver from control group and *Gprc6a*^*Liver-cko*^ male mice. (**b**) Comparison of liver glycogen content in the liver from control group and *Gprc6a*^*Liver-cko*^ male mice. Values represent the mean ± SEM. *significant difference between control group and *Gprc6a*^*Liver-cko*^ mice (*P* < 0.05; n = 5). (**c**) Comparison of the content of glucose-6-phosphate (Glucose-6-P) in liver from control group and *Gprc6a*^*Liver-cko*^ male mice at age of 10 weeks. Values represent the mean ± SEM. *significant difference between control group and *Gprc6a*^*Liver-cko*^ mice (*P* < 0.05; n = 5). (**d**) Representative histology of Oil Red O staining. The result was shown increased hepatic steatosis in *Gprc6a*^*Liver-cko*^ mice. (**e**,**f**) Comparison of the contents of cholesterol (D) and triglyceride (E) in liver from control group and *Gprc6a*^*Liver-cko*^ male mice at age of 10 week-old. Values represent the mean ± SEM. *significant difference between control group and *Gprc6a*^*Liver-cko*^ male mice (*P* < 0.05; n = 5). (**g**,**h**) Comparison of FGF-21 liver content (G) and gene expression (H) in control group and *Gprc6a*^*Liver-cko*^ male mice at age of 10 weeks. Values represent the mean ± SEM. *significant difference between control group and *Gprc6a*^*Liver-cko*^ mice (*P* < 0.05; n = 5).

Consistent with the abnormal pyruvate tolerance test, we found that the liver glycogen storage was significantly decreased in *Gprc6a*^*Liver-cko*^ compared to control group mice as measured by PAS staining of glycogen (Fig. 5a). We also observed reduced liver glycogen content (Fig. 5b) in the liver of *Gprc6a*^*Liver-cko*^ compared to control mice. We found that liver glucose-6-phosphate was significantly reduced in *Gprc6a*^*Liver-cko*^ mice compared to wild type controls (Fig. 5c), consistent with the impaired uptake of glucose and conversion to glycogen.

### *Gprc6a*^*Liver-cko*^ mice develop hepatosteatosis

Consistent with activation of GPRC6A by Ocn to prevent high fat induced fatty liver, the histology in *Gprc6a*^*Liver-cko*^ also showed increase fat accumulation by Oil Red O staining (Fig. 5d). Lipid positive droplets were present in hepatocytes of *Gprc6a*^*Liver-cko*^ mice, but not in control group mice. The liver content of cholesterol and triglyceride were also increased in GPRC6A loss-of-function *Gprc6a*^*Liver-cko*^ mice compared to the control mice (Fig. 5e,f).

### Decreased FGF21 in the liver of Gprc6a^*Liver-cko*^ mice

FGF-21 is primarily produced by the liver. Consistent with the reduced FGF-21 serum levels (Fig. 3c), the liver content of FGF-21 protein (Fig. 5g) was significantly decreased in *Gprc6a*^*Liver-cko*^ mice (12.9 ± 2.1 ng/g) compared to control mice (22.4 ± 3.7 ng/g). We also found that *Fgf-21* message expression level significantly decreased in the liver from *Gprc6a*^*Liver-cko*^ mice compared to control group mice (Fig. 5h). These data indicate for the first time that GPRC6A regulates FGF-21 expression and production by the liver.

### Liver transcriptome in *Gprc6a*^*Liver-cko*^ mice

**Figure 6:**
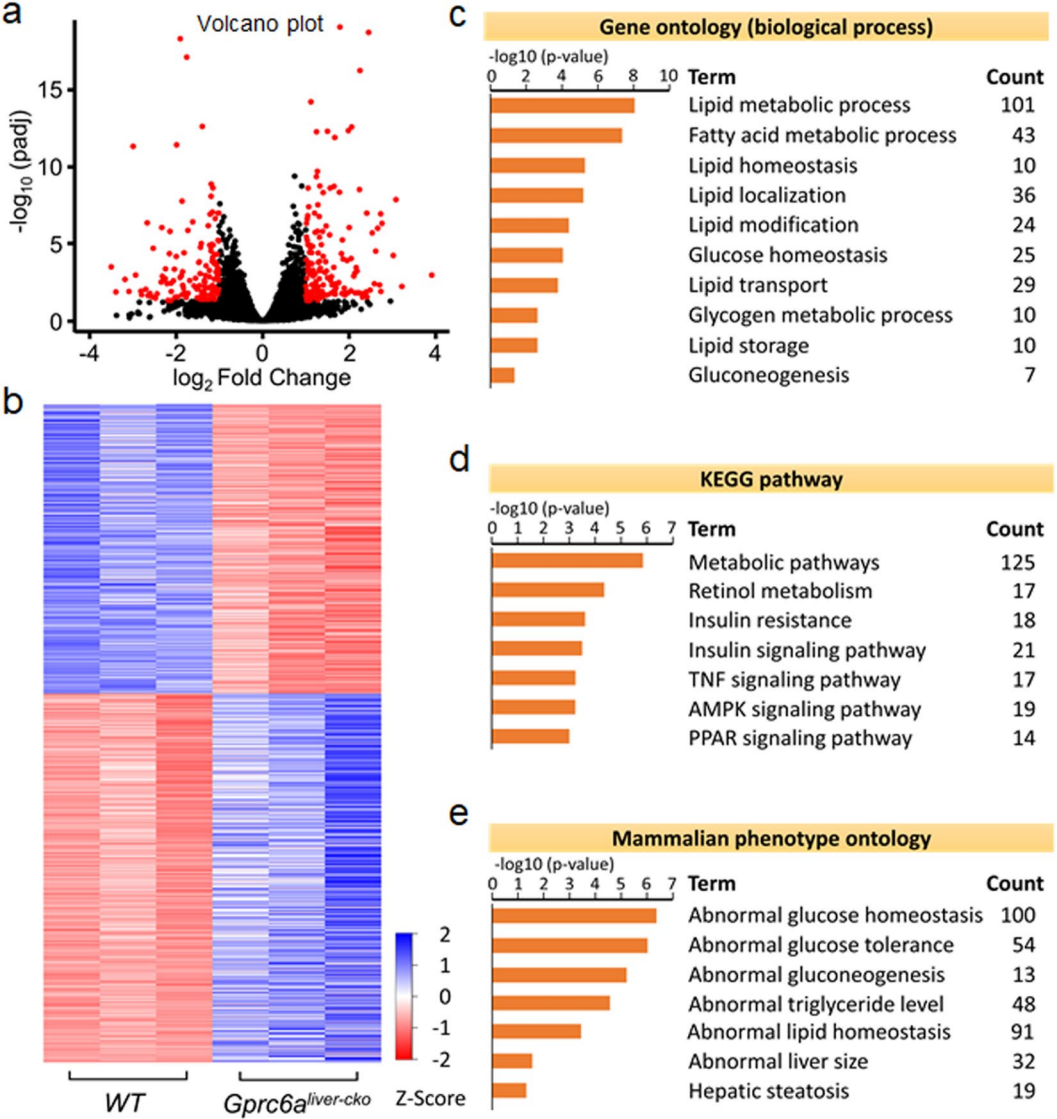
Hepatic gene expression in the liver of *Gprc6a*^*Liver-cko*^ mice. (**a**,**b**) Volcano plot (**a**); Heat map (**b**) of regulated genes between WT and *Gprc6a*^*Liver-cko*^ mice. Blue and red colors indicate high and low gene expression, respectively. Volcano plot and heat map visualization of the hepatic transcriptome demonstrated distinct differences between WT and *Gprc6a*^*Liver-cko*^ mice. (**c**–**e**) Gene ontology (**c**), the Kyoto Encyclopedia of Genes and Genomes pathway (KEGG) (**d**) and Mammalian phenotype (**e)** in the liver of *Gprc6a*^*Liver-cko*^ mice. The top rank ordered processes, maps and networks are based on statistical significance.

To investigate molecular basis for the observed hepatic phenotypes, we assessed the *Gprc6a* hepatic transcriptome by performing RNA-seq analysis on whole liver RNA obtained from *Gprc6a*^*Liver-cko*^ and control mice. Volcano plot and heat map visualization of the hepatic transcriptome demonstrated distinct differences between wild-type and *Gprc6a*^*Liver-cko*^ mouse (Fig. 6a,b). A total of 1208 (677 upregulated and 531 downregulated) genes were identified to be differentially expressed in livers from *Gprc6a*^*Liver-cko*^ mice and controls (adjusted p < 0.05) (Tables S2 and 3). Biological process (GO) enrichment analysis of the ^differentially expressed genes^ (DEGs) revealed that *Gprc6a* specific knockout in liver resulted in differences in lipid and glucose metabolism (Fig. 6c). Genes induced in *Gprc6a*^*Liver-cko*^ mice included genes involved in lipid homeostasis (10 genes), localization (36 genes), modification (24 genes), transport (29 genes), and storage (10 genes).

In addition, the Kyoto Encyclopedia of Genes and Genomes (KEGG) pathway analysis also revealed genes significantly involved in insulin resistance (18 genes), insulin signaling pathway (21 genes), TNF signaling pathway (17 genes), AMPK signaling pathway (19 genes), PPAR signaling pathway (14 genes), and retinol metabolism (17 genes). More importantly, phenotype enrichment analysis of the DEGs revealed that a transcriptional profile pointing to abnormal glucose homeostasis (100 genes) and tolerance (54 genes) (Fig. 6d). Moreover, phenotype enrichment revealed 48 genes are contributed to abnormal triglyceride level such as *Lipc, Abhd5, Lact, Dgat1, Gpihbp1*, and *Angptl4* (Fig. 6e).

**Figure 7:**
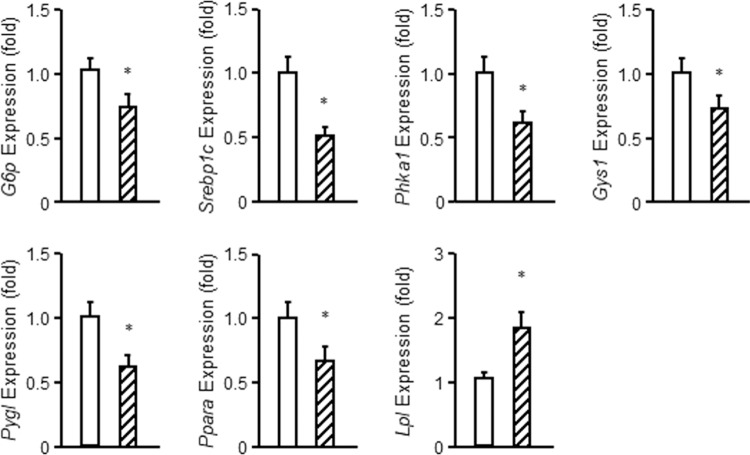
Confirmation of the selected gene expression by real-time PCR. The gene expression was assessed by real-time PCR using total RNA derived from control group mice with unaltered phenotype and *Gprc6a*^*Liver-cko*^ mice. *Gprc6a* expression is relative to the level of the *cyclophilin A* control gene. The real-time PCR using specific primers as described in Table S1. Values represent the mean ± SEM. *significant difference between control group and *Gprc6a*^*Liver-cko*^ mice (*P* < 0.05; n = 5). G6p, glucose 6-phosphatase; Srebp1c, sterol Regulatory Element Binding Transcription Factor 1c; Phka1, phosphorylase kinase alpha 1; Gys1, glycogen synthase 1; Pygl, glycogen Phosphorylase, Liver Form; Ppara, Peroxisome proliferator-activated receptor alpha; Lpl, lipoprotein lipase.

Changes in expression of selected transcripts were confirmed by RT-PCR (Fig. 7). Specific genes altered in *Gprc6a*^*Liver-cko*^ mice included genes involved in glycogen metabolism, such as *Gys1, Slc37a4, Ppp1r3b, Ppp1r3c*, and *Ppp1r3g* and genes involved in gluconeogenesis, such *Pck2, Atf3, Gnmt, Mst1, Per2*, and *Ppargc1a*.

Gene expression analysis revealed that glucose transporter-2 (solute carrier family 2, member A2, *Slc2a2* or *Glut2*), the predominant hepatic bi-directional glucose transporter, was increased in the liver of *Gprc6a*^*Liver-cko*^ mice. Glucokinase (*Gck*), the first rate-limiting step in glucose metabolism, however, was decreased expression in the liver of *Gprc6a*^*Liver-cko*^ consistent with the observed reduced glucose 6-phosphate levels and mild hyperglycemia associated with inactivation of GCK^20^; but genes that regulate glycolysis, including *Gpi, Pdha1, Pdhb, Pklr, Slc2a3, Aldoa, Aldob, Pgam1, Pfkl, Pgm1, Pdhx* and *Creb1*, expression were increased in the liver of *Gprc6a*^*Liver-cko*^.

The expression of gluconeogenesis pathway genes, including *G6p, Pgk1, Pcx, Fbp1, Eno1, Eno3* and *Gck*, were decreased in the liver of *Gprc6a*^*Liver-cko*^ mice, consistent with the impaired pyruvate tolerance test.

With regards to genes regulating glycogen metabolism, we found that the expression of glycogenesis pathway genes, including *Hk1, Pgm2, Gys1*, and glycogen synthase kinase, *Gsk3a* were decreased, consistent with the decreased glycogen stores in the liver of *Gprc6a*^*Liver-cko*^ mice.

Surprisingly, the expression of glycogenolysis pathway genes, including *Pygl*, and branching enzymes, glycogen phosphorylase kinases (*Gaa*), phosphorylase kinase alpha 1 (*Phka1*) and phosphorylase kinase alpha 2 (*Phka2*), were also decreased in the liver of *Gprc6a*^*Liver-cko*^ mice.

Gene transcripts related to fatty acid synthesis, including ATP-citrate lyase (*Acl*), acetylCoA carboxylase^16^, fatty acid synthase enzyme (*Fas*), fatty acid desaturase 2 (*Fads2*), an endoplasmic gene involved in fatty acid metabolism, and long-chain acyl-CoA synthetase (*Acsl*), elongation of long-chain fatty acids family member 6 (*Elovl6*), 3-hydroxyacyl-CoA dehydratase 1 (*Hacd1*), and mitochondrial trans-2-enoyl-CoA reductase (*Mecr*) were increased in the liver from *Gprc6a*^*Liver-cko*^ mice. In contrast, the expression of fatty acid degradation or beta oxidation related genes, acyl-Coenzyme A dehydrogenase, medium chain (*Acadm*), enoyl Coenzyme A hydratase, short chain, 1, mitochondrial (*Echs1*), hydroxyacyl-Coenzyme A dehydrogenase (*Hadh*), and hydroxyacyl-Coenzyme A dehydrogenase, alpha subunit (*Hadha*) were decreased. In addition, the expression of ketogenesis pathway genes, including *Hadha, Hadhb*, 3-hydroxy-3-methylglutaryl-Coenzyme A lyase (*Hmgcl*), 3-hydroxybutyrate dehydrogenase, type 1 (*Bdh1*) and *Bdh2*, were decreased in the liver from *Gprc6a*^*Liver-cko*^ mice. *Srebp-1c* and *Chrebp* transcripts were also increased in *Gprc6a*^*Liver-cko*^, as well as in the global *Gprc6a*^*−/−*^ mice, consistent with increased *de novo* lipogenesis.

## Discussion

**Figure 8:**
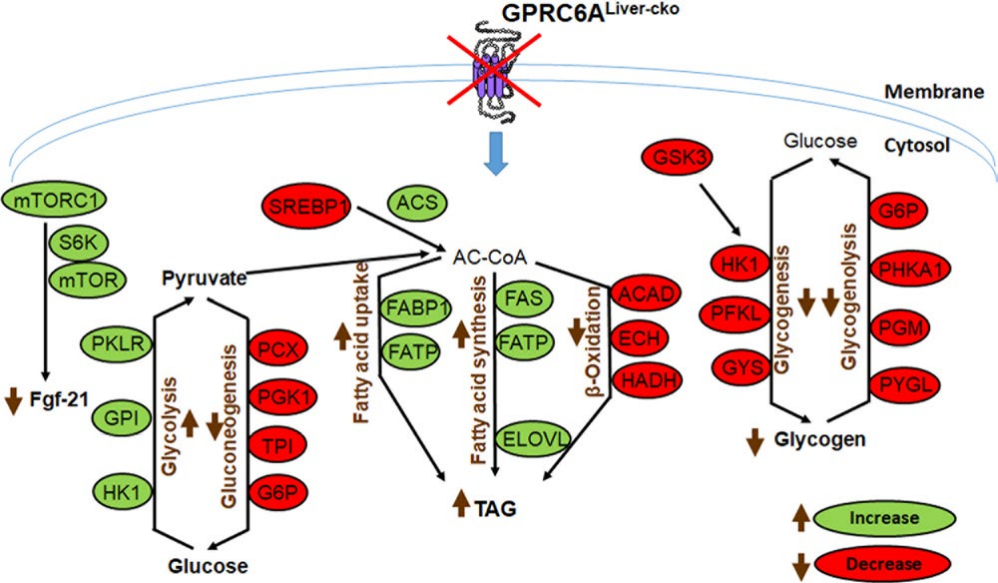
Summary of metabolic pathways in hepatocytes in *Gprc6a*^*Liver-cko*^ mice. Green designates increased gene expression, red indicates decreased gene expression from transcriptome studies in the liver of G*prc6a*^*Liver-cko*^ mice compared to control mice. Glucose is shunted toward triglyceride (TG) and away from glycogen storage.

This study establishes an important role of GPRC6A in the liver to regulate glucose and fat metabolism. Loss-of-*Gprc6a* function in hepatocytes resulted in several metabolic abnormalities in *Gprc6a*^*Liver-cko*^ mice, including hyperglycemia, reduced serum insulin levels, increased circulating concentrations of free fatty acids and cholesterol, impaired glucose and pyruvate tolerance tests, and increased fat and decreased glycogen content in the liver (Fig. 8). These metabolic abnormalities likely resulted from both direct effects of GPRC6A on metabolic pathways regulating glucose and fat metabolism in hepatocytes (Fig. 8), as well as indirect effects mediated by increases in the hepatokine FGF-21.

Indeed, we show for the first time that GPRC6A regulates FGF-21 expression and production in the liver. FGF-21 was reduced 64.3% (p = 0.025) in the circulation and 43.3% in the liver (p = 0.031), a magnitude predicted to contribute to both the hepatic and systemic abnormalities in *Gprc6a*^*Liver-cko*^ mice^21^. FGF-21 regulates glucose and lipid metabolism through systemic activation of fibroblast growth factor receptors complexed to the β-Klotho co-receptor in peripheral and neural tissues, as well as the liver^22–24^. FGF-21 has paracrine effects to regulate hepatic lipid oxidation, triglyceride clearance, ketogenesis, and gluconeogenesis^25^, systemic effects to increase fat browning, glucose and fatty acid utilization and insulin sensitivity in muscle, to increase insulin synthesis^26^ and central nervous system actions to regulated energy intake and sugar consumption^17^. The reduction of FGF-21 in *Gprc6a*^*Liver-cko*^ mice confounds our ability to determine which of the observed alterations in glucose and fat metabolism are due to direct hepatic effects of GPRC6A or indirect systemic effects of these hormonal changes. FGF-21 deficiency, however, might have contributed to the observed weight gain and glucose intolerance in *Gprc6a*^*Liver-cko*^ mice^27^. It will be instructive in future studies to see which of the liver loss-of-function phenotypes will be rescued if FGF-21 is supplemented.

In any event, our findings add FGF-21 to a growing list of hormones regulated by GPRC6A, which include insulin secretion in pancreatic β-cells^3,11^, testosterone (T) production in Leydig cells^6,28^, IL-6 secretion in skeletal muscle^14,16^, lipocalin 2 from adipocytes^29^ and glucagon-like peptide 1 (GLP-1) production from intestinal cells^30–32^. GPRC6A appears to be unique in the complement of the metabolically active hormones that it regulates.

GPRC6A also likely plays a direct role in regulating hepatic glucose and fat metabolism, similar and complementary to the role that GPRC6A plays in regulating catabolism of glucose and fatty acids in skeletal muscle^14,16^. Insights into possible direct hepatic effects of GPRC6A are derived from changes in the expression of key hepatic gene transcripts that control gluconeogenesis, glycolysis, glycogenesis, and glycogenolysis in *Gprc6a*^*Liver-cko*^ mice (Fig. 8). Of the genes that regulate glucose conversion to glycogen, loss-of-GPRC6A decreases glycogenesis as well as glycogenolysis, with the net effects to reduce hepatic glycogen stores in in *Gprc6a*^*Liver-cko*^ mice. In contrast, glycolysis is predicted to be increased leading to conversion of glucose to pyruvate, while gluconeogenesis is decreased in *Gprc6a*^*Liver-cko*^ mice. Down-regulation of the expression of gluconeogenesis pathway genes predicts that *de novo* glucose synthesis form non-carbohydrate precursors is impaired in *Gprc6a*^*Liver-cko*^ mice. A hepatic explanation for the elevation in fasting serum glucose in *Gprc6a*^*Liver-cko*^ mice is not evident from the hepatic transcriptome analysis. Finally, the observed hepatosteatosis and increased hepatic triglyceride content in the liver of *Gprc6a*^*Liver-cko*^ mice may result from multiple changes, including the enhanced conversion of glucose into triacylglycerol in *Gprc6a*-deficient hepatocytes from the increase in glycolysis and decrease in glycogen content in *Gprc6a*^*Liver-cko*^ mice, as well as the increase in fatty acid uptake and synthesis and decrease in β-oxidation. Regardless, the direct hepatic effects of GPRC6A deficiency and reductions in paracrine effects of FGF-21 in the liver are concordant with regards to fatty acid metabolism and triglyceride accumulation.

Additional studies are needed to investigate the signaling and metabolic pathways regulated by GPRC6A in hepatocytes. Like insulin, GPRC6A activates PI3K/AKT/mTOR; but also activates cAMP, like glucagon and GLP-1, as well as ERK^1,2,12^. At the cellular level, the ability of GPRC6A to activate both PI3K/AKT and cAMP pathways creates potentially offsetting signaling mechanisms controlling nutrient uptake and utilization^33,34^.

The metabolic phenotype in *Gprc6a*^*Liver-cko*^ mice is opposite to the reported effects of recombinant Ocn activation of GPRC6A to prevent high fat diet-induced hepatosteatosis, and to improve glucose tolerance, increase insulin sensitivity, reduce fat, and increase muscle mass, ostensibly through GPRC6A activation in multiple tissues^1–4,6,7,12,13,18,35–37^. Testosterone, another ligand for GPRC6A, has non-genomic effects to regulate fat metabolism in the liver^19,38–40^. These observations suggest that activation of GPRC6A may provide a method to treat metabolic syndrome (MetS), type 2 diabetes (T2D), and non-alcoholic fatty liver disease (NAFLD). To this end, novel small molecule agonists for GPRC6A have recently been developed that lower glucose in mice^41^ that may serve as therapeutic leads to develop GPRC6A agonists.

Though some laboratories questioned the function of the mouse GPRC6A, discrepancies in the phenotype of *Gprc6a* deficient mice are likely due to genetic, sex-dependent and environmental factors^3,15,42–47^. Our findings in the liver are consistent with the function of the ancestral ICL3_RKLP mouse variant that now has been confirmed by multiple groups both *in vitro* and *in vivo*^3,15,42,43^. Many studies now show that GPRC6A^RKLP^ is localized to the cell surface and undergoes β-arrestin dependent internalization^44,46,47^. These observations, along with results from *Gprc6a*^*−/−*^ mice, and conditional deletion of *Gprc6a* in β-cells, Leydig cells, and skeletal muscle, indicate that the ancestral *Gprc6a* in mice creates an integrative network for regulating energy metabolism involving direct actions to regulate organ-specific glucose and fat metabolism and indirect effects from the release of multiple hormones from different tissues.

In conclusion, the emerging function of GPRC6A in the liver and other tissues, along with its activation by amino acids, cations, Ocn, T and certain natural products defines a new systems biology in energy metabolism. These data in the liver, along with complementary finding in mice with the conditional deletion of *Gprc6a* in β-cells and skeletal muscle, indicate that GPRC6A plays a key role in glucose and fatty acid metabolism, through both direct tissue effects and the regulation of metabolically active hormones. The afferent and efferent limbs of these endocrine networks created by GPRC6A and its ligands differ from the classic hormonal and metabolic response model to feeding and fasting that are mediated by insulin and glucagon. Rather, variation in GPRC6A activity in response to nutrient, hormone and environmental factors directly controls the anabolic and catabolic functions of multiple organs as well as stimulates the release of an ensemble of hormones that further coordinate glucose and fat metabolism in these various organs.

## Materials and Methods

### Animals

We used global *Gprc6a*^−/−^ and *Gprc6a*^*flox/flox*^ mice that we have previously characterized^10,11^. We used *Alb-Cre* mice^48^ obtained from the Jackson Laboratory [B6.Cg-Tg (Alb-Cre) 21Mgn/J; Bar Harbor, ME, USA] to delete *Gprc6a* in hepatocytes by crossing with *Gprc6a*^*flox/flox*^ mice. We used a strategy that pairs a heterozygous floxed allele with a “null” mutant allele (*Gprc6a*^*+/-*^ mice) to reduce the risk of mosaicism caused by the less than 100% efficiency of Cre-recombinase to excise two floxed alleles (*flox/flox*) and achieves a >75% deletion of *Gprc6a*^11,49,50^. For genotyping, we isolated total DNA from mouse tails, and performed PCR using the primer sets listed in Table S1. Wild-type mice, +*/*+*;Gprc6a*^*flox/*+^, +*/*+*;Gprc6a*^*flox/*-^, *Alb-Cre/*+*;Gprc6a*^*+/*+^ and *Alb-Cre/*+*;Gprc6a*^*flox/*+^ were metabolically indistinguishable and were combined to create the control group.

All mouse strains were maintained in pure C57BL/6 J background more than 10 generations. Mice were maintained and used in accordance with recommendations as described (National Research Council. 1985; Guide for the Care and Use of Laboratory Animals DHHS Publication NIH 86-23, Institute on Laboratory Animal Resources, Rockville, MD, USA) and following guidelines established by the University of Tennessee Health Science Center Institutional Animal Care and Use Committee. The animal study protocol was approved by the institutional review boards at University of Tennessee Health Science Center Institutional Animal Care and Use Committee.

### Metabolic Studies

The glucose tolerance test (GTT) was performed by injecting glucose (2 g/kg body weight) intraperitoneally^51^ after a 5 hours fast, and monitoring blood glucose using glucose strips and the Accu-Check glucometer at the indicated times^52^. For the insulin tolerance test (ITT), mice were fasted for 5 hrs, injected IP with insulin (0.75 U/kg body weight, Sigma; St. Louis, MO, USA), and blood glucose levels were measured at indicated times as described^15^. For the pyruvate tolerance test (PTT), samples were collected following IP injection with pyruvate sodium (2 g/kg bodyweight) to 5 hrs fasted mice. Insulin (mouse) ultrasensitive ELISA kit was obtained from ALPCO Immunoassays (Salem, NH, USA). Glycogen assay and cholesterol quantitation kits were purchased from Sigma (St. Louis, MO, USA). Triglyceride colorimetric assay kit was obtained from Cayman chemical (Ann Arbor, MI, USA). Free fatty acid assay kit was purchased from Fisher Scientific (Pittsburgh, PA, USA). Rat/Mouse FGF-21 Elisa kit purchased from EMD Millipore (Burlington, MA, USA).

### Hepatocyte glucose production

Primary mouse hepatocytes or Hepa1C1C7 cells on 12-well plates (1 × 10^5^ cells/well) were maintained in DMEM medium supplemented with 10% fetal bovine serum (Atlanta Biologicals; Norcross, GA, USA) and 10 units/ml penicillin and 100 μg/ml streptomycin (Invitrogen, Rockville, MA, USA) and 100 nM dexamethasone (Sigma; St. Louis, MO, USA) for 16 hours prior to the measurement of glucose production. Hepatocytes were washed once with PBS, and glucose production was determined after a 12-hour incubation period in glucose-free DMEM containing lactate/pyruvate (10:1 mM) alone or with 100 ng/ml osteocalcin or 100 nM testosterone. At the end of the incubation period, 50 μl of condition medium was collected and glucose level was measured by Glucose assay kit (Sigma; St. Louis, MO, USA).

### Measurement of Total and Phospho‐ERK by ERK Elisa Analysis

Briefly, HEK‐293 cells transfected with/without mouse GPRC6A cDNA plasmid were starved by overnight incubation in serum‐free DMEM/F12 containing 0.1% bovine serum albumin (BSA) and stimulated with various ligands at different doses. ERK activation were assessed 20 min after treatment by using ERK1/2 (phospho‐T203/Y204) ELISA Kit (Invitrogen) corrected for the amount of total ERK using ERK1/2 (Total) ELISA Kit (Invitrogen) to measure ERK levels.

### Histology

Mouse liver tissues were embedded into Tissue-Tek OCT compound from Sakura Finetek USA, Inc. (Torrance, CA, USA). Cryostat sections (6 μm) were air-dried, fixed in 10% buffered formalin for 5 min, and washed in phosphate-buffered saline (PBS) for 10 min. For Oil Red O staining, the cryostat sections were rinsed in H_2_O twice, and wash with 2 ml 60% isopropanol for 5 min at room temperature. After slides were completely dry, 1 ml Oil Red O working solution (0.36% Oil Red O in 60% isopropanol) was added to slide for 15 min at room temperature. Then the slides were rinsed with 60% isopropanol and H_2_O, and dehydrated and mounted. For Periodic Acid-Schiff (PAS) staining, after rinsing in H_2_O, the slide was immersed in 1% Periodic acid solution for 5 min and rinsed in H_2_O. The slide was immersed in Schiff’s reagent (1% pararosaniline HCl, and 4% sodium metabisulfite in 0.25 mol/l hydrochloric acid) for 15 min at room temperature. The slide was counterstained in Hematoxylin solution and mounted.

### RNA extraction

Total RNA was extracted from mouse liver tissue (~30 mg for each sample) using QIAGEN RNeasy Mini Kit (Frederick, MD, USA). QIAGEN RNase-free DNase Set (Frederick, MD, USA) were used for RNA cleanup. RNA quantity was determined by Qubit fluorometer and RNA integrity were determined by Agilent 2100 bioanalyzer (Santa Clara, CA, USA). The qualified samples (RNA integrity number, RIN > 9) were subjected to RNA-seq analysis.

### Real-time RT-PCR

We used 2.0 μg of total RNAs for reverse transcription using cDNA synthesis kit (Bio-Rad). PCR reactions were described in previously publications^10,11^. The primers for mouse *Gprc6a* (NM_153071) consisted of mGPRC6A.For135: catgattggtggcttgtttg and mGprc6a/Rev353: gctgctgtgacttcggtaca, and for the *Cyclophilin A* (NM_008907) consisted of CycA.For: ctgcactgccaagactgaat and CycA. Rev: ccacaatgttcatgccttct.

### siRNA Suppression of *Gprc6a* Gene Expression

For *Gprc6a* knockdown experiments, the short interfering RNAs (siRNA) (CTCAAGG-ATGCTGAACTTA) has been designed from the mGPRC6A sequence (NM_153071). The siRNA hairpins were synthesized and cloned into a pSilencer 4.1-CMV neo vector (Ambion). A circular pSilencer 4.1-CMV neo vector that expresses a hairpin siRNA with limited homology to any known sequence was used as a negative control. The construct of siRNA duplexes has been stably transfected into C2C12 and Hepa1c1c7 cells using Lipofectamine (Invitrogen) and were selected by G418 (Invitrogen). Successful knock down of GPRC6A will be confirmed by assessing RT-PCR analysis of *Gprc6a* RNA and Western blot of GPRC6A protein expression.

### Library preparation and sequencing

The library preparation and sequencing were carried out by Novogene Co., Ltd. (Chula Vista, CA, USA). Briefly, mRNA was first enriched using oligo^53^ beads and fragmented randomly by adding fragmentation buffer. Then the cDNA was synthesized by using mRNA template and random hexamers primer, after which a custom second-strand synthesis buffer (Illumina; Mountain View, CA, USA), dNTPs, RNase H, and DNA polymerase I were added to initiate the second-strand synthesis. Second, after terminal repair, a ligation and sequencing adaptor ligation, the double-stranded cDNA library was completed through size selection and PCR enrichment. The library quality was accessed by Qubit 2.0, Agilent 2100, and Q-PCR. The DNA from the qualified libraries are fed into Illumina sequencers at an average depth of 42 million reads per sample.

### RNA-seq data analysis

Raw reads were quality filtered with NGS QC Toolkit version 2.3^54^ to remove adaptor contaminated reads or reads containing >20% low-quality (*Q* < 20) bases. Filtered reads were aligned to the mouse reference sequence (GRCm38/mm10) using STAR aligner version 2.5.0a^55^. Raw read count was quantified across all annotated mm10 transcript using FeatureCounts version 1.6.3 implemented in Subread package^56^, then submitted to DeSeq. 2 version^57^ to identify the differentially expressed genes between KO and WT groups (three replicates for each group). Differentially expressed genes were defined as having an adjusted p value <0.05. The final significant differential gene list was used for gene enrichment analysis^58^ including Gene Ontology (Biological Process), KEGG pathway^59^, and Mammalian Phenotype Ontology. The heatmap and barplot were prepared with R and Excel program respectively.

### Statistics

We evaluated differences between groups by Studet’s *t* test, and for multiple groups by two-way ANOVA, followed by *a post-hoc* Tukey’s test. Significance was set at p < 0.05. All values are expressed as means ± SEM. All computations were performed using the Statgraphic statistical graphics system (STSC Inc., Rockville, MD, USA).

## Supplementary information

Supplementary Information.

Supplementary Information.
