# Supplementary material for: Role of GPRC6A in Regulating Hepatic Energy Metabolism in Mice
Source: Sci Rep. 2020 Apr 29;10:7216. doi: 10.1038/s41598-020-64384-8 (PMC7190669; doi:10.1038/s41598-020-64384-8)
Supplement: Supplementary file 1 — Supplementary Information. [file 41598_2020_64384_MOESM1_ESM.docx]

**Supplemental information**

**Role of GPRC6A in Regulating Hepatic Energy Metabolism in Mice**

Min Pi^1 *^, Fuyi Xu^2^, Ruisong Ye^1^, Satoru K. Nishimoto^3^, Robert W. Williams^2^, Lu Lu^2^, L. Darryl Quarles^1 *^

^1^Department of Medicine, ^2^Department of Genetics, Genomics and Informatics**,** and ^3^Department of Microbiology, Immunology and Biochemistry,

University of Tennessee Health Science Center, 19 S Manassas St. Memphis, TN 38163

**
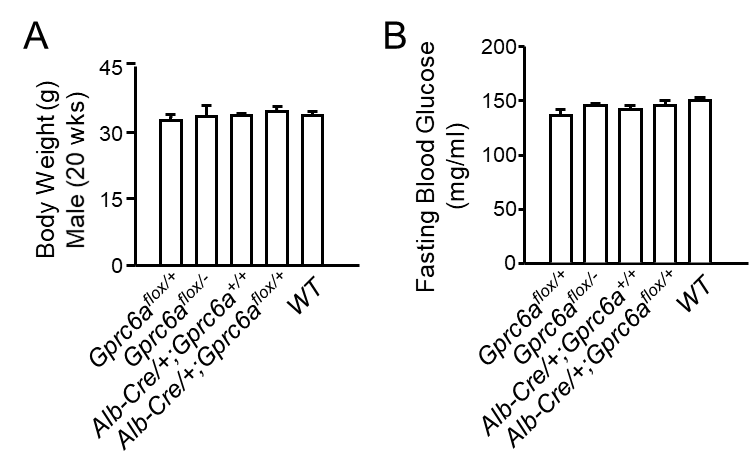
**

**Figure S1.** Comparison of the body weight (A) and blood glucose level (B) in control group mice [+*/*+*;Gprc6a^flox/^*^+^, +*/*+*;Gprc6a^flox/^*^-^, *Alb-Cre/*+*;Gprc6a^+/^*^+^, *Alb-Cre/*+*;Gprc6a^flox/^*^+^ and Wild-type(WT)].

**Table S1. Mouse genotyping primer sets.**

| **Genotype** | **Forward primer** | **Reverse primer** |  |
| --- | --- | --- | --- |
| *Gprc6a^-/-^* | gaataactagcaggaggggcgctggaaggag | cagagtggcagccattgctgctgtgacttcg | Wild type |
|  | cacgagagatcgtggggtatcgacagag | ctacatggcgtgatttcatatgcgcgattgctg | Knockout |
| *Gprc6a^flox/flox^* | acaaagaaaaagaggctatcagctag | tagcagaatgtcttctatttgcttgat | Transgene |
| *Alb-Cre* | tgcaaacatcacatgcacac | ttggccccttaccataactg | Wild type |
|  | gaagcagaagcttaggaagatgg | ttggccccttaccataactg | Transgene |
| **Real-time PCR** |  |  |  |
| *G6p* | cgactcgctatctccaagtga | gggcgttgtccaaacagaat |  |
| *Gys1* | gaacgcagtgcttttcgagg | gctccgtgtatggtcccac |  |
| *Fgf-21* | ctgctgggggtctaccaag | ctgcgcctaccactgttcc |  |
| *Lpl* | tccaaggaagcctttgagaa | ccatcctcagtcccagaaaa |  |
| *Phka1* | cctgggtccgagataatgtgt | cggtcagcatttttgcgatatg |  |
| *Ppara* | agagccccatctgtcctctc | actggtagtctgcaaaaccaaa |  |
| *Pygl* | gagaagcgacggcagatcag | cttgaccagagtgaagtgcag |  |
| *Srepb1c* | tgttggcatcctgctatctg | agggaaagctttggggtcta |  |

**Table S2. Up-regulated gene list in *Gprc6a^Liver-cko^* mice.**

The complete list of up-regulated genes in livers from *Gprc6a^Liver-cko^* mice compared to controls.

| **Ensembl Gene ID** | **Gene name** | **Wild type (WT)** | | | **Gprc6a-liver-cko** | | | **Gprc6a-liver-cko-vs-WT-log2FoldChange** | **Gprc6a-liver-cko-vs-WT-pvalue** | **Gprc6a-liver-cko-vs-WT-padj** |
| --- | --- | --- | --- | --- | --- | --- | --- | --- | --- | --- |
|  |  | **replicate**  **1** | **Replicate**  **2** | **replicate 3** | **Replicate**  **1** | **Replicate**  **2** | **Replicate**  **3** |  |  |  |
| ENSMUSG00000070645 | Ren1 | 0.00 | 0.00 | 0.00 | 48.45 | 55.04 | 63.36 | 8.17 | 8.80E-10 | 2.27E-07 |
| ENSMUSG00000059040 | Eno1b | 50.83 | 29.70 | 55.12 | 2817.44 | 2389.73 | 2960.79 | 5.90 | 1.80E-172 | 2.59E-168 |
| ENSMUSG00000004552 | Ctse | 18.00 | 9.14 | 13.30 | 1027.07 | 798.06 | 537.70 | 5.86 | 2.20E-64 | 1.05E-60 |
| ENSMUSG00000025784 | Clec3b | 0.00 | 6.85 | 1.90 | 15.07 | 49.71 | 63.36 | 3.91 | 2.24E-05 | 1.08E-03 |
| ENSMUSG00000090175 | Ugt1a9 | 698.85 | 616.89 | 310.74 | 9183.34 | 5830.52 | 7651.99 | 3.80 | 2.03E-54 | 7.28E-51 |
| ENSMUSG00000035930 | Chst4 | 0.00 | 6.85 | 1.90 | 16.15 | 17.75 | 45.38 | 3.22 | 2.04E-04 | 5.90E-03 |
| ENSMUSG00000032010 | Usp2 | 196.95 | 305.02 | 242.32 | 1499.69 | 1281.86 | 3516.48 | 3.08 | 3.61E-11 | 1.36E-08 |
| ENSMUSG00000031016 | Wee1 | 131.30 | 83.39 | 63.67 | 430.64 | 343.55 | 1480.40 | 3.02 | 5.77E-07 | 5.71E-05 |
| ENSMUSG00000050097 | Ces2b | 85.77 | 18.28 | 25.66 | 211.01 | 221.93 | 444.38 | 2.76 | 2.06E-09 | 4.76E-07 |
| ENSMUSG00000105703 | Gm43305 | 225.54 | 299.31 | 91.23 | 1308.06 | 1087.45 | 1697.02 | 2.73 | 5.54E-04 | 1.24E-02 |
| ENSMUSG00000052415 | Tchh | 38.12 | 52.55 | 38.01 | 256.23 | 467.83 | 128.43 | 2.73 | 3.88E-10 | 1.14E-07 |
| ENSMUSG00000022346 | Myc | 99.53 | 107.39 | 140.64 | 592.13 | 1503.79 | 166.11 | 2.70 | 1.48E-04 | 4.70E-03 |
| ENSMUSG00000030137 | Tuba8 | 19.06 | 11.42 | 19.01 | 65.67 | 132.27 | 115.59 | 2.66 | 4.97E-09 | 9.90E-07 |
| ENSMUSG00000040152 | Thbs1 | 29.65 | 12.57 | 26.61 | 221.78 | 151.80 | 49.66 | 2.61 | 2.68E-07 | 3.05E-05 |
| ENSMUSG00000025270 | Alas2 | 1449.59 | 1175.53 | 1447.29 | 9199.49 | 7937.07 | 7618.60 | 2.60 | 2.20E-84 | 1.58E-80 |
| ENSMUSG00000103560 | Gm38070 | 8.47 | 15.99 | 13.30 | 54.91 | 22.19 | 148.98 | 2.59 | 1.16E-04 | 3.98E-03 |
| ENSMUSG00000021250 | Fos | 12.71 | 36.56 | 32.31 | 161.49 | 231.69 | 78.77 | 2.53 | 1.17E-08 | 1.99E-06 |
| ENSMUSG00000038415 | Foxq1 | 475.43 | 607.75 | 492.25 | 3694.87 | 4642.75 | 472.63 | 2.48 | 4.27E-04 | 1.03E-02 |
| ENSMUSG00000050069 | Grem2 | 173.65 | 267.32 | 146.34 | 1123.96 | 726.15 | 1346.83 | 2.45 | 1.33E-22 | 1.91E-19 |
| ENSMUSG00000021453 | Gadd45g | 666.03 | 495.80 | 654.75 | 2153.19 | 6294.79 | 1206.41 | 2.41 | 1.78E-03 | 2.77E-02 |
| ENSMUSG00000038550 | Ciart | 46.59 | 54.84 | 128.29 | 372.50 | 212.16 | 638.74 | 2.41 | 3.25E-10 | 1.02E-07 |
| ENSMUSG00000026358 | Rgs1 | 6.35 | 13.71 | 7.60 | 15.07 | 60.36 | 68.50 | 2.39 | 6.35E-04 | 1.36E-02 |
| ENSMUSG00000022388 | Ttll8 | 6.35 | 22.85 | 5.70 | 72.13 | 18.64 | 80.48 | 2.31 | 9.23E-04 | 1.75E-02 |
| ENSMUSG00000027171 | Prrg4 | 2.12 | 6.85 | 7.60 | 19.38 | 27.52 | 35.10 | 2.31 | 1.94E-03 | 2.92E-02 |
| ENSMUSG00000105987 | AI506816 | 73.06 | 55.98 | 72.22 | 273.45 | 373.73 | 311.66 | 2.25 | 5.67E-20 | 5.81E-17 |
| ENSMUSG00000056487 | Mettl7a2 | 31.77 | 27.42 | 33.26 | 158.26 | 136.71 | 142.99 | 2.24 | 7.15E-12 | 3.02E-09 |
| ENSMUSG00000091366 | Gm17040 | 8.47 | 25.13 | 7.60 | 38.76 | 23.08 | 129.29 | 2.23 | 7.38E-04 | 1.52E-02 |
| ENSMUSG00000044949 | Ubtd2 | 6.35 | 11.42 | 6.65 | 17.23 | 24.86 | 67.64 | 2.18 | 7.53E-04 | 1.54E-02 |
| ENSMUSG00000035783 | Acta2 | 29.65 | 28.56 | 81.72 | 284.22 | 285.84 | 68.50 | 2.18 | 1.00E-05 | 5.86E-04 |
| ENSMUSG00000030208 | Emp1 | 14.82 | 61.69 | 30.41 | 88.28 | 287.62 | 104.46 | 2.17 | 1.01E-05 | 5.86E-04 |
| ENSMUSG00000028680 | Plk3 | 1060.98 | 1613.06 | 1063.37 | 8379.13 | 6000.96 | 2333.19 | 2.16 | 5.36E-06 | 3.53E-04 |
| ENSMUSG00000050423 | Ppp1r3g | 91.06 | 52.55 | 28.51 | 213.17 | 101.20 | 443.52 | 2.14 | 7.70E-04 | 1.56E-02 |
| ENSMUSG00000090264 | Eif4ebp3 | 20.12 | 41.13 | 21.86 | 120.58 | 65.69 | 175.52 | 2.13 | 1.59E-05 | 8.32E-04 |
| ENSMUSG00000090165 | Ugt1a10 | 15.88 | 17.14 | 12.35 | 81.82 | 55.93 | 57.37 | 2.11 | 2.68E-05 | 1.26E-03 |
| ENSMUSG00000026773 | Pfkfb3 | 592.96 | 783.68 | 781.14 | 4128.74 | 2195.32 | 2670.53 | 2.06 | 3.09E-16 | 2.60E-13 |
| ENSMUSG00000054675 | Tmem119 | 21.18 | 43.41 | 19.01 | 109.81 | 108.30 | 125.01 | 2.05 | 9.81E-07 | 9.02E-05 |
| ENSMUSG00000028957 | Per3 | 290.13 | 263.89 | 248.03 | 1002.31 | 559.26 | 1738.97 | 2.04 | 4.92E-04 | 1.14E-02 |
| ENSMUSG00000020593 | Lpin1 | 4818.90 | 3508.30 | 2211.32 | 17496.80 | 10625.07 | 14070.18 | 2.00 | 4.36E-09 | 8.81E-07 |
| ENSMUSG00000042622 | Maff | 191.65 | 173.64 | 219.52 | 1090.59 | 721.71 | 500.03 | 1.98 | 5.73E-16 | 4.57E-13 |
| ENSMUSG00000035385 | Ccl2 | 29.65 | 18.28 | 38.01 | 142.11 | 168.67 | 27.40 | 1.97 | 7.51E-04 | 1.54E-02 |
| ENSMUSG00000002068 | Ccne1 | 48.71 | 31.99 | 54.17 | 105.51 | 161.56 | 255.15 | 1.95 | 1.44E-06 | 1.23E-04 |
| ENSMUSG00000040434 | Large2 | 9.53 | 9.14 | 5.70 | 25.84 | 27.52 | 38.53 | 1.93 | 8.42E-04 | 1.65E-02 |
| ENSMUSG00000043110 | Lrrn4 | 12.71 | 31.99 | 15.20 | 17.23 | 72.79 | 131.86 | 1.90 | 1.24E-03 | 2.16E-02 |
| ENSMUSG00000020205 | Phlda1 | 692.50 | 1074.99 | 691.81 | 3002.62 | 5237.52 | 898.17 | 1.89 | 1.11E-03 | 2.00E-02 |
| ENSMUSG00000053113 | Socs3 | 274.25 | 640.88 | 404.82 | 1159.49 | 2785.65 | 932.42 | 1.89 | 2.89E-04 | 7.60E-03 |
| ENSMUSG00000059824 | Dbp | 1430.53 | 1916.94 | 1618.34 | 5846.98 | 3642.30 | 8539.89 | 1.86 | 1.10E-06 | 9.86E-05 |
| ENSMUSG00000075511 | 1700001L05Rik | 76.24 | 66.26 | 76.02 | 180.87 | 197.07 | 405.85 | 1.84 | 3.82E-08 | 5.71E-06 |
| ENSMUSG00000097251 | 5033417F24Rik | 38.12 | 9.14 | 13.30 | 88.28 | 46.16 | 81.34 | 1.83 | 8.68E-04 | 1.68E-02 |
| ENSMUSG00000030554 | Synm | 42.35 | 47.98 | 57.02 | 152.88 | 133.16 | 235.46 | 1.82 | 4.15E-07 | 4.35E-05 |
| ENSMUSG00000021573 | Tppp | 393.90 | 468.38 | 361.11 | 1206.86 | 1256.12 | 1751.82 | 1.79 | 5.38E-23 | 8.58E-20 |
| ENSMUSG00000039633 | Lonrf1 | 364.25 | 335.86 | 356.36 | 1151.95 | 671.11 | 1789.49 | 1.77 | 1.11E-11 | 4.53E-09 |
| ENSMUSG00000056071 | S100a9 | 19.06 | 34.27 | 24.71 | 88.28 | 118.95 | 56.51 | 1.76 | 9.30E-05 | 3.29E-03 |
| ENSMUSG00000028212 | Ccne2 | 25.41 | 26.28 | 17.11 | 40.91 | 85.22 | 95.90 | 1.70 | 2.53E-04 | 6.94E-03 |
| ENSMUSG00000045934 | Mtmr11 | 16.94 | 26.28 | 6.65 | 45.22 | 72.79 | 39.39 | 1.68 | 1.05E-03 | 1.92E-02 |
| ENSMUSG00000019737 | Syne4 | 21.18 | 75.40 | 33.26 | 155.03 | 130.49 | 126.72 | 1.67 | 1.18E-04 | 4.01E-03 |
| ENSMUSG00000091971 | Hspa1a | 82.59 | 33.13 | 23.76 | 118.43 | 231.69 | 93.33 | 1.67 | 4.51E-04 | 1.07E-02 |
| ENSMUSG00000025981 | Coq10b | 579.20 | 736.85 | 664.25 | 1885.12 | 1531.31 | 2877.74 | 1.67 | 1.86E-15 | 1.27E-12 |
| ENSMUSG00000066153 | Mup21 | 2637.63 | 1530.81 | 1929.09 | 8607.36 | 3938.80 | 6638.24 | 1.65 | 3.91E-12 | 1.87E-09 |
| ENSMUSG00000009108 | Gnat2 | 27.53 | 51.41 | 39.91 | 167.95 | 113.63 | 89.90 | 1.64 | 5.00E-06 | 3.37E-04 |
| ENSMUSG00000104475 | #N/A | 367.43 | 571.20 | 260.38 | 685.79 | 1022.65 | 2029.23 | 1.64 | 5.51E-04 | 1.24E-02 |
| ENSMUSG00000019997 | Ctgf | 87.89 | 70.83 | 114.99 | 209.94 | 529.97 | 93.33 | 1.60 | 1.51E-04 | 4.75E-03 |
| ENSMUSG00000026981 | Il1rn | 38.12 | 43.41 | 38.01 | 77.51 | 173.99 | 107.88 | 1.59 | 2.97E-05 | 1.35E-03 |
| ENSMUSG00000026388 | 3110009E18Rik | 16.94 | 15.99 | 32.31 | 67.83 | 88.77 | 40.24 | 1.58 | 8.35E-04 | 1.64E-02 |
| ENSMUSG00000029552 | Tes | 30.71 | 94.82 | 52.27 | 169.03 | 237.91 | 119.01 | 1.57 | 5.84E-05 | 2.32E-03 |
| ENSMUSG00000032311 | Nrg4 | 176.83 | 129.09 | 133.04 | 369.27 | 402.14 | 522.29 | 1.56 | 5.52E-12 | 2.40E-09 |
| ENSMUSG00000027035 | Cers6 | 199.07 | 245.62 | 240.42 | 488.77 | 466.94 | 1048.01 | 1.55 | 1.62E-08 | 2.67E-06 |
| ENSMUSG00000026628 | Atf3 | 31.77 | 34.27 | 43.71 | 109.81 | 134.93 | 66.78 | 1.50 | 3.63E-05 | 1.60E-03 |
| ENSMUSG00000030228 | Pik3c2g | 268.95 | 239.90 | 192.91 | 617.96 | 736.80 | 627.61 | 1.50 | 6.69E-16 | 5.05E-13 |
| ENSMUSG00000019947 | Arid5b | 669.20 | 1908.94 | 788.74 | 3805.76 | 3516.24 | 2135.40 | 1.49 | 1.18E-03 | 2.09E-02 |
| ENSMUSG00000044548 | Dact1 | 74.12 | 118.81 | 95.98 | 152.88 | 276.08 | 372.45 | 1.48 | 1.01E-05 | 5.86E-04 |
| ENSMUSG00000021365 | Nedd9 | 148.24 | 175.93 | 171.05 | 515.69 | 486.47 | 369.03 | 1.47 | 3.27E-09 | 7.01E-07 |
| ENSMUSG00000055320 | Tead1 | 266.83 | 313.02 | 217.62 | 478.01 | 450.96 | 1260.35 | 1.46 | 1.79E-06 | 1.45E-04 |
| ENSMUSG00000029380 | Cxcl1 | 160.95 | 268.46 | 224.27 | 418.79 | 720.83 | 631.89 | 1.44 | 1.34E-05 | 7.24E-04 |
| ENSMUSG00000031734 | Irx3 | 29.65 | 57.12 | 36.11 | 159.34 | 134.05 | 37.67 | 1.43 | 1.35E-03 | 2.29E-02 |
| ENSMUSG00000030256 | Bhlhe41 | 103.77 | 246.76 | 144.44 | 398.34 | 411.01 | 494.89 | 1.40 | 6.63E-07 | 6.52E-05 |
| ENSMUSG00000039470 | Zdhhc2 | 50.83 | 25.13 | 45.61 | 107.66 | 104.75 | 109.60 | 1.40 | 1.50E-04 | 4.73E-03 |
| ENSMUSG00000061906 | Ugt2b38 | 831.21 | 801.96 | 631.94 | 1754.85 | 2273.44 | 1945.32 | 1.40 | 1.20E-11 | 4.77E-09 |
| ENSMUSG00000094786 | Gm14403 | 350.48 | 595.19 | 453.29 | 970.01 | 1136.28 | 1556.60 | 1.39 | 8.66E-09 | 1.57E-06 |
| ENSMUSG00000098708 | Gm27252 | 44.47 | 26.28 | 45.61 | 128.11 | 74.57 | 102.75 | 1.39 | 1.68E-03 | 2.68E-02 |
| ENSMUSG00000028943 | Espn | 24.35 | 19.42 | 30.41 | 49.52 | 71.02 | 72.78 | 1.38 | 7.24E-04 | 1.50E-02 |
| ENSMUSG00000027699 | Ect2 | 69.89 | 36.56 | 41.81 | 85.05 | 90.55 | 208.92 | 1.38 | 5.71E-04 | 1.25E-02 |
| ENSMUSG00000055866 | Per2 | 676.62 | 512.94 | 492.25 | 1251.00 | 898.37 | 2191.91 | 1.37 | 1.12E-03 | 2.01E-02 |
| ENSMUSG00000037465 | Klf10 | 1086.40 | 1103.55 | 522.66 | 2940.18 | 2087.02 | 1923.92 | 1.36 | 8.38E-08 | 1.13E-05 |
| ENSMUSG00000023046 | Igfbp6 | 46.59 | 143.94 | 81.72 | 90.43 | 266.31 | 333.92 | 1.35 | 1.45E-03 | 2.42E-02 |
| ENSMUSG00000020268 | Lyrm7 | 58.24 | 36.56 | 60.82 | 134.57 | 130.49 | 129.29 | 1.34 | 5.97E-06 | 3.86E-04 |
| ENSMUSG00000084839 | Gm14097 | 67.77 | 137.09 | 73.17 | 164.72 | 123.39 | 406.70 | 1.33 | 1.09E-03 | 1.98E-02 |
| ENSMUSG00000022139 | Mbnl2 | 1086.40 | 1385.73 | 982.60 | 2251.16 | 2648.94 | 3749.37 | 1.32 | 3.57E-12 | 1.79E-09 |
| ENSMUSG00000024222 | Fkbp5 | 1471.82 | 2496.14 | 1384.57 | 3300.84 | 2970.30 | 7130.56 | 1.32 | 2.51E-03 | 3.48E-02 |
| ENSMUSG00000030659 | Nucb2 | 124.95 | 141.66 | 104.53 | 391.88 | 362.19 | 174.67 | 1.32 | 9.64E-06 | 5.74E-04 |
| ENSMUSG00000052302 | Tbc1d30 | 346.25 | 566.63 | 296.49 | 972.16 | 894.82 | 1139.62 | 1.32 | 2.37E-08 | 3.74E-06 |
| ENSMUSG00000022206 | Npr3 | 57.18 | 59.40 | 68.42 | 135.65 | 147.36 | 177.24 | 1.31 | 1.67E-06 | 1.38E-04 |
| ENSMUSG00000037447 | Arid5a | 57.18 | 93.68 | 121.64 | 219.63 | 298.27 | 159.26 | 1.31 | 4.44E-05 | 1.86E-03 |
| ENSMUSG00000045441 | Gprin3 | 240.36 | 365.57 | 155.85 | 487.70 | 461.61 | 934.99 | 1.31 | 8.73E-06 | 5.33E-04 |
| ENSMUSG00000025780 | Itih5 | 496.61 | 688.86 | 446.64 | 1297.30 | 1695.54 | 1040.30 | 1.31 | 6.34E-10 | 1.72E-07 |
| ENSMUSG00000032085 | Tagln | 316.60 | 397.55 | 334.50 | 1063.67 | 1084.79 | 416.98 | 1.29 | 2.04E-06 | 1.61E-04 |
| ENSMUSG00000032577 | Mapkapk3 | 109.06 | 180.50 | 160.60 | 400.49 | 264.54 | 432.39 | 1.29 | 3.68E-07 | 3.97E-05 |
| ENSMUSG00000031433 | Rbm41 | 59.30 | 60.55 | 38.01 | 100.12 | 103.86 | 178.09 | 1.28 | 3.59E-04 | 9.01E-03 |
| ENSMUSG00000083287 | Gm13502 | 27.53 | 39.98 | 32.31 | 101.20 | 102.09 | 39.39 | 1.28 | 3.77E-03 | 4.61E-02 |
| ENSMUSG00000048279 | Sacs | 85.77 | 57.12 | 64.62 | 96.89 | 102.98 | 303.10 | 1.28 | 1.56E-03 | 2.54E-02 |
| ENSMUSG00000057425 | Ugt2b37 | 90.00 | 84.54 | 89.33 | 119.50 | 189.97 | 328.79 | 1.28 | 2.09E-04 | 6.00E-03 |
| ENSMUSG00000023963 | Cyp39a1 | 417.19 | 550.63 | 364.91 | 856.97 | 1304.05 | 1061.71 | 1.28 | 8.42E-11 | 2.95E-08 |
| ENSMUSG00000041220 | Elovl6 | 7528.54 | 5203.61 | 5792.02 | 11802.69 | 8212.26 | 24831.95 | 1.28 | 2.89E-03 | 3.84E-02 |
| ENSMUSG00000092021 | Gbp11 | 55.06 | 62.83 | 71.27 | 175.48 | 144.70 | 137.85 | 1.27 | 3.54E-04 | 8.88E-03 |
| ENSMUSG00000026072 | Il1r1 | 631.08 | 1157.25 | 744.08 | 2054.14 | 1767.44 | 2281.81 | 1.27 | 8.34E-09 | 1.55E-06 |
| ENSMUSG00000006154 | Eps8l1 | 36.00 | 31.99 | 50.37 | 113.04 | 112.74 | 60.79 | 1.27 | 5.54E-04 | 1.24E-02 |
| ENSMUSG00000012428 | Steap4 | 4815.72 | 8120.15 | 5846.18 | 15859.30 | 15755.18 | 13542.75 | 1.27 | 3.35E-13 | 2.00E-10 |
| ENSMUSG00000017002 | Slpi | 23.30 | 60.55 | 43.71 | 48.45 | 120.73 | 133.57 | 1.25 | 3.15E-03 | 4.06E-02 |
| ENSMUSG00000026574 | Dpt | 44.47 | 109.67 | 131.14 | 120.58 | 316.03 | 241.45 | 1.25 | 1.69E-03 | 2.68E-02 |
| ENSMUSG00000025612 | Bach1 | 1625.36 | 1942.07 | 1360.82 | 4113.66 | 3382.20 | 4186.89 | 1.25 | 7.62E-16 | 5.47E-13 |
| ENSMUSG00000089774 | Slc5a3 | 237.19 | 210.20 | 244.22 | 536.14 | 506.89 | 595.93 | 1.24 | 8.02E-13 | 4.43E-10 |
| ENSMUSG00000041153 | Osgin2 | 54.00 | 74.26 | 54.17 | 105.51 | 134.93 | 186.65 | 1.23 | 3.78E-04 | 9.40E-03 |
| ENSMUSG00000001506 | Col1a1 | 169.42 | 283.31 | 400.07 | 723.47 | 885.94 | 395.57 | 1.23 | 7.73E-05 | 2.88E-03 |
| ENSMUSG00000067818 | Myl9 | 95.30 | 75.40 | 70.32 | 243.31 | 177.54 | 142.13 | 1.22 | 1.41E-04 | 4.56E-03 |
| ENSMUSG00000034645 | Zyg11a | 96.36 | 196.49 | 143.49 | 376.81 | 159.79 | 480.34 | 1.22 | 2.18E-04 | 6.17E-03 |
| ENSMUSG00000054178 | Gm9938 | 26.47 | 47.98 | 23.76 | 60.29 | 78.12 | 88.19 | 1.22 | 2.00E-03 | 2.99E-02 |
| ENSMUSG00000047368 | Abhd17b | 692.50 | 856.80 | 726.02 | 1420.03 | 1396.38 | 2465.04 | 1.22 | 1.06E-08 | 1.82E-06 |
| ENSMUSG00000028862 | Map3k6 | 72.00 | 63.97 | 58.92 | 157.18 | 168.67 | 125.86 | 1.21 | 1.83E-05 | 9.19E-04 |
| ENSMUSG00000023805 | Synj2 | 164.12 | 148.51 | 163.45 | 330.51 | 383.49 | 380.16 | 1.20 | 3.27E-10 | 1.02E-07 |
| ENSMUSG00000032224 | Fam81a | 42.35 | 86.82 | 58.92 | 145.34 | 176.66 | 108.74 | 1.20 | 3.47E-04 | 8.76E-03 |
| ENSMUSG00000054150 | Syne3 | 200.13 | 313.02 | 192.91 | 370.35 | 325.79 | 911.01 | 1.19 | 1.84E-04 | 5.42E-03 |
| ENSMUSG00000042246 | Tmc7 | 144.01 | 143.94 | 70.32 | 333.74 | 342.66 | 136.99 | 1.18 | 5.68E-04 | 1.25E-02 |
| ENSMUSG00000017667 | Zfp334 | 58.24 | 57.12 | 60.82 | 109.81 | 127.83 | 157.54 | 1.17 | 2.67E-05 | 1.26E-03 |
| ENSMUSG00000025019 | Lcor | 349.43 | 355.29 | 202.41 | 517.84 | 576.13 | 932.42 | 1.16 | 8.51E-06 | 5.24E-04 |
| ENSMUSG00000022389 | Tef | 4538.30 | 5151.06 | 4568.99 | 9215.64 | 7392.90 | 15137.88 | 1.15 | 1.13E-07 | 1.46E-05 |
| ENSMUSG00000032515 | Csrnp1 | 928.63 | 751.70 | 631.94 | 2035.84 | 2120.75 | 952.97 | 1.14 | 2.64E-06 | 1.94E-04 |
| ENSMUSG00000012123 | Crybg2 | 100.59 | 53.69 | 63.67 | 117.35 | 205.95 | 157.54 | 1.14 | 5.50E-04 | 1.24E-02 |
| ENSMUSG00000027864 | Ptgfrn | 101.65 | 185.07 | 120.69 | 195.94 | 271.64 | 428.11 | 1.14 | 1.43E-04 | 4.60E-03 |
| ENSMUSG00000028654 | Mycl | 857.68 | 566.63 | 437.13 | 1727.93 | 1184.21 | 1181.58 | 1.14 | 3.41E-07 | 3.74E-05 |
| ENSMUSG00000021670 | Hmgcr | 3700.74 | 4574.15 | 3035.23 | 12075.07 | 8286.83 | 4411.22 | 1.13 | 1.05E-03 | 1.92E-02 |
| ENSMUSG00000066071 | Cyp4a12a | 14579.52 | 15633.69 | 13858.08 | 31689.53 | 33634.67 | 30318.58 | 1.12 | 6.54E-18 | 6.26E-15 |
| ENSMUSG00000021253 | Tgfb3 | 37.06 | 50.27 | 66.52 | 86.13 | 146.47 | 101.03 | 1.12 | 1.92E-03 | 2.89E-02 |
| ENSMUSG00000027533 | Fabp5 | 1327.82 | 975.61 | 845.76 | 1440.48 | 1938.77 | 3409.45 | 1.11 | 3.40E-03 | 4.26E-02 |
| ENSMUSG00000049047 | Armcx3 | 160.95 | 170.22 | 178.65 | 301.45 | 442.97 | 352.76 | 1.11 | 1.36E-07 | 1.69E-05 |
| ENSMUSG00000024235 | Map3k8 | 36.00 | 70.83 | 57.02 | 127.04 | 121.62 | 102.75 | 1.10 | 6.92E-04 | 1.45E-02 |
| ENSMUSG00000035914 | Cd276 | 113.30 | 156.51 | 150.15 | 192.71 | 371.07 | 332.21 | 1.10 | 1.10E-05 | 6.23E-04 |
| ENSMUSG00000052837 | Junb | 2441.74 | 2301.93 | 2235.08 | 6394.96 | 5475.43 | 3031.00 | 1.09 | 2.43E-06 | 1.84E-04 |
| ENSMUSG00000053553 | 3110082I17Rik | 380.13 | 424.97 | 401.97 | 1072.29 | 645.37 | 860.50 | 1.09 | 9.18E-08 | 1.21E-05 |
| ENSMUSG00000038301 | Snx10 | 839.68 | 1003.02 | 927.48 | 1325.29 | 1819.82 | 2722.76 | 1.08 | 3.24E-07 | 3.60E-05 |
| ENSMUSG00000038174 | Fam126b | 1039.81 | 881.93 | 938.89 | 1458.78 | 1354.65 | 3242.49 | 1.08 | 1.69E-05 | 8.71E-04 |
| ENSMUSG00000024411 | Aqp4 | 78.36 | 66.26 | 51.32 | 86.13 | 92.32 | 234.60 | 1.08 | 4.10E-03 | 4.90E-02 |
| ENSMUSG00000045064 | Zc2hc1c | 240.36 | 222.77 | 148.25 | 480.16 | 482.92 | 317.66 | 1.07 | 8.71E-06 | 5.33E-04 |
| ENSMUSG00000026064 | Ptp4a1 | 2021.37 | 2030.04 | 1947.14 | 3454.79 | 3715.98 | 5395.01 | 1.07 | 1.21E-10 | 4.02E-08 |
| ENSMUSG00000026620 | Mark1 | 63.53 | 86.82 | 45.61 | 114.12 | 181.09 | 113.88 | 1.07 | 4.15E-03 | 4.95E-02 |
| ENSMUSG00000066361 | Serpina3c | 45.53 | 54.84 | 29.46 | 97.97 | 87.00 | 84.77 | 1.06 | 3.82E-03 | 4.66E-02 |
| ENSMUSG00000047037 | Nipa1 | 64.59 | 77.68 | 61.77 | 144.26 | 149.14 | 130.14 | 1.06 | 2.55E-03 | 3.53E-02 |
| ENSMUSG00000070436 | Serpinh1 | 513.55 | 637.46 | 598.68 | 1291.91 | 1537.52 | 797.99 | 1.05 | 3.10E-06 | 2.23E-04 |
| ENSMUSG00000048807 | Slc35e4 | 31.77 | 27.42 | 34.21 | 66.75 | 60.36 | 66.78 | 1.05 | 4.16E-03 | 4.95E-02 |
| ENSMUSG00000029657 | Hsph1 | 3202.01 | 2737.18 | 2904.09 | 5929.88 | 7067.11 | 5309.39 | 1.05 | 5.47E-12 | 2.40E-09 |
| ENSMUSG00000004891 | Nes | 241.42 | 169.07 | 193.86 | 514.61 | 376.39 | 361.32 | 1.05 | 5.12E-06 | 3.40E-04 |
| ENSMUSG00000038119 | Cdon | 52.94 | 57.12 | 55.12 | 123.81 | 80.78 | 136.99 | 1.05 | 2.29E-03 | 3.27E-02 |
| ENSMUSG00000020137 | Thap2 | 223.42 | 238.76 | 236.62 | 427.41 | 424.33 | 589.93 | 1.05 | 8.33E-08 | 1.13E-05 |
| ENSMUSG00000021466 | Ptch1 | 1731.25 | 1517.10 | 1185.01 | 3290.07 | 3246.38 | 2610.60 | 1.05 | 5.41E-10 | 1.49E-07 |
| ENSMUSG00000059810 | Rgs3 | 360.01 | 727.71 | 428.58 | 1115.35 | 1098.10 | 909.30 | 1.04 | 1.07E-05 | 6.08E-04 |
| ENSMUSG00000030729 | Pgm2l1 | 199.07 | 157.65 | 151.10 | 219.63 | 266.31 | 557.40 | 1.04 | 8.76E-04 | 1.69E-02 |
| ENSMUSG00000069833 | Ahnak | 585.55 | 711.71 | 747.88 | 1164.87 | 1475.38 | 1546.32 | 1.03 | 9.48E-10 | 2.39E-07 |
| ENSMUSG00000025269 | Apex2 | 224.48 | 285.60 | 282.24 | 533.99 | 502.45 | 584.79 | 1.03 | 3.97E-08 | 5.87E-06 |
| ENSMUSG00000042549 | Map2k3os | 44.47 | 83.39 | 36.11 | 122.73 | 103.86 | 104.46 | 1.02 | 4.02E-03 | 4.83E-02 |
| ENSMUSG00000026348 | Acmsd | 879.92 | 740.27 | 753.58 | 1901.26 | 2043.52 | 866.49 | 1.02 | 4.10E-05 | 1.77E-03 |
| ENSMUSG00000050248 | Evc2 | 47.65 | 45.70 | 49.42 | 77.51 | 84.33 | 126.72 | 1.02 | 2.72E-03 | 3.69E-02 |
| ENSMUSG00000054321 | Taf4b | 67.77 | 119.95 | 112.13 | 156.11 | 227.26 | 221.76 | 1.02 | 4.69E-04 | 1.10E-02 |
| ENSMUSG00000003134 | Tbc1d8 | 696.73 | 997.31 | 706.07 | 1492.16 | 1342.23 | 2006.11 | 1.01 | 1.25E-06 | 1.10E-04 |
| ENSMUSG00000022299 | Slc25a32 | 586.61 | 587.19 | 487.50 | 896.80 | 1068.81 | 1380.22 | 1.01 | 2.27E-08 | 3.63E-06 |
| ENSMUSG00000029189 | Sel1l3 | 508.26 | 799.68 | 681.36 | 1226.24 | 1384.84 | 1396.49 | 1.01 | 2.12E-09 | 4.76E-07 |
| ENSMUSG00000036362 | P2ry13 | 48.71 | 73.11 | 74.12 | 114.12 | 134.93 | 144.70 | 1.01 | 1.77E-03 | 2.76E-02 |
| ENSMUSG00000021765 | Fst | 152.48 | 99.39 | 102.63 | 228.24 | 344.43 | 136.99 | 1.00 | 1.33E-03 | 2.27E-02 |
| ENSMUSG00000026674 | Ddr2 | 141.89 | 227.34 | 175.80 | 235.77 | 427.88 | 424.68 | 1.00 | 1.93E-04 | 5.62E-03 |
| ENSMUSG00000021775 | Nr1d2 | 2361.27 | 2875.41 | 2258.84 | 4320.37 | 4316.07 | 6343.70 | 1.00 | 6.54E-09 | 1.27E-06 |
| ENSMUSG00000054115 | Skp2 | 48.71 | 50.27 | 49.42 | 61.37 | 106.53 | 126.72 | 0.99 | 3.63E-03 | 4.49E-02 |
| ENSMUSG00000055675 | Kbtbd11 | 40.24 | 29.70 | 72.22 | 92.59 | 110.96 | 81.34 | 0.99 | 3.49E-03 | 4.34E-02 |
| ENSMUSG00000035133 | Arhgap5 | 1505.71 | 1775.28 | 1187.86 | 1992.77 | 2616.99 | 4262.24 | 0.99 | 1.03E-05 | 5.91E-04 |
| ENSMUSG00000097426 | Gm8941 | 263.66 | 467.24 | 233.77 | 755.77 | 335.56 | 821.11 | 0.99 | 1.76E-03 | 2.75E-02 |
| ENSMUSG00000057897 | Camk2b | 476.49 | 278.74 | 347.81 | 564.13 | 750.12 | 869.06 | 0.99 | 5.12E-05 | 2.10E-03 |
| ENSMUSG00000028766 | Alpl | 514.61 | 626.03 | 490.35 | 1368.35 | 1262.33 | 598.49 | 0.99 | 4.16E-05 | 1.79E-03 |
| ENSMUSG00000022816 | Fstl1 | 158.83 | 207.92 | 237.57 | 340.20 | 530.85 | 318.51 | 0.98 | 1.18E-04 | 4.00E-03 |
| ENSMUSG00000030660 | Pik3c2a | 1472.88 | 1528.53 | 1023.46 | 1671.95 | 2238.82 | 3998.52 | 0.98 | 6.00E-05 | 2.36E-03 |
| ENSMUSG00000074622 | Mafb | 3712.38 | 3238.69 | 2118.20 | 6122.59 | 4579.73 | 6989.28 | 0.96 | 8.12E-07 | 7.77E-05 |
| ENSMUSG00000015501 | Hivep2 | 210.71 | 257.04 | 282.24 | 483.39 | 510.44 | 469.21 | 0.96 | 8.24E-06 | 5.12E-04 |
| ENSMUSG00000054942 | Miga1 | 74.12 | 78.83 | 54.17 | 118.43 | 102.98 | 179.81 | 0.96 | 2.03E-03 | 3.02E-02 |
| ENSMUSG00000086265 | Marcksl1-ps4 | 324.01 | 281.03 | 331.65 | 644.88 | 442.08 | 729.50 | 0.95 | 2.50E-06 | 1.87E-04 |
| ENSMUSG00000070720 | Tmem200b | 112.24 | 149.65 | 113.08 | 245.46 | 322.24 | 156.69 | 0.95 | 7.68E-04 | 1.56E-02 |
| ENSMUSG00000020423 | Btg2 | 468.02 | 445.53 | 456.14 | 819.29 | 1342.23 | 486.33 | 0.95 | 2.51E-04 | 6.91E-03 |
| ENSMUSG00000022360 | Atad2 | 139.77 | 98.25 | 95.98 | 195.94 | 237.91 | 209.77 | 0.95 | 1.79E-04 | 5.34E-03 |
| ENSMUSG00000031750 | Il34 | 59.30 | 89.11 | 91.23 | 172.25 | 172.22 | 118.16 | 0.95 | 1.32E-03 | 2.27E-02 |
| ENSMUSG00000004631 | Sgce | 110.12 | 81.11 | 83.63 | 92.59 | 265.43 | 169.53 | 0.94 | 2.63E-03 | 3.61E-02 |
| ENSMUSG00000020099 | Unc5b | 249.89 | 162.22 | 188.16 | 338.05 | 410.12 | 400.71 | 0.94 | 1.69E-05 | 8.72E-04 |
| ENSMUSG00000024298 | Zfp871 | 556.96 | 531.21 | 366.81 | 692.25 | 662.24 | 1428.17 | 0.94 | 4.35E-04 | 1.04E-02 |
| ENSMUSG00000020601 | Trib2 | 82.59 | 59.40 | 72.22 | 148.57 | 152.69 | 107.88 | 0.93 | 1.24E-03 | 2.16E-02 |
| ENSMUSG00000005057 | Sh2b2 | 95.30 | 171.36 | 138.74 | 273.45 | 236.13 | 261.15 | 0.93 | 1.44E-04 | 4.62E-03 |
| ENSMUSG00000035293 | G2e3 | 105.89 | 74.26 | 165.35 | 164.72 | 184.64 | 304.81 | 0.92 | 3.23E-03 | 4.14E-02 |
| ENSMUSG00000060803 | Gstp1 | 77026.14 | 93878.69 | 80397.38 | 200277.59 | 169978.94 | 103041.20 | 0.91 | 2.99E-06 | 2.18E-04 |
| ENSMUSG00000033715 | Akr1c14 | 15632.04 | 14187.42 | 11003.41 | 20080.62 | 24010.94 | 32441.13 | 0.91 | 3.20E-07 | 3.59E-05 |
| ENSMUSG00000053646 | Plxnb1 | 2001.26 | 2185.40 | 2236.98 | 3948.94 | 4263.70 | 3823.86 | 0.91 | 3.62E-12 | 1.79E-09 |
| ENSMUSG00000029591 | Ung | 266.83 | 487.80 | 378.22 | 697.63 | 513.99 | 896.46 | 0.90 | 4.38E-04 | 1.05E-02 |
| ENSMUSG00000035992 | Fnip1 | 1045.10 | 1035.01 | 787.79 | 1356.51 | 1631.62 | 2344.32 | 0.90 | 3.07E-06 | 2.22E-04 |
| ENSMUSG00000026043 | Col3a1 | 628.97 | 1063.57 | 914.18 | 1213.32 | 2073.70 | 1558.31 | 0.89 | 3.90E-05 | 1.71E-03 |
| ENSMUSG00000024378 | Stard4 | 6857.22 | 8168.13 | 6252.91 | 12072.92 | 11100.89 | 16189.31 | 0.89 | 1.21E-08 | 2.05E-06 |
| ENSMUSG00000026107 | Nabp1 | 548.49 | 447.82 | 504.60 | 872.04 | 966.72 | 924.71 | 0.88 | 3.19E-07 | 3.59E-05 |
| ENSMUSG00000039457 | Ppl | 491.31 | 470.67 | 315.50 | 735.31 | 942.75 | 647.30 | 0.87 | 4.16E-05 | 1.79E-03 |
| ENSMUSG00000038370 | Pcp4l1 | 169.42 | 151.94 | 205.26 | 311.14 | 244.12 | 404.99 | 0.87 | 1.76E-04 | 5.31E-03 |
| ENSMUSG00000007613 | Tgfbr1 | 998.51 | 1003.02 | 979.75 | 1382.35 | 1816.27 | 2221.02 | 0.86 | 1.66E-07 | 1.96E-05 |
| ENSMUSG00000091387 | Gcnt4 | 231.89 | 306.16 | 218.57 | 521.07 | 515.76 | 338.20 | 0.86 | 1.06E-04 | 3.65E-03 |
| ENSMUSG00000027397 | Slc20a1 | 623.67 | 606.61 | 834.35 | 1085.21 | 1154.92 | 1514.64 | 0.86 | 1.17E-06 | 1.03E-04 |
| ENSMUSG00000029167 | Ppargc1a | 264.72 | 195.35 | 105.48 | 342.36 | 367.51 | 315.09 | 0.86 | 1.25E-03 | 2.16E-02 |
| ENSMUSG00000026042 | Col5a2 | 76.24 | 89.11 | 115.94 | 163.64 | 205.95 | 140.42 | 0.86 | 1.67E-03 | 2.67E-02 |
| ENSMUSG00000032265 | Tent5a | 330.37 | 269.61 | 271.78 | 342.36 | 410.12 | 820.25 | 0.85 | 1.85E-03 | 2.83E-02 |
| ENSMUSG00000025757 | Hspa4l | 843.92 | 766.55 | 790.64 | 1420.03 | 1752.35 | 1154.18 | 0.85 | 1.02E-05 | 5.91E-04 |
| ENSMUSG00000029333 | #N/A | 317.66 | 345.00 | 435.23 | 599.66 | 1029.75 | 347.62 | 0.85 | 2.94E-03 | 3.89E-02 |
| ENSMUSG00000047719 | Ubiad1 | 658.61 | 660.30 | 565.42 | 1162.72 | 1031.53 | 1193.56 | 0.85 | 2.52E-09 | 5.56E-07 |
| ENSMUSG00000094114 | #N/A | 122.83 | 138.23 | 109.28 | 161.49 | 198.85 | 303.10 | 0.85 | 2.31E-03 | 3.28E-02 |
| ENSMUSG00000062901 | Klhl24 | 2347.51 | 2692.63 | 2003.21 | 3505.39 | 4166.94 | 4954.92 | 0.84 | 3.75E-07 | 4.01E-05 |
| ENSMUSG00000030244 | Gys2 | 5656.46 | 6206.64 | 5509.78 | 8836.68 | 7820.78 | 14434.07 | 0.84 | 7.03E-06 | 4.45E-04 |
| ENSMUSG00000038894 | Irs2 | 728.50 | 1232.65 | 704.16 | 1537.38 | 1377.73 | 1850.28 | 0.84 | 1.29E-04 | 4.28E-03 |
| ENSMUSG00000071637 | Cebpd | 364.25 | 310.73 | 340.20 | 579.21 | 751.01 | 484.62 | 0.84 | 1.12E-05 | 6.33E-04 |
| ENSMUSG00000040648 | Ppip5k2 | 1713.24 | 1810.70 | 1391.22 | 2405.11 | 2996.04 | 3376.06 | 0.84 | 1.50E-07 | 1.80E-05 |
| ENSMUSG00000040891 | Foxa3 | 2991.30 | 3587.12 | 3867.68 | 7211.02 | 5228.65 | 6163.89 | 0.83 | 2.23E-08 | 3.60E-06 |
| ENSMUSG00000027351 | Spred1 | 735.91 | 896.78 | 760.23 | 1336.05 | 1295.18 | 1626.81 | 0.83 | 3.75E-08 | 5.66E-06 |
| ENSMUSG00000034342 | Cbl | 83.65 | 90.25 | 71.27 | 132.42 | 158.90 | 143.84 | 0.83 | 2.35E-03 | 3.31E-02 |
| ENSMUSG00000021061 | Sptb | 74.12 | 91.39 | 145.39 | 165.80 | 192.63 | 195.22 | 0.83 | 3.26E-03 | 4.15E-02 |
| ENSMUSG00000020893 | Per1 | 676.62 | 687.72 | 520.76 | 1198.25 | 775.86 | 1370.80 | 0.83 | 3.47E-05 | 1.55E-03 |
| ENSMUSG00000023073 | Slc10a2 | 687.20 | 556.35 | 681.36 | 933.41 | 1426.56 | 1055.71 | 0.83 | 6.77E-06 | 4.32E-04 |
| ENSMUSG00000027806 | Tsc22d2 | 1459.12 | 1578.79 | 1265.79 | 2889.58 | 2391.51 | 2332.33 | 0.82 | 9.62E-09 | 1.70E-06 |
| ENSMUSG00000044768 | D1Ertd622e | 579.20 | 539.21 | 643.35 | 998.00 | 1025.31 | 1092.53 | 0.82 | 7.04E-09 | 1.34E-06 |
| ENSMUSG00000037111 | Setd7 | 220.24 | 379.28 | 378.22 | 370.35 | 679.99 | 675.55 | 0.82 | 1.05E-03 | 1.92E-02 |
| ENSMUSG00000000303 | Cdh1 | 1334.17 | 1823.26 | 1468.20 | 2791.61 | 3086.59 | 2292.09 | 0.82 | 1.32E-06 | 1.14E-04 |
| ENSMUSG00000034723 | Tmx4 | 1137.22 | 1199.52 | 1026.31 | 1504.00 | 1281.86 | 3145.73 | 0.82 | 1.16E-03 | 2.06E-02 |
| ENSMUSG00000029186 | Pi4k2b | 1554.42 | 1329.75 | 1260.08 | 2174.72 | 2469.63 | 2657.69 | 0.82 | 2.12E-09 | 4.76E-07 |
| ENSMUSG00000031365 | Zfp275 | 281.66 | 342.72 | 240.42 | 454.32 | 438.53 | 626.75 | 0.82 | 7.86E-05 | 2.90E-03 |
| ENSMUSG00000022565 | Plec | 5635.28 | 6837.24 | 5582.95 | 9089.68 | 8335.65 | 14321.91 | 0.81 | 1.24E-05 | 6.85E-04 |
| ENSMUSG00000069114 | Zbtb10 | 218.13 | 299.31 | 165.35 | 426.33 | 444.75 | 326.22 | 0.81 | 3.76E-04 | 9.36E-03 |
| ENSMUSG00000025278 | Flnb | 3906.16 | 4193.74 | 3226.23 | 5828.68 | 4515.81 | 9458.61 | 0.81 | 1.77E-04 | 5.32E-03 |
| ENSMUSG00000040596 | Pogk | 403.43 | 461.53 | 303.14 | 533.99 | 485.58 | 1019.75 | 0.81 | 1.47E-03 | 2.45E-02 |
| ENSMUSG00000026728 | Vim | 782.50 | 909.35 | 973.10 | 1227.32 | 2055.95 | 1372.51 | 0.81 | 1.43E-04 | 4.59E-03 |
| ENSMUSG00000030102 | Itpr1 | 2350.68 | 2898.26 | 2191.37 | 3718.55 | 3431.02 | 5839.39 | 0.80 | 1.82E-05 | 9.15E-04 |
| ENSMUSG00000020241 | Col6a2 | 165.18 | 271.89 | 273.68 | 299.29 | 539.73 | 399.00 | 0.80 | 2.41E-03 | 3.38E-02 |
| ENSMUSG00000026315 | Serpinb8 | 153.54 | 95.96 | 126.39 | 200.25 | 284.07 | 171.24 | 0.80 | 2.73E-03 | 3.70E-02 |
| ENSMUSG00000050332 | Amer1 | 188.48 | 235.33 | 214.77 | 256.23 | 372.84 | 481.19 | 0.80 | 8.31E-04 | 1.64E-02 |
| ENSMUSG00000041992 | Rapgef5 | 130.24 | 109.67 | 107.38 | 178.71 | 208.61 | 216.62 | 0.80 | 3.99E-04 | 9.82E-03 |
| ENSMUSG00000046404 | Yod1 | 490.25 | 427.26 | 419.08 | 732.08 | 727.04 | 866.49 | 0.80 | 9.06E-07 | 8.44E-05 |
| ENSMUSG00000022951 | Rcan1 | 1396.64 | 1368.59 | 1549.92 | 2019.69 | 2814.95 | 2675.67 | 0.80 | 8.85E-07 | 8.36E-05 |
| ENSMUSG00000038582 | Pptc7 | 884.15 | 1051.00 | 731.72 | 1485.70 | 1399.04 | 1744.11 | 0.80 | 9.72E-06 | 5.76E-04 |
| ENSMUSG00000061313 | Ddhd2 | 1206.05 | 879.65 | 973.10 | 1651.49 | 1446.09 | 2214.17 | 0.80 | 7.01E-06 | 4.45E-04 |
| ENSMUSG00000036446 | Lum | 128.12 | 189.64 | 161.55 | 187.33 | 355.09 | 287.69 | 0.80 | 3.32E-03 | 4.20E-02 |
| ENSMUSG00000024177 | Nme4 | 91.06 | 123.38 | 118.79 | 189.48 | 230.81 | 157.54 | 0.79 | 2.21E-03 | 3.22E-02 |
| ENSMUSG00000032875 | Arhgef17 | 209.66 | 342.72 | 293.64 | 475.85 | 573.46 | 416.12 | 0.79 | 2.47E-04 | 6.82E-03 |
| ENSMUSG00000044197 | Gpr146 | 4114.75 | 5306.43 | 4339.97 | 8352.21 | 6074.64 | 9373.84 | 0.79 | 9.54E-07 | 8.83E-05 |
| ENSMUSG00000025089 | Gfra1 | 6308.72 | 9649.82 | 5388.14 | 10959.72 | 12073.82 | 13875.82 | 0.79 | 1.47E-05 | 7.83E-04 |
| ENSMUSG00000039286 | Fndc3b | 954.04 | 1250.92 | 720.32 | 1112.12 | 1709.74 | 2226.16 | 0.79 | 6.06E-04 | 1.31E-02 |
| ENSMUSG00000008384 | Sertad1 | 402.37 | 275.32 | 325.00 | 781.61 | 508.66 | 443.52 | 0.79 | 8.58E-04 | 1.67E-02 |
| ENSMUSG00000055485 | Soga1 | 104.83 | 145.08 | 107.38 | 158.26 | 227.26 | 229.47 | 0.79 | 3.40E-03 | 4.26E-02 |
| ENSMUSG00000023150 | Ivns1abp | 3622.38 | 3397.49 | 3148.31 | 4886.66 | 5442.59 | 7193.06 | 0.79 | 4.42E-07 | 4.57E-05 |
| ENSMUSG00000052040 | Klf13 | 4510.77 | 4994.56 | 3684.27 | 8960.49 | 7619.27 | 6108.24 | 0.78 | 1.05E-06 | 9.52E-05 |
| ENSMUSG00000026028 | Trak2 | 1082.16 | 1488.54 | 980.70 | 1509.38 | 1830.47 | 2763.01 | 0.78 | 2.09E-04 | 6.00E-03 |
| ENSMUSG00000057230 | Aak1 | 196.95 | 233.05 | 185.31 | 300.37 | 310.70 | 443.52 | 0.78 | 2.63E-04 | 7.17E-03 |
| ENSMUSG00000049044 | Rapgef4 | 3188.25 | 6496.81 | 4789.46 | 7725.63 | 6139.44 | 10974.11 | 0.78 | 5.83E-04 | 1.27E-02 |
| ENSMUSG00000064337 | mt-Rnr1 | 62881.81 | 40900.07 | 43528.03 | 56784.92 | 77872.21 | 118038.66 | 0.78 | 3.35E-04 | 8.54E-03 |
| ENSMUSG00000024659 | Anxa1 | 112.24 | 162.22 | 132.09 | 213.17 | 289.40 | 193.50 | 0.78 | 2.39E-03 | 3.35E-02 |
| ENSMUSG00000043885 | Slc36a4 | 357.90 | 424.97 | 354.46 | 563.06 | 588.56 | 792.86 | 0.78 | 4.23E-05 | 1.80E-03 |
| ENSMUSG00000045414 | 1190002N15Rik | 1574.53 | 2006.05 | 1784.64 | 2760.39 | 2222.84 | 4196.31 | 0.78 | 6.62E-05 | 2.54E-03 |
| ENSMUSG00000037885 | Stk35 | 1021.81 | 925.34 | 950.29 | 1737.62 | 1218.83 | 2000.97 | 0.77 | 7.80E-06 | 4.86E-04 |
| ENSMUSG00000039789 | Zfp597 | 170.48 | 178.21 | 133.04 | 206.71 | 271.64 | 343.34 | 0.77 | 1.61E-03 | 2.60E-02 |
| ENSMUSG00000026131 | Dst | 327.19 | 327.87 | 265.13 | 416.64 | 466.94 | 685.83 | 0.77 | 5.77E-04 | 1.26E-02 |
| ENSMUSG00000021270 | Hsp90aa1 | 13606.43 | 11096.09 | 11533.67 | 22872.23 | 25045.13 | 13950.31 | 0.77 | 3.19E-05 | 1.44E-03 |
| ENSMUSG00000031799 | Tpm4 | 585.55 | 652.31 | 686.11 | 951.71 | 1293.40 | 1038.59 | 0.77 | 1.81E-06 | 1.45E-04 |
| ENSMUSG00000017929 | B4galt5 | 2147.38 | 2191.12 | 1833.11 | 3354.67 | 3188.68 | 3983.97 | 0.77 | 9.72E-09 | 1.70E-06 |
| ENSMUSG00000020532 | Acaca | 6038.71 | 5377.26 | 4914.90 | 9734.56 | 7463.91 | 10659.88 | 0.77 | 7.89E-07 | 7.60E-05 |
| ENSMUSG00000022462 | Slc38a2 | 3776.97 | 4696.39 | 3521.77 | 7430.65 | 6866.48 | 6162.18 | 0.77 | 1.32E-07 | 1.66E-05 |
| ENSMUSG00000020484 | Xbp1 | 11519.40 | 16634.43 | 14081.40 | 24897.30 | 18800.05 | 28276.50 | 0.77 | 6.50E-06 | 4.18E-04 |
| ENSMUSG00000037416 | Dmxl1 | 639.55 | 701.43 | 535.01 | 738.54 | 948.08 | 1503.51 | 0.77 | 4.90E-04 | 1.14E-02 |
| ENSMUSG00000066406 | Akap13 | 3224.25 | 3567.70 | 2953.50 | 5406.65 | 5333.40 | 5821.41 | 0.77 | 4.12E-10 | 1.18E-07 |
| ENSMUSG00000070407 | Hs3st3b1 | 4163.46 | 5005.98 | 4275.35 | 6369.13 | 6227.33 | 10222.35 | 0.76 | 1.96E-05 | 9.68E-04 |
| ENSMUSG00000017144 | Rnd3 | 724.26 | 831.66 | 714.62 | 1052.91 | 1528.65 | 1263.77 | 0.76 | 4.30E-06 | 2.97E-04 |
| ENSMUSG00000039704 | Lmbrd2 | 1188.05 | 1105.84 | 840.06 | 1347.89 | 1655.59 | 2298.08 | 0.76 | 1.79E-04 | 5.34E-03 |
| ENSMUSG00000039270 | Megf9 | 1531.12 | 1654.19 | 1109.94 | 2407.26 | 2680.01 | 2179.92 | 0.76 | 2.53E-06 | 1.88E-04 |
| ENSMUSG00000026890 | Lhx6 | 212.83 | 172.50 | 187.21 | 292.83 | 384.38 | 290.26 | 0.76 | 1.27E-04 | 4.24E-03 |
| ENSMUSG00000030103 | Bhlhe40 | 5675.52 | 5098.51 | 4778.06 | 9892.82 | 5174.50 | 11216.42 | 0.76 | 3.80E-04 | 9.41E-03 |
| ENSMUSG00000028527 | Ak4 | 4428.18 | 4074.93 | 3268.05 | 6005.24 | 5114.13 | 8741.10 | 0.75 | 3.23E-05 | 1.45E-03 |
| ENSMUSG00000033792 | Atp7a | 90.00 | 132.52 | 102.63 | 160.41 | 187.31 | 198.64 | 0.75 | 2.01E-03 | 3.00E-02 |
| ENSMUSG00000042595 | Fam199x | 336.72 | 333.58 | 319.30 | 479.08 | 482.92 | 703.81 | 0.75 | 1.26E-04 | 4.24E-03 |
| ENSMUSG00000041688 | Amot | 260.48 | 251.33 | 160.60 | 299.29 | 449.18 | 381.87 | 0.75 | 1.89E-03 | 2.87E-02 |
| ENSMUSG00000037606 | Osbpl5 | 116.48 | 115.38 | 127.34 | 156.11 | 240.57 | 207.20 | 0.75 | 3.17E-03 | 4.08E-02 |
| ENSMUSG00000005871 | Apc | 1422.06 | 1617.63 | 1208.77 | 1850.66 | 2026.66 | 3260.47 | 0.75 | 1.63E-04 | 5.00E-03 |
| ENSMUSG00000037110 | Ralgapa2 | 1689.95 | 2156.84 | 1420.68 | 2625.81 | 2351.56 | 3850.40 | 0.75 | 9.10E-05 | 3.25E-03 |
| ENSMUSG00000020580 | Rock2 | 835.45 | 1111.55 | 1022.51 | 1429.72 | 1561.49 | 1982.14 | 0.74 | 4.24E-06 | 2.94E-04 |
| ENSMUSG00000048758 | Rpl29 | 7375.00 | 7211.95 | 7590.92 | 13160.28 | 12119.10 | 11831.18 | 0.74 | 7.14E-13 | 4.10E-10 |
| ENSMUSG00000018906 | P4ha2 | 393.90 | 354.14 | 389.62 | 719.16 | 733.25 | 452.08 | 0.74 | 1.65E-04 | 5.05E-03 |
| ENSMUSG00000026421 | Csrp1 | 523.08 | 563.20 | 716.52 | 919.41 | 1448.75 | 643.87 | 0.74 | 3.95E-03 | 4.76E-02 |
| ENSMUSG00000032860 | P2ry2 | 1285.46 | 1423.43 | 971.20 | 2179.03 | 1802.95 | 2158.52 | 0.74 | 2.77E-06 | 2.03E-04 |
| ENSMUSG00000034158 | Lrrc58 | 3261.31 | 2598.95 | 2723.53 | 3819.75 | 4084.38 | 6407.91 | 0.74 | 4.34E-05 | 1.84E-03 |
| ENSMUSG00000064339 | mt-Rnr2 | 72890.21 | 63433.83 | 61568.34 | 72399.83 | 89487.08 | 168001.38 | 0.74 | 9.47E-04 | 1.79E-02 |
| ENSMUSG00000068566 | Myadm | 950.86 | 1113.84 | 932.23 | 1905.57 | 1801.18 | 1286.89 | 0.74 | 8.86E-06 | 5.36E-04 |
| ENSMUSG00000023025 | Larp4 | 2507.39 | 2577.25 | 2389.98 | 3397.73 | 3067.06 | 5962.68 | 0.73 | 2.67E-04 | 7.24E-03 |
| ENSMUSG00000071369 | Map3k5 | 2162.20 | 2321.35 | 1682.96 | 3731.47 | 3401.73 | 3111.49 | 0.73 | 4.47E-07 | 4.58E-05 |
| ENSMUSG00000020173 | Cobl | 636.38 | 910.49 | 743.13 | 1197.17 | 1181.55 | 1423.03 | 0.73 | 1.96E-05 | 9.68E-04 |
| ENSMUSG00000037458 | Azin1 | 4162.40 | 3938.98 | 3738.44 | 5235.47 | 6087.07 | 8323.27 | 0.73 | 9.49E-06 | 5.70E-04 |
| ENSMUSG00000042677 | Zc3h12a | 192.71 | 175.93 | 133.04 | 346.66 | 277.86 | 208.06 | 0.73 | 3.40E-03 | 4.26E-02 |
| ENSMUSG00000078866 | Zfp970 | 536.84 | 708.29 | 698.46 | 1157.34 | 757.22 | 1310.01 | 0.73 | 9.19E-04 | 1.75E-02 |
| ENSMUSG00000042599 | Kdm7a | 779.33 | 635.17 | 764.98 | 1005.54 | 1262.33 | 1345.11 | 0.73 | 4.80E-06 | 3.26E-04 |
| ENSMUSG00000041135 | Ripk2 | 313.42 | 337.01 | 298.39 | 418.79 | 567.25 | 583.94 | 0.73 | 6.64E-05 | 2.54E-03 |
| ENSMUSG00000063317 | Usp31 | 375.90 | 335.86 | 295.54 | 476.93 | 476.70 | 709.80 | 0.73 | 3.31E-04 | 8.46E-03 |
| ENSMUSG00000019189 | Rnf145 | 885.21 | 998.45 | 971.20 | 1255.31 | 1303.17 | 2148.24 | 0.72 | 2.82E-04 | 7.50E-03 |
| ENSMUSG00000027381 | Bcl2l11 | 188.48 | 212.49 | 180.56 | 286.37 | 282.29 | 388.72 | 0.72 | 5.42E-04 | 1.23E-02 |
| ENSMUSG00000056515 | Rab31 | 149.30 | 221.62 | 205.26 | 247.62 | 416.34 | 284.26 | 0.72 | 3.11E-03 | 4.04E-02 |
| ENSMUSG00000030556 | Lrrc28 | 1393.47 | 1085.28 | 1386.47 | 1940.02 | 1780.76 | 2647.42 | 0.72 | 1.91E-05 | 9.53E-04 |
| ENSMUSG00000034617 | Mtrr | 358.96 | 444.39 | 320.25 | 507.08 | 512.21 | 827.96 | 0.72 | 9.55E-04 | 1.80E-02 |
| ENSMUSG00000061175 | Fnip2 | 1478.18 | 1206.37 | 998.75 | 2010.00 | 2063.94 | 1970.15 | 0.71 | 2.60E-05 | 1.23E-03 |
| ENSMUSG00000040659 | Efhd2 | 2926.71 | 3501.44 | 3345.02 | 5272.08 | 4699.57 | 6060.29 | 0.71 | 1.20E-07 | 1.52E-05 |
| ENSMUSG00000001774 | Chordc1 | 1639.12 | 1538.81 | 1762.79 | 2507.39 | 2780.33 | 2813.52 | 0.71 | 3.81E-09 | 7.92E-07 |
| ENSMUSG00000024052 | Lpin2 | 11142.45 | 12848.53 | 10779.14 | 22498.65 | 19305.16 | 15193.54 | 0.71 | 3.98E-06 | 2.80E-04 |
| ENSMUSG00000064372 | mt-Tp | 2357.04 | 2339.63 | 2061.18 | 2443.87 | 3249.04 | 5377.03 | 0.71 | 1.16E-03 | 2.06E-02 |
| ENSMUSG00000041351 | Rap1gap | 171.54 | 228.48 | 315.50 | 401.57 | 375.50 | 396.43 | 0.71 | 1.26E-03 | 2.18E-02 |
| ENSMUSG00000072872 | Rybp | 338.84 | 431.83 | 279.39 | 459.71 | 619.63 | 635.31 | 0.71 | 3.75E-04 | 9.36E-03 |
| ENSMUSG00000056091 | St3gal5 | 4915.26 | 4944.29 | 3946.55 | 6345.44 | 6814.99 | 9425.22 | 0.71 | 9.24E-06 | 5.57E-04 |
| ENSMUSG00000028081 | Rps3a1 | 10767.61 | 11575.90 | 10315.40 | 18584.16 | 17867.94 | 16942.78 | 0.71 | 1.09E-10 | 3.71E-08 |
| ENSMUSG00000028252 | Ccnc | 609.91 | 550.63 | 611.99 | 694.40 | 1107.87 | 1090.82 | 0.71 | 1.33E-04 | 4.39E-03 |
| ENSMUSG00000007617 | Homer1 | 447.90 | 411.26 | 313.60 | 549.06 | 600.98 | 762.03 | 0.71 | 2.85E-04 | 7.53E-03 |
| ENSMUSG00000042680 | Garem1 | 1566.06 | 2100.87 | 1239.18 | 2426.64 | 2294.75 | 3274.17 | 0.71 | 1.80E-04 | 5.34E-03 |
| ENSMUSG00000037526 | Atg14 | 901.09 | 1152.68 | 884.72 | 1629.96 | 1484.26 | 1673.04 | 0.70 | 3.55E-06 | 2.52E-04 |
| ENSMUSG00000035451 | Foxa1 | 1465.47 | 1768.43 | 1243.93 | 2914.34 | 2609.00 | 1772.37 | 0.70 | 2.45E-04 | 6.77E-03 |
| ENSMUSG00000026678 | Rgs5 | 1016.51 | 987.03 | 1166.96 | 2105.82 | 1581.02 | 1465.84 | 0.70 | 4.39E-04 | 1.05E-02 |
| ENSMUSG00000038393 | Txnip | 2193.97 | 2800.01 | 2165.71 | 4161.03 | 2284.98 | 5174.96 | 0.70 | 3.65E-03 | 4.50E-02 |
| ENSMUSG00000090100 | Ttbk2 | 169.42 | 167.93 | 168.20 | 225.01 | 255.66 | 337.35 | 0.70 | 1.52E-03 | 2.49E-02 |
| ENSMUSG00000014599 | Csf1 | 418.25 | 623.75 | 454.24 | 721.32 | 939.20 | 761.18 | 0.70 | 1.74E-04 | 5.26E-03 |
| ENSMUSG00000031278 | Acsl4 | 2962.71 | 3021.64 | 2577.19 | 3917.72 | 4159.84 | 5779.45 | 0.70 | 9.81E-06 | 5.77E-04 |
| ENSMUSG00000041782 | Lad1 | 601.44 | 440.96 | 440.93 | 595.36 | 690.64 | 1109.65 | 0.69 | 2.82E-03 | 3.77E-02 |
| ENSMUSG00000036863 | Syde2 | 408.72 | 402.12 | 306.94 | 547.99 | 529.08 | 725.21 | 0.69 | 2.85E-04 | 7.53E-03 |
| ENSMUSG00000026208 | Des | 196.95 | 306.16 | 250.88 | 389.73 | 504.22 | 318.51 | 0.69 | 2.09E-03 | 3.09E-02 |
| ENSMUSG00000031885 | Cbfb | 885.21 | 975.61 | 792.54 | 1156.26 | 1168.23 | 1936.76 | 0.68 | 5.75E-04 | 1.26E-02 |
| ENSMUSG00000022533 | Atp13a3 | 6860.39 | 6898.93 | 5309.27 | 7352.06 | 8701.39 | 14555.66 | 0.68 | 7.86E-04 | 1.59E-02 |
| ENSMUSG00000048249 | Crebrf | 776.15 | 838.52 | 713.67 | 1346.82 | 1204.63 | 1183.29 | 0.68 | 5.08E-06 | 3.39E-04 |
| ENSMUSG00000062373 | Tmem65 | 285.89 | 382.70 | 261.33 | 416.64 | 451.85 | 619.90 | 0.68 | 1.34E-03 | 2.29E-02 |
| ENSMUSG00000049658 | Bdp1 | 630.03 | 579.19 | 485.60 | 695.48 | 798.94 | 1220.96 | 0.68 | 1.44E-03 | 2.40E-02 |
| ENSMUSG00000027253 | Lrp4 | 1375.47 | 1263.49 | 1198.32 | 1539.53 | 1515.33 | 3094.36 | 0.68 | 2.09E-03 | 3.09E-02 |
| ENSMUSG00000033964 | Zbtb41 | 743.32 | 807.67 | 724.12 | 866.66 | 1066.15 | 1710.72 | 0.68 | 1.60E-03 | 2.59E-02 |
| ENSMUSG00000018846 | Pank3 | 2863.17 | 2834.28 | 2191.37 | 3113.51 | 3808.30 | 5706.67 | 0.68 | 4.11E-04 | 1.00E-02 |
| ENSMUSG00000033658 | Ddx19b | 1000.63 | 1344.60 | 1093.78 | 1483.55 | 1348.44 | 2671.39 | 0.68 | 2.24E-03 | 3.25E-02 |
| ENSMUSG00000030499 | Kctd15 | 586.61 | 750.55 | 629.09 | 1237.01 | 986.25 | 921.29 | 0.68 | 1.25E-04 | 4.20E-03 |
| ENSMUSG00000045103 | Dmd | 681.91 | 804.25 | 660.45 | 932.33 | 1010.22 | 1484.68 | 0.68 | 4.84E-04 | 1.13E-02 |
| ENSMUSG00000022698 | Naa50 | 4790.31 | 4422.22 | 4326.67 | 5742.55 | 6597.50 | 9266.82 | 0.67 | 2.96E-05 | 1.35E-03 |
| ENSMUSG00000040738 | Ints8 | 721.09 | 744.84 | 618.64 | 1041.07 | 1046.62 | 1235.52 | 0.67 | 4.08E-06 | 2.84E-04 |
| ENSMUSG00000026558 | Uck2 | 713.68 | 782.54 | 588.23 | 958.17 | 779.41 | 1581.43 | 0.67 | 2.26E-03 | 3.25E-02 |
| ENSMUSG00000043019 | Edem3 | 1900.66 | 2145.42 | 1851.16 | 2267.31 | 2932.13 | 4186.89 | 0.67 | 3.42E-04 | 8.67E-03 |
| ENSMUSG00000042202 | Slc35e2 | 2649.28 | 1992.34 | 2259.79 | 3184.56 | 4439.47 | 3360.64 | 0.67 | 2.52E-05 | 1.19E-03 |
| ENSMUSG00000015882 | Lcorl | 357.90 | 477.52 | 371.56 | 466.16 | 616.96 | 833.95 | 0.67 | 2.92E-03 | 3.87E-02 |
| ENSMUSG00000039958 | Etfbkmt | 2565.63 | 3528.86 | 2936.40 | 5546.61 | 4881.55 | 3936.02 | 0.67 | 1.15E-04 | 3.96E-03 |
| ENSMUSG00000029672 | Fam3c | 1473.94 | 1462.27 | 1492.91 | 2154.26 | 2375.53 | 2511.28 | 0.67 | 2.68E-08 | 4.18E-06 |
| ENSMUSG00000021360 | Gcnt2 | 761.32 | 801.96 | 639.55 | 1013.07 | 1285.41 | 1193.56 | 0.67 | 7.73E-05 | 2.88E-03 |
| ENSMUSG00000041702 | Btbd7 | 406.60 | 480.95 | 333.55 | 528.61 | 621.40 | 783.44 | 0.67 | 5.71E-04 | 1.25E-02 |
| ENSMUSG00000034640 | Tiparp | 208.60 | 213.63 | 197.66 | 287.45 | 436.76 | 257.72 | 0.66 | 3.99E-03 | 4.80E-02 |
| ENSMUSG00000036499 | Eea1 | 1385.00 | 1598.21 | 1352.26 | 1736.55 | 1970.73 | 3134.60 | 0.66 | 5.27E-04 | 1.21E-02 |
| ENSMUSG00000042354 | Gnl3 | 873.56 | 1063.57 | 1151.75 | 1331.75 | 1197.53 | 2341.75 | 0.66 | 2.69E-03 | 3.67E-02 |
| ENSMUSG00000046096 | Mosmo | 563.32 | 557.49 | 536.91 | 691.17 | 833.57 | 1086.54 | 0.66 | 2.40E-04 | 6.68E-03 |
| ENSMUSG00000035181 | Heatr5a | 764.50 | 844.23 | 564.47 | 1027.07 | 1193.09 | 1202.13 | 0.66 | 1.21E-04 | 4.08E-03 |
| ENSMUSG00000022679 | Mpv17l | 1413.59 | 1188.09 | 1052.92 | 1675.18 | 1793.19 | 2282.67 | 0.65 | 2.73E-04 | 7.37E-03 |
| ENSMUSG00000040855 | Reps2 | 1486.65 | 1733.01 | 1419.73 | 1905.57 | 2483.83 | 2900.86 | 0.65 | 7.97E-05 | 2.92E-03 |
| ENSMUSG00000051236 | Msrb3 | 415.08 | 653.45 | 544.52 | 734.24 | 898.37 | 900.74 | 0.65 | 8.43E-04 | 1.65E-02 |
| ENSMUSG00000025006 | Sorbs1 | 1130.87 | 1116.12 | 812.50 | 1393.11 | 1454.08 | 1950.46 | 0.65 | 1.63E-04 | 5.00E-03 |
| ENSMUSG00000035614 | Togaram1 | 606.73 | 644.31 | 458.99 | 627.65 | 812.26 | 1239.80 | 0.65 | 3.56E-03 | 4.41E-02 |
| ENSMUSG00000019487 | Trip10 | 507.20 | 560.92 | 578.73 | 869.89 | 719.94 | 994.07 | 0.65 | 1.21E-04 | 4.08E-03 |
| ENSMUSG00000053175 | Bcl3 | 2185.50 | 2157.99 | 2121.05 | 4557.22 | 3133.64 | 2445.35 | 0.65 | 4.49E-04 | 1.07E-02 |
| ENSMUSG00000029246 | Ppat | 1284.40 | 1383.44 | 942.69 | 1627.81 | 1830.47 | 2191.91 | 0.65 | 1.79E-04 | 5.34E-03 |
| ENSMUSG00000020288 | Ahsa2 | 741.21 | 860.22 | 778.29 | 1227.32 | 1276.54 | 1220.96 | 0.65 | 9.98E-07 | 9.12E-05 |
| ENSMUSG00000036885 | Arhgef26 | 950.86 | 1911.23 | 1450.14 | 2489.08 | 2080.81 | 2179.92 | 0.65 | 3.18E-03 | 4.09E-02 |
| ENSMUSG00000078566 | Bnip3 | 5240.33 | 5510.92 | 5266.51 | 6589.83 | 5924.62 | 12553.83 | 0.65 | 2.27E-03 | 3.26E-02 |
| ENSMUSG00000025326 | Ube3a | 2270.21 | 2066.59 | 2027.92 | 2480.47 | 2989.83 | 4474.58 | 0.64 | 4.85E-04 | 1.13E-02 |
| ENSMUSG00000050627 | Gpd1l | 1694.19 | 1573.08 | 1448.24 | 2257.62 | 2065.71 | 3042.99 | 0.64 | 3.54E-05 | 1.58E-03 |
| ENSMUSG00000036698 | Ago2 | 1278.05 | 1388.01 | 1117.54 | 1590.13 | 1581.02 | 2735.61 | 0.64 | 1.19E-03 | 2.10E-02 |
| ENSMUSG00000037405 | Icam1 | 411.90 | 549.49 | 591.08 | 733.16 | 959.62 | 731.21 | 0.64 | 8.00E-04 | 1.61E-02 |
| ENSMUSG00000030465 | Psd3 | 1959.96 | 2083.73 | 1810.30 | 2880.96 | 2831.81 | 3422.29 | 0.64 | 3.88E-07 | 4.13E-05 |
| ENSMUSG00000031732 | Phlpp2 | 212.83 | 217.06 | 216.67 | 356.35 | 241.46 | 410.98 | 0.64 | 3.32E-03 | 4.20E-02 |
| ENSMUSG00000036990 | Otud4 | 1357.47 | 1499.97 | 1439.69 | 1893.73 | 1953.86 | 2853.76 | 0.64 | 8.85E-05 | 3.18E-03 |
| ENSMUSG00000037253 | Mex3c | 730.62 | 801.96 | 612.94 | 1016.30 | 1090.12 | 1234.66 | 0.64 | 2.76E-05 | 1.28E-03 |
| ENSMUSG00000043391 | 2510009E07Rik | 355.78 | 367.85 | 351.61 | 531.84 | 549.50 | 593.36 | 0.64 | 5.52E-05 | 2.24E-03 |
| ENSMUSG00000026623 | Lpgat1 | 9759.57 | 9720.65 | 8498.44 | 9982.17 | 11561.61 | 22040.69 | 0.64 | 3.14E-03 | 4.05E-02 |
| ENSMUSG00000002289 | Angptl4 | 4623.01 | 4354.81 | 4140.41 | 8034.62 | 5624.57 | 6768.38 | 0.64 | 3.69E-05 | 1.62E-03 |
| ENSMUSG00000003355 | Fkbp11 | 669.20 | 616.89 | 701.31 | 1029.22 | 1118.52 | 947.83 | 0.64 | 1.25E-05 | 6.87E-04 |
| ENSMUSG00000037234 | Hook3 | 1982.20 | 1825.55 | 1714.32 | 2270.54 | 2611.66 | 3705.70 | 0.64 | 1.82E-04 | 5.38E-03 |
| ENSMUSG00000078941 | Ak6 | 222.36 | 295.88 | 335.45 | 392.96 | 434.09 | 500.03 | 0.64 | 1.22E-03 | 2.14E-02 |
| ENSMUSG00000000056 | Narf | 2535.98 | 3575.70 | 2491.66 | 4065.22 | 3680.47 | 5622.76 | 0.64 | 6.53E-04 | 1.39E-02 |
| ENSMUSG00000045767 | B230219D22Rik | 1674.07 | 1994.62 | 1408.33 | 2225.32 | 2187.33 | 3471.95 | 0.64 | 6.91E-04 | 1.45E-02 |
| ENSMUSG00000034801 | Sos2 | 603.55 | 606.61 | 516.01 | 737.47 | 854.87 | 1085.68 | 0.64 | 1.94E-04 | 5.64E-03 |
| ENSMUSG00000034377 | Tulp4 | 1693.13 | 2136.28 | 1774.19 | 2315.75 | 2608.11 | 3765.63 | 0.63 | 3.12E-04 | 8.02E-03 |
| ENSMUSG00000040423 | Rc3h1 | 1361.70 | 1253.21 | 1134.65 | 1565.37 | 1899.71 | 2347.74 | 0.63 | 1.63E-04 | 5.00E-03 |
| ENSMUSG00000040396 | Abhd13 | 931.80 | 1117.26 | 978.80 | 1313.44 | 1365.31 | 2008.68 | 0.63 | 2.63E-04 | 7.17E-03 |
| ENSMUSG00000032498 | Mlh1 | 490.25 | 493.52 | 498.90 | 760.07 | 774.09 | 762.03 | 0.63 | 1.14E-05 | 6.37E-04 |
| ENSMUSG00000035184 | Fam124a | 516.73 | 570.06 | 611.99 | 994.77 | 814.92 | 821.97 | 0.63 | 8.08E-04 | 1.61E-02 |
| ENSMUSG00000025907 | Rb1cc1 | 1412.53 | 1827.83 | 1414.98 | 1817.29 | 2245.03 | 3139.74 | 0.63 | 8.04E-04 | 1.61E-02 |
| ENSMUSG00000035064 | Eef2k | 639.55 | 830.52 | 620.54 | 861.27 | 885.94 | 1485.53 | 0.63 | 2.61E-03 | 3.59E-02 |
| ENSMUSG00000024118 | Tedc2 | 4473.71 | 3129.02 | 3445.75 | 6157.04 | 5619.24 | 5293.98 | 0.63 | 6.41E-04 | 1.37E-02 |
| ENSMUSG00000029273 | Sult1d1 | 3296.25 | 3275.25 | 2949.70 | 3969.40 | 5155.85 | 5577.38 | 0.63 | 4.33E-04 | 1.04E-02 |
| ENSMUSG00000066232 | Ipo7 | 2575.16 | 3089.04 | 2450.80 | 3295.45 | 4018.69 | 5210.92 | 0.63 | 2.78E-04 | 7.47E-03 |
| ENSMUSG00000046982 | Tshz1 | 1181.69 | 1258.92 | 973.10 | 1645.03 | 1647.60 | 1973.58 | 0.63 | 2.47E-05 | 1.18E-03 |
| ENSMUSG00000028261 | Ndufaf4 | 1152.05 | 1008.74 | 1111.84 | 1320.98 | 1421.23 | 2301.51 | 0.62 | 1.06E-03 | 1.93E-02 |
| ENSMUSG00000022094 | Slc39a14 | 8298.33 | 10035.95 | 8566.86 | 13897.74 | 10358.76 | 17210.78 | 0.62 | 3.20E-04 | 8.20E-03 |
| ENSMUSG00000059495 | Arhgef12 | 7580.42 | 8999.80 | 7003.64 | 10604.45 | 11063.60 | 14669.53 | 0.62 | 2.76E-05 | 1.28E-03 |
| ENSMUSG00000024561 | Mbd1 | 2062.67 | 2582.96 | 2305.40 | 4530.31 | 2410.15 | 3763.92 | 0.62 | 3.19E-03 | 4.09E-02 |
| ENSMUSG00000037174 | Elf2 | 936.04 | 1202.94 | 1105.19 | 1459.86 | 1660.03 | 1870.83 | 0.62 | 2.48E-05 | 1.18E-03 |
| ENSMUSG00000039157 | Fam102a | 2010.79 | 1833.55 | 1597.44 | 2394.34 | 2452.76 | 3519.04 | 0.62 | 1.42E-04 | 4.58E-03 |
| ENSMUSG00000031328 | Flna | 1048.28 | 1256.64 | 1208.77 | 1907.72 | 2095.01 | 1400.77 | 0.62 | 3.00E-04 | 7.78E-03 |
| ENSMUSG00000029135 | Fosl2 | 572.85 | 801.96 | 714.62 | 1261.77 | 1114.08 | 837.38 | 0.62 | 1.70E-03 | 2.69E-02 |
| ENSMUSG00000056501 | Cebpb | 6721.68 | 6260.33 | 6528.49 | 14485.56 | 8168.76 | 7332.63 | 0.62 | 2.11E-03 | 3.11E-02 |
| ENSMUSG00000059325 | Hopx | 1120.28 | 940.19 | 912.28 | 1477.09 | 1305.83 | 1780.93 | 0.62 | 1.49E-04 | 4.73E-03 |
| ENSMUSG00000032114 | Slc37a4 | 8757.88 | 7006.32 | 7481.63 | 12213.95 | 10701.41 | 12755.04 | 0.62 | 1.18E-05 | 6.57E-04 |
| ENSMUSG00000042742 | Bmt2 | 372.72 | 363.28 | 389.62 | 529.68 | 527.30 | 668.70 | 0.62 | 1.87E-04 | 5.48E-03 |
| ENSMUSG00000035873 | Pawr | 676.62 | 686.58 | 548.32 | 1094.90 | 882.39 | 952.11 | 0.62 | 6.76E-05 | 2.57E-03 |
| ENSMUSG00000031503 | Col4a2 | 1122.40 | 1228.08 | 1054.82 | 2158.57 | 1842.01 | 1220.11 | 0.62 | 1.39E-03 | 2.33E-02 |
| ENSMUSG00000031540 | Kat6a | 974.16 | 1156.10 | 773.54 | 1185.33 | 1439.88 | 1809.18 | 0.61 | 1.24E-03 | 2.16E-02 |
| ENSMUSG00000040618 | Pck2 | 259.42 | 195.35 | 275.58 | 373.58 | 413.68 | 330.50 | 0.61 | 2.21E-03 | 3.22E-02 |
| ENSMUSG00000021112 | Mpp5 | 778.27 | 884.21 | 659.50 | 915.10 | 1209.07 | 1419.60 | 0.61 | 6.46E-04 | 1.38E-02 |
| ENSMUSG00000095115 | Itpripl2 | 374.84 | 424.97 | 370.61 | 422.02 | 680.88 | 681.55 | 0.61 | 2.37E-03 | 3.34E-02 |
| ENSMUSG00000034837 | Gnat1 | 1590.42 | 1904.37 | 1893.93 | 3131.81 | 2772.34 | 2325.48 | 0.61 | 7.29E-04 | 1.51E-02 |
| ENSMUSG00000025860 | Xiap | 5428.81 | 5341.84 | 4389.39 | 6632.89 | 7490.55 | 8967.14 | 0.61 | 6.88E-05 | 2.61E-03 |
| ENSMUSG00000037443 | Cep85 | 2636.58 | 3343.79 | 2264.54 | 3884.35 | 3568.62 | 5103.04 | 0.61 | 1.23E-03 | 2.15E-02 |
| ENSMUSG00000061024 | Rrs1 | 1497.24 | 1491.97 | 1656.36 | 2562.29 | 2066.60 | 2447.06 | 0.61 | 5.04E-06 | 3.38E-04 |
| ENSMUSG00000021097 | Clmn | 6667.68 | 8091.59 | 6238.65 | 9547.23 | 8314.35 | 14071.89 | 0.60 | 6.76E-04 | 1.43E-02 |
| ENSMUSG00000034574 | Daam1 | 2491.51 | 2847.99 | 2348.17 | 3372.97 | 3538.44 | 4776.82 | 0.60 | 7.81E-05 | 2.89E-03 |
| ENSMUSG00000057982 | Zfp809 | 661.79 | 957.33 | 694.66 | 1323.13 | 1032.41 | 1159.32 | 0.60 | 5.48E-04 | 1.24E-02 |
| ENSMUSG00000015656 | Hspa8 | 40807.63 | 43194.00 | 37868.10 | 58855.21 | 66221.83 | 59933.34 | 0.60 | 8.67E-08 | 1.16E-05 |
| ENSMUSG00000044026 | Slc35g1 | 1251.58 | 1189.23 | 995.90 | 2046.60 | 1680.45 | 1489.81 | 0.60 | 7.23E-05 | 2.73E-03 |
| ENSMUSG00000002416 | Ndufb2 | 248.83 | 431.83 | 358.26 | 519.99 | 482.03 | 572.81 | 0.60 | 2.95E-03 | 3.90E-02 |
| ENSMUSG00000029512 | Ulk1 | 6406.14 | 6064.98 | 5175.28 | 10335.30 | 8737.79 | 7674.26 | 0.60 | 1.46E-05 | 7.79E-04 |
| ENSMUSG00000047648 | Fbxo30 | 489.20 | 568.91 | 519.81 | 790.22 | 790.95 | 806.55 | 0.60 | 3.58E-05 | 1.59E-03 |
| ENSMUSG00000047466 | 8030462N17Rik | 314.48 | 305.02 | 282.24 | 370.35 | 471.38 | 520.58 | 0.60 | 1.23E-03 | 2.15E-02 |
| ENSMUSG00000030313 | Dennd5b | 3481.55 | 3790.47 | 2942.10 | 4066.29 | 4999.62 | 6382.23 | 0.60 | 4.71E-04 | 1.11E-02 |
| ENSMUSG00000025017 | Pik3ap1 | 2419.51 | 3697.94 | 2536.32 | 4045.84 | 3939.68 | 5084.21 | 0.60 | 5.74E-04 | 1.26E-02 |
| ENSMUSG00000042460 | C1galt1 | 1031.34 | 1022.44 | 688.96 | 1229.47 | 1136.28 | 1765.52 | 0.59 | 2.82E-03 | 3.77E-02 |
| ENSMUSG00000033060 | Lmo7 | 838.62 | 1067.00 | 896.12 | 1297.30 | 1336.01 | 1584.85 | 0.59 | 7.59E-05 | 2.85E-03 |
| ENSMUSG00000041570 | Camsap2 | 852.39 | 985.89 | 860.01 | 1065.83 | 1322.70 | 1670.48 | 0.59 | 6.46E-04 | 1.38E-02 |
| ENSMUSG00000052934 | Fbxo31 | 4758.54 | 4515.89 | 3643.41 | 7441.41 | 5207.34 | 6787.22 | 0.59 | 1.33E-04 | 4.40E-03 |
| ENSMUSG00000034610 | Tut4 | 588.73 | 627.18 | 548.32 | 712.70 | 812.26 | 1125.07 | 0.59 | 9.39E-04 | 1.78E-02 |
| ENSMUSG00000038866 | Zcchc2 | 1595.71 | 1996.91 | 1341.81 | 2833.59 | 2468.74 | 2108.00 | 0.59 | 6.55E-04 | 1.39E-02 |
| ENSMUSG00000021959 | Lats2 | 1131.93 | 1038.44 | 962.64 | 1300.52 | 1615.64 | 1777.50 | 0.58 | 1.03E-04 | 3.59E-03 |
| ENSMUSG00000048706 | Lurap1l | 3108.83 | 3383.78 | 3286.10 | 5999.86 | 5030.69 | 3591.82 | 0.58 | 4.38E-04 | 1.05E-02 |
| ENSMUSG00000020190 | Mknk2 | 10059.23 | 7466.70 | 6864.89 | 13517.71 | 13032.56 | 9829.35 | 0.58 | 6.30E-04 | 1.35E-02 |
| ENSMUSG00000034560 | Washc4 | 943.45 | 975.61 | 815.35 | 1112.12 | 1360.87 | 1599.41 | 0.58 | 2.78E-04 | 7.47E-03 |
| ENSMUSG00000070871 | Ccnyl1 | 237.19 | 234.19 | 239.47 | 328.36 | 300.05 | 429.82 | 0.57 | 3.43E-03 | 4.28E-02 |
| ENSMUSG00000038024 | Dennd4c | 1034.51 | 1304.62 | 942.69 | 1243.47 | 1599.66 | 2041.22 | 0.57 | 2.30E-03 | 3.28E-02 |
| ENSMUSG00000027947 | Il6ra | 2281.86 | 2129.43 | 1662.06 | 2953.10 | 3408.83 | 2669.68 | 0.57 | 5.67E-04 | 1.25E-02 |
| ENSMUSG00000001473 | Tubb6 | 648.03 | 564.34 | 603.43 | 1059.37 | 929.44 | 712.37 | 0.57 | 8.02E-04 | 1.61E-02 |
| ENSMUSG00000035529 | Prdm4 | 454.25 | 530.07 | 434.28 | 665.33 | 637.38 | 802.27 | 0.57 | 1.01E-03 | 1.87E-02 |
| ENSMUSG00000030287 | Itpr2 | 2401.51 | 2365.90 | 2362.42 | 3627.04 | 3310.29 | 3649.19 | 0.57 | 1.33E-06 | 1.14E-04 |
| ENSMUSG00000036934 | 4921524J17Rik | 286.95 | 282.17 | 250.88 | 342.36 | 456.29 | 416.98 | 0.57 | 3.86E-03 | 4.70E-02 |
| ENSMUSG00000007812 | Zfp655 | 1345.82 | 1372.02 | 1199.27 | 1735.47 | 1832.25 | 2233.87 | 0.57 | 4.23E-05 | 1.80E-03 |
| ENSMUSG00000032788 | Pdxk | 3763.21 | 4094.35 | 3673.82 | 4613.20 | 4559.31 | 7908.86 | 0.57 | 2.09E-03 | 3.09E-02 |
| ENSMUSG00000040651 | Fam208a | 1024.98 | 864.79 | 986.40 | 1052.91 | 1298.73 | 1906.79 | 0.57 | 3.88E-03 | 4.71E-02 |
| ENSMUSG00000049470 | Aff4 | 3053.77 | 3604.26 | 2910.74 | 4050.14 | 4468.76 | 5648.45 | 0.57 | 1.63E-04 | 5.00E-03 |
| ENSMUSG00000058013 | 43719 | 918.04 | 856.80 | 775.44 | 1270.38 | 1540.19 | 964.95 | 0.57 | 1.28E-03 | 2.20E-02 |
| ENSMUSG00000032271 | Nnmt | 2617.52 | 2943.95 | 2772.00 | 3441.87 | 3982.29 | 4911.25 | 0.57 | 8.78E-04 | 1.69E-02 |
| ENSMUSG00000070780 | Rbm47 | 4189.93 | 4151.47 | 3577.84 | 6536.00 | 5197.58 | 5903.60 | 0.57 | 2.91E-05 | 1.34E-03 |
| ENSMUSG00000020634 | Ubxn2a | 1305.58 | 1525.10 | 1251.53 | 1575.06 | 2095.01 | 2365.72 | 0.57 | 4.75E-04 | 1.11E-02 |
| ENSMUSG00000034832 | Tet3 | 1148.87 | 1109.27 | 903.73 | 1424.33 | 1444.31 | 1803.19 | 0.56 | 1.02E-03 | 1.88E-02 |
| ENSMUSG00000030935 | Acsm3 | 2160.09 | 2974.80 | 2051.68 | 3089.82 | 2856.67 | 4663.80 | 0.56 | 2.93E-03 | 3.89E-02 |
| ENSMUSG00000002897 | Il17ra | 1137.22 | 1140.11 | 1215.42 | 1669.80 | 1584.57 | 1902.51 | 0.56 | 2.78E-05 | 1.28E-03 |
| ENSMUSG00000040167 | Ikzf5 | 375.90 | 418.12 | 339.25 | 486.62 | 542.39 | 641.31 | 0.56 | 1.82E-03 | 2.80E-02 |
| ENSMUSG00000020841 | Cpd | 1354.29 | 1378.87 | 1430.19 | 1851.74 | 2129.63 | 2161.09 | 0.56 | 1.73E-05 | 8.79E-04 |
| ENSMUSG00000015305 | Sash1 | 1849.84 | 2004.91 | 1802.70 | 2512.77 | 2342.68 | 3485.65 | 0.56 | 3.53E-04 | 8.87E-03 |
| ENSMUSG00000048332 | Lhfp | 290.13 | 435.25 | 340.20 | 498.46 | 521.98 | 547.12 | 0.56 | 1.78E-03 | 2.76E-02 |
| ENSMUSG00000026669 | Mcm10 | 5433.04 | 6312.88 | 6001.08 | 10798.23 | 6431.50 | 8896.07 | 0.56 | 3.30E-03 | 4.20E-02 |
| ENSMUSG00000020170 | Frs2 | 672.38 | 768.83 | 604.38 | 894.65 | 838.00 | 1274.05 | 0.56 | 2.32E-03 | 3.29E-02 |
| ENSMUSG00000028572 | Hook1 | 2839.88 | 3065.05 | 2207.52 | 3055.37 | 3844.70 | 5029.41 | 0.56 | 1.74E-03 | 2.73E-02 |
| ENSMUSG00000004591 | Pkn2 | 1661.36 | 1665.61 | 1291.44 | 1748.39 | 2264.56 | 2772.42 | 0.56 | 1.18E-03 | 2.09E-02 |
| ENSMUSG00000049878 | Rlf | 627.91 | 967.61 | 696.56 | 1254.23 | 933.88 | 1176.44 | 0.55 | 2.79E-03 | 3.74E-02 |
| ENSMUSG00000042712 | Tceal9 | 463.78 | 349.57 | 503.65 | 551.22 | 735.03 | 647.30 | 0.55 | 3.11E-03 | 4.04E-02 |
| ENSMUSG00000035798 | Zdhhc17 | 373.78 | 405.55 | 382.02 | 573.82 | 496.23 | 633.60 | 0.55 | 8.37E-04 | 1.65E-02 |
| ENSMUSG00000071226 | Cecr2 | 852.39 | 721.99 | 538.81 | 1037.84 | 1155.81 | 904.16 | 0.55 | 2.30E-03 | 3.28E-02 |
| ENSMUSG00000026259 | Ngef | 1709.01 | 1895.24 | 1762.79 | 3330.98 | 1910.36 | 2628.58 | 0.55 | 1.89E-03 | 2.87E-02 |
| ENSMUSG00000035349 | #N/A | 2885.41 | 3590.55 | 2297.80 | 3320.21 | 4351.58 | 5182.67 | 0.55 | 2.58E-03 | 3.57E-02 |
| ENSMUSG00000042251 | Pm20d1 | 7831.37 | 9106.04 | 7713.50 | 12999.87 | 12562.07 | 10551.99 | 0.55 | 2.21E-05 | 1.08E-03 |
| ENSMUSG00000020262 | Adarb1 | 792.03 | 917.34 | 824.85 | 1269.30 | 1053.72 | 1387.07 | 0.55 | 2.96E-04 | 7.70E-03 |
| ENSMUSG00000074221 | Zfp568 | 691.44 | 775.69 | 634.79 | 1056.14 | 877.06 | 1143.05 | 0.55 | 9.06E-04 | 1.73E-02 |
| ENSMUSG00000003226 | Ranbp2 | 3153.30 | 3506.01 | 3310.81 | 3510.77 | 4393.31 | 6688.75 | 0.55 | 3.49E-03 | 4.34E-02 |
| ENSMUSG00000040565 | Btaf1 | 808.97 | 960.76 | 690.86 | 968.93 | 1169.12 | 1459.85 | 0.55 | 1.95E-03 | 2.92E-02 |
| ENSMUSG00000020482 | Ccdc117 | 1804.31 | 1683.89 | 1563.23 | 2282.38 | 2469.63 | 2639.71 | 0.55 | 2.44E-04 | 6.77E-03 |
| ENSMUSG00000032497 | Lrrfip2 | 1555.47 | 1587.93 | 1562.28 | 2464.32 | 2462.52 | 1955.60 | 0.55 | 8.02E-05 | 2.93E-03 |
| ENSMUSG00000018143 | Mafk | 1047.22 | 1037.30 | 1048.17 | 1651.49 | 1495.80 | 1432.45 | 0.55 | 1.28E-05 | 7.00E-04 |
| ENSMUSG00000030691 | Fchsd2 | 877.80 | 805.39 | 753.58 | 1162.72 | 1210.84 | 1184.15 | 0.55 | 2.77E-05 | 1.28E-03 |
| ENSMUSG00000020954 | Strn3 | 1122.40 | 1328.61 | 1163.15 | 1422.18 | 1762.12 | 2090.02 | 0.55 | 5.51E-04 | 1.24E-02 |
| ENSMUSG00000024122 | Pdpk1 | 1766.19 | 1951.21 | 1639.25 | 2097.20 | 2476.73 | 3233.07 | 0.54 | 7.49E-04 | 1.53E-02 |
| ENSMUSG00000040550 | Otud6b | 1740.78 | 1667.90 | 1439.69 | 1906.65 | 2301.85 | 2852.91 | 0.54 | 7.98E-04 | 1.61E-02 |
| ENSMUSG00000026014 | Raph1 | 2191.85 | 2715.48 | 2055.48 | 3430.03 | 3450.55 | 3252.76 | 0.54 | 1.35E-04 | 4.43E-03 |
| ENSMUSG00000030315 | Vgll4 | 1678.30 | 1887.24 | 1637.35 | 2545.07 | 2323.15 | 2702.21 | 0.54 | 5.73E-05 | 2.30E-03 |
| ENSMUSG00000030304 | Ergic2 | 1064.16 | 1322.89 | 1093.78 | 1331.75 | 1536.64 | 2193.62 | 0.54 | 3.25E-03 | 4.15E-02 |
| ENSMUSG00000026113 | Inpp4a | 980.51 | 998.45 | 863.81 | 1535.22 | 1181.55 | 1417.89 | 0.54 | 2.24E-04 | 6.28E-03 |
| ENSMUSG00000028410 | Dnaja1 | 14097.74 | 12725.15 | 14104.20 | 18208.42 | 21455.20 | 19824.80 | 0.54 | 2.25E-06 | 1.74E-04 |
| ENSMUSG00000052557 | Gan | 375.90 | 430.68 | 509.36 | 670.72 | 530.85 | 712.37 | 0.54 | 3.34E-03 | 4.21E-02 |
| ENSMUSG00000021870 | Slmap | 2993.41 | 3195.28 | 2700.72 | 3676.57 | 3926.37 | 5306.82 | 0.54 | 4.17E-04 | 1.02E-02 |
| ENSMUSG00000030059 | Tmf1 | 1118.16 | 1241.78 | 1101.39 | 1509.38 | 1451.42 | 2061.77 | 0.54 | 1.07E-03 | 1.96E-02 |
| ENSMUSG00000030704 | Rab6a | 2197.15 | 2081.45 | 2025.07 | 2553.68 | 3562.40 | 3031.86 | 0.54 | 1.30E-04 | 4.33E-03 |
| ENSMUSG00000039809 | Gabbr2 | 1449.59 | 1728.45 | 1322.80 | 1908.80 | 2112.76 | 2507.85 | 0.54 | 1.10E-03 | 1.99E-02 |
| ENSMUSG00000075232 | Amd1 | 1853.02 | 2383.04 | 1996.56 | 2562.29 | 2910.82 | 3569.56 | 0.54 | 5.68E-04 | 1.25E-02 |
| ENSMUSG00000041895 | Wipi1 | 894.74 | 1278.34 | 819.15 | 1275.76 | 1397.26 | 1656.78 | 0.53 | 3.25E-03 | 4.15E-02 |
| ENSMUSG00000032540 | Abhd5 | 1464.41 | 1451.99 | 1246.78 | 2053.06 | 1815.38 | 2156.81 | 0.53 | 6.52E-05 | 2.51E-03 |
| ENSMUSG00000038658 | Ric1 | 1270.64 | 1135.54 | 945.54 | 1379.12 | 1505.57 | 1962.44 | 0.53 | 1.69E-03 | 2.68E-02 |
| ENSMUSG00000050730 | Arhgap42 | 2804.94 | 3220.41 | 2399.48 | 3467.71 | 3713.32 | 4997.73 | 0.53 | 1.24E-03 | 2.16E-02 |
| ENSMUSG00000033671 | Cep350 | 1482.41 | 1558.23 | 1377.92 | 1729.01 | 2056.84 | 2595.19 | 0.53 | 8.16E-04 | 1.62E-02 |
| ENSMUSG00000034509 | Mad2l1bp | 518.84 | 518.65 | 598.68 | 870.96 | 757.22 | 738.06 | 0.53 | 1.13E-03 | 2.02E-02 |
| ENSMUSG00000025871 | 4833439L19Rik | 3682.74 | 4133.19 | 3696.63 | 5226.86 | 5123.01 | 6255.51 | 0.53 | 3.93E-05 | 1.71E-03 |
| ENSMUSG00000044795 | Cyb5d1 | 753.91 | 889.93 | 761.18 | 968.93 | 997.79 | 1500.09 | 0.53 | 3.46E-03 | 4.31E-02 |
| ENSMUSG00000041328 | Pcf11 | 638.50 | 597.47 | 554.97 | 839.74 | 895.71 | 846.80 | 0.53 | 4.79E-04 | 1.12E-02 |
| ENSMUSG00000027087 | Itgav | 884.15 | 932.20 | 828.65 | 1013.07 | 1110.53 | 1687.60 | 0.53 | 3.87E-03 | 4.71E-02 |
| ENSMUSG00000025862 | Stag2 | 2083.85 | 2079.16 | 1705.77 | 2420.18 | 2803.41 | 3231.36 | 0.53 | 2.94E-04 | 7.68E-03 |
| ENSMUSG00000028967 | Errfi1 | 45445.46 | 46078.55 | 39254.58 | 67298.93 | 69481.52 | 51568.12 | 0.53 | 1.14E-04 | 3.94E-03 |
| ENSMUSG00000031333 | Abcb7 | 1917.61 | 1752.44 | 1542.32 | 1998.16 | 2441.22 | 3054.12 | 0.52 | 2.14E-03 | 3.15E-02 |
| ENSMUSG00000039987 | Phtf2 | 332.48 | 353.00 | 376.31 | 497.39 | 466.94 | 562.53 | 0.52 | 1.71E-03 | 2.70E-02 |
| ENSMUSG00000031010 | Usp9x | 3505.90 | 4188.02 | 2776.75 | 4119.05 | 4819.41 | 6108.24 | 0.52 | 2.64E-03 | 3.62E-02 |
| ENSMUSG00000026339 | Ccdc93 | 697.79 | 766.55 | 607.24 | 814.98 | 960.51 | 1198.70 | 0.52 | 2.15E-03 | 3.15E-02 |
| ENSMUSG00000075254 | Heg1 | 530.49 | 507.22 | 535.96 | 595.36 | 777.64 | 887.04 | 0.52 | 3.69E-03 | 4.54E-02 |
| ENSMUSG00000017418 | Arl5b | 1345.82 | 1240.64 | 1090.93 | 1534.15 | 1718.62 | 2029.23 | 0.52 | 6.08E-04 | 1.32E-02 |
| ENSMUSG00000022181 | C6 | 9535.09 | 10535.18 | 8975.49 | 14124.91 | 16317.99 | 11278.06 | 0.52 | 1.33E-03 | 2.27E-02 |
| ENSMUSG00000040274 | Cdk6 | 1136.16 | 1039.58 | 1130.85 | 1326.36 | 1458.52 | 1962.44 | 0.52 | 1.02E-03 | 1.88E-02 |
| ENSMUSG00000021824 | Ap3m1 | 3310.01 | 3035.35 | 3033.33 | 3928.49 | 4748.39 | 4789.67 | 0.52 | 2.97E-04 | 7.71E-03 |
| ENSMUSG00000022774 | Ncbp2 | 1129.81 | 1012.16 | 1023.46 | 1381.27 | 1404.37 | 1757.81 | 0.52 | 2.12E-04 | 6.04E-03 |
| ENSMUSG00000057069 | Ero1lb | 4395.35 | 4054.36 | 3683.32 | 5814.68 | 6767.06 | 4835.05 | 0.52 | 3.07E-04 | 7.92E-03 |
| ENSMUSG00000019838 | Slc16a10 | 4549.95 | 5462.94 | 4143.26 | 6214.10 | 7115.04 | 6955.03 | 0.52 | 1.16E-04 | 3.98E-03 |
| ENSMUSG00000028522 | Mier1 | 1473.94 | 1445.13 | 1301.90 | 1776.38 | 2100.34 | 2168.79 | 0.52 | 1.62E-04 | 5.00E-03 |
| ENSMUSG00000063334 | Krr1 | 750.74 | 707.14 | 611.99 | 828.98 | 994.24 | 1137.91 | 0.52 | 1.28E-03 | 2.20E-02 |
| ENSMUSG00000045671 | Spred2 | 708.38 | 709.43 | 505.55 | 1030.30 | 846.88 | 875.05 | 0.52 | 2.32E-03 | 3.29E-02 |
| ENSMUSG00000066735 | Vkorc1l1 | 2162.20 | 2068.88 | 2008.91 | 2484.78 | 2829.15 | 3607.23 | 0.52 | 4.24E-04 | 1.03E-02 |
| ENSMUSG00000021133 | Susd6 | 2113.50 | 2616.09 | 1974.70 | 3132.89 | 3004.03 | 3441.13 | 0.52 | 2.93E-04 | 7.68E-03 |
| ENSMUSG00000044447 | Dock5 | 1047.22 | 1255.49 | 1023.46 | 1274.69 | 1457.63 | 2016.39 | 0.51 | 3.13E-03 | 4.05E-02 |
| ENSMUSG00000036197 | Gxylt1 | 825.92 | 1092.13 | 882.82 | 1098.13 | 1265.00 | 1627.66 | 0.51 | 2.97E-03 | 3.91E-02 |
| ENSMUSG00000025437 | Usp33 | 775.09 | 927.63 | 629.09 | 993.70 | 1051.94 | 1272.34 | 0.51 | 3.03E-03 | 3.97E-02 |
| ENSMUSG00000056216 | Cebpg | 3030.47 | 3615.68 | 3155.91 | 4251.47 | 4154.51 | 5551.70 | 0.51 | 3.62E-04 | 9.07E-03 |
| ENSMUSG00000026031 | Cflar | 4216.40 | 4201.73 | 4330.47 | 5157.96 | 6079.97 | 6911.37 | 0.51 | 1.04E-04 | 3.62E-03 |
| ENSMUSG00000037933 | Bicd2 | 836.50 | 948.19 | 792.54 | 1072.29 | 1128.29 | 1464.13 | 0.51 | 1.57E-03 | 2.55E-02 |
| ENSMUSG00000025234 | Arih1 | 2791.17 | 3060.48 | 2492.61 | 3419.26 | 3658.28 | 4793.09 | 0.51 | 7.64E-04 | 1.56E-02 |
| ENSMUSG00000027007 | Ssfa2 | 3390.49 | 3607.69 | 2724.48 | 4108.28 | 4152.73 | 5561.12 | 0.51 | 9.90E-04 | 1.85E-02 |
| ENSMUSG00000040661 | Rad54l2 | 1827.60 | 2078.02 | 1665.86 | 2329.75 | 2792.75 | 2796.40 | 0.51 | 1.55E-03 | 2.53E-02 |
| ENSMUSG00000051285 | Pcmtd1 | 4991.49 | 4465.63 | 4304.81 | 4949.10 | 6573.54 | 8040.72 | 0.51 | 1.87E-03 | 2.85E-02 |
| ENSMUSG00000018736 | Ndel1 | 1216.64 | 1579.93 | 1480.55 | 1960.48 | 1977.83 | 2141.39 | 0.51 | 1.51E-04 | 4.75E-03 |
| ENSMUSG00000030451 | Herc2 | 2368.68 | 2437.87 | 2116.30 | 3076.90 | 2966.75 | 3778.48 | 0.51 | 2.21E-04 | 6.22E-03 |
| ENSMUSG00000032307 | Ube2q2 | 1569.24 | 1649.62 | 1268.64 | 1762.38 | 1927.23 | 2673.96 | 0.50 | 3.77E-03 | 4.62E-02 |
| ENSMUSG00000028271 | Gtf2b | 772.97 | 929.91 | 949.34 | 1373.73 | 1116.75 | 1273.19 | 0.50 | 1.61E-03 | 2.60E-02 |
| ENSMUSG00000028300 | C9orf72 | 425.66 | 461.53 | 366.81 | 600.74 | 601.87 | 573.66 | 0.50 | 1.63E-03 | 2.62E-02 |
| ENSMUSG00000046711 | Hmga1 | 656.50 | 870.51 | 798.24 | 1114.27 | 1148.70 | 1032.60 | 0.50 | 5.32E-04 | 1.22E-02 |
| ENSMUSG00000034297 | Med13 | 1318.29 | 1080.71 | 1112.79 | 1268.23 | 1780.76 | 1922.20 | 0.50 | 3.24E-03 | 4.14E-02 |
| ENSMUSG00000022280 | Rnf19a | 1307.70 | 1376.59 | 1102.34 | 1656.88 | 1839.35 | 1857.99 | 0.50 | 1.83E-04 | 5.41E-03 |
| ENSMUSG00000020257 | Wdr82 | 1926.08 | 2123.71 | 1789.40 | 2522.46 | 2567.27 | 3159.43 | 0.50 | 2.37E-04 | 6.61E-03 |
| ENSMUSG00000021147 | Wdr37 | 511.43 | 646.60 | 468.49 | 714.86 | 748.34 | 832.24 | 0.50 | 3.13E-03 | 4.05E-02 |
| ENSMUSG00000075054 | Yae1d1 | 626.85 | 712.86 | 547.37 | 731.01 | 908.13 | 1023.18 | 0.50 | 3.08E-03 | 4.01E-02 |
| ENSMUSG00000027177 | Hipk3 | 4016.28 | 4433.64 | 3362.13 | 4691.79 | 4997.84 | 6967.88 | 0.50 | 2.37E-03 | 3.34E-02 |
| ENSMUSG00000023852 | Chd1 | 1173.22 | 1400.58 | 1137.50 | 1533.07 | 1754.13 | 1941.04 | 0.50 | 5.23E-04 | 1.20E-02 |
| ENSMUSG00000020255 | D10Wsu102e | 768.74 | 968.75 | 830.55 | 1061.52 | 1177.11 | 1376.79 | 0.49 | 1.69E-03 | 2.68E-02 |
| ENSMUSG00000049550 | Clip1 | 2216.21 | 2750.89 | 2109.64 | 2812.06 | 3278.33 | 3861.53 | 0.49 | 1.14E-03 | 2.03E-02 |
| ENSMUSG00000032041 | Tirap | 1539.59 | 1857.54 | 1417.83 | 2171.49 | 2075.48 | 2523.27 | 0.49 | 8.06E-04 | 1.61E-02 |
| ENSMUSG00000031596 | Slc7a2 | 20516.58 | 20848.73 | 13939.80 | 25605.70 | 28902.25 | 23243.67 | 0.49 | 2.45E-03 | 3.42E-02 |
| ENSMUSG00000041417 | Pik3r1 | 4600.77 | 5273.30 | 4275.35 | 6217.33 | 5512.72 | 8158.87 | 0.49 | 1.66E-03 | 2.66E-02 |
| ENSMUSG00000022401 | Xpnpep3 | 1071.57 | 1100.13 | 1117.54 | 1417.87 | 1273.87 | 1926.48 | 0.49 | 2.33E-03 | 3.29E-02 |
| ENSMUSG00000001542 | Ell2 | 3172.36 | 3109.60 | 2555.33 | 3940.33 | 4847.82 | 3605.52 | 0.49 | 1.52E-03 | 2.49E-02 |
| ENSMUSG00000020130 | Tbc1d15 | 1791.60 | 2172.84 | 1817.90 | 2189.79 | 2681.79 | 3227.93 | 0.49 | 1.75E-03 | 2.73E-02 |
| ENSMUSG00000003518 | Dusp3 | 1770.42 | 2175.12 | 1523.31 | 2288.84 | 2402.16 | 2968.50 | 0.49 | 2.14E-03 | 3.15E-02 |
| ENSMUSG00000021669 | Col4a3bp | 1967.37 | 2030.04 | 1611.69 | 2282.38 | 2478.50 | 3084.94 | 0.48 | 1.19E-03 | 2.09E-02 |
| ENSMUSG00000029053 | Prkcz | 1455.94 | 1517.10 | 1335.16 | 2013.23 | 1970.73 | 2041.22 | 0.48 | 5.94E-05 | 2.35E-03 |
| ENSMUSG00000025198 | Erlin1 | 2566.69 | 2788.59 | 2510.67 | 3548.45 | 3720.42 | 3717.69 | 0.48 | 1.73E-05 | 8.79E-04 |
| ENSMUSG00000040446 | Rprd1a | 571.79 | 596.33 | 536.91 | 718.09 | 815.81 | 845.08 | 0.48 | 3.11E-03 | 4.04E-02 |
| ENSMUSG00000041235 | Chd7 | 1132.99 | 1144.68 | 1176.46 | 1670.87 | 1602.33 | 1549.75 | 0.48 | 1.36E-04 | 4.44E-03 |
| ENSMUSG00000022811 | Zfp148 | 1319.35 | 1362.88 | 1125.14 | 1457.71 | 1788.75 | 2063.48 | 0.48 | 2.48E-03 | 3.44E-02 |
| ENSMUSG00000031790 | Mmp15 | 5419.28 | 5131.64 | 4729.59 | 7711.64 | 6539.80 | 7073.19 | 0.48 | 4.96E-05 | 2.05E-03 |
| ENSMUSG00000021987 | Mtmr6 | 1128.75 | 1125.26 | 1053.87 | 1173.49 | 1681.33 | 1752.67 | 0.48 | 2.69E-03 | 3.67E-02 |
| ENSMUSG00000027104 | Atf2 | 1135.10 | 1177.81 | 1077.63 | 1258.54 | 1612.09 | 1846.86 | 0.48 | 2.25E-03 | 3.25E-02 |
| ENSMUSG00000028991 | Mtor | 2415.27 | 3063.91 | 2413.74 | 3502.16 | 3238.39 | 4243.40 | 0.48 | 1.10E-03 | 1.99E-02 |
| ENSMUSG00000027598 | Itch | 2528.57 | 2935.96 | 2665.56 | 3283.61 | 3565.95 | 4461.74 | 0.48 | 1.33E-03 | 2.27E-02 |
| ENSMUSG00000048756 | Foxo3 | 3658.38 | 3922.99 | 3038.08 | 5590.75 | 4309.86 | 4856.45 | 0.47 | 1.13E-03 | 2.02E-02 |
| ENSMUSG00000040181 | Fmo1 | 6246.25 | 6252.33 | 5811.97 | 8200.41 | 7182.51 | 10045.97 | 0.47 | 7.39E-04 | 1.52E-02 |
| ENSMUSG00000032244 | Fem1b | 990.04 | 917.34 | 1002.56 | 1283.30 | 1142.49 | 1613.97 | 0.47 | 2.15E-03 | 3.15E-02 |
| ENSMUSG00000039835 | Nhsl1 | 578.14 | 606.61 | 550.22 | 680.41 | 901.03 | 824.54 | 0.47 | 2.75E-03 | 3.71E-02 |
| ENSMUSG00000024074 | Crim1 | 1048.28 | 1071.57 | 990.20 | 1403.88 | 1368.86 | 1542.90 | 0.47 | 2.81E-04 | 7.50E-03 |
| ENSMUSG00000017291 | Taok1 | 1777.84 | 1832.40 | 1578.43 | 1941.10 | 2318.71 | 2938.53 | 0.47 | 3.82E-03 | 4.66E-02 |
| ENSMUSG00000020315 | Sptbn1 | 3914.63 | 4360.53 | 3610.15 | 4293.45 | 5281.91 | 6904.52 | 0.47 | 3.90E-03 | 4.72E-02 |
| ENSMUSG00000020661 | Dnmt3a | 1076.87 | 1008.74 | 895.17 | 1315.60 | 1279.20 | 1534.34 | 0.47 | 1.11E-03 | 2.00E-02 |
| ENSMUSG00000028878 | Fam76a | 743.32 | 905.92 | 719.37 | 1052.91 | 985.36 | 1240.66 | 0.47 | 2.80E-03 | 3.74E-02 |
| ENSMUSG00000036097 | Slf2 | 735.91 | 645.45 | 612.94 | 819.29 | 998.68 | 938.41 | 0.47 | 2.30E-03 | 3.28E-02 |
| ENSMUSG00000029004 | Kmt2e | 1156.28 | 1423.43 | 1152.70 | 1611.66 | 1568.59 | 1977.86 | 0.47 | 2.30E-03 | 3.28E-02 |
| ENSMUSG00000028132 | Tmem56 | 8915.65 | 8657.08 | 7947.27 | 8956.18 | 12082.70 | 14238.86 | 0.47 | 2.78E-03 | 3.73E-02 |
| ENSMUSG00000037857 | Nufip2 | 918.04 | 724.28 | 740.28 | 1075.52 | 1060.82 | 1155.89 | 0.47 | 2.00E-03 | 2.99E-02 |
| ENSMUSG00000036391 | Sec24a | 4236.52 | 3966.40 | 3406.79 | 4598.13 | 5141.65 | 6289.76 | 0.47 | 1.35E-03 | 2.29E-02 |
| ENSMUSG00000038859 | Baiap2l1 | 1276.99 | 1172.10 | 1024.41 | 1671.95 | 1706.19 | 1417.04 | 0.47 | 1.13E-03 | 2.02E-02 |
| ENSMUSG00000048578 | Mlec | 10565.36 | 10213.02 | 10511.16 | 14728.87 | 14498.18 | 13939.18 | 0.46 | 2.38E-06 | 1.81E-04 |
| ENSMUSG00000029992 | Gfpt1 | 1417.82 | 1527.38 | 1346.56 | 1550.29 | 2079.03 | 2284.38 | 0.46 | 2.68E-03 | 3.66E-02 |
| ENSMUSG00000030201 | Lrp6 | 4360.41 | 4827.77 | 3810.66 | 5338.83 | 5430.16 | 7147.68 | 0.46 | 1.69E-03 | 2.68E-02 |
| ENSMUSG00000049516 | Spty2d1 | 785.68 | 869.36 | 890.42 | 1009.84 | 1083.90 | 1412.75 | 0.46 | 3.47E-03 | 4.32E-02 |
| ENSMUSG00000016756 | Cmah | 11102.21 | 11382.83 | 9385.06 | 12729.64 | 13636.20 | 17537.00 | 0.46 | 2.08E-03 | 3.09E-02 |
| ENSMUSG00000022629 | Kif21a | 2928.82 | 3179.29 | 2734.93 | 3475.24 | 4238.84 | 4460.02 | 0.46 | 6.97E-04 | 1.46E-02 |
| ENSMUSG00000035354 | Uvrag | 1674.07 | 1955.78 | 1586.03 | 2428.79 | 2048.85 | 2702.21 | 0.46 | 2.27E-03 | 3.26E-02 |
| ENSMUSG00000102869 | 2900097C17Rik | 1718.54 | 1577.65 | 1520.46 | 2059.52 | 2040.86 | 2522.41 | 0.46 | 3.79E-03 | 4.63E-02 |
| ENSMUSG00000022507 | 1810013L24Rik | 2048.91 | 1974.06 | 1722.88 | 2781.92 | 3032.44 | 2087.45 | 0.46 | 2.61E-03 | 3.59E-02 |
| ENSMUSG00000037720 | Tmem33 | 2874.82 | 2713.19 | 2544.88 | 3369.74 | 3864.23 | 3930.88 | 0.46 | 1.48E-04 | 4.70E-03 |
| ENSMUSG00000039879 | Heca | 1804.31 | 1978.63 | 1587.93 | 2204.86 | 2324.93 | 2840.07 | 0.46 | 1.19E-03 | 2.09E-02 |
| ENSMUSG00000018209 | Stk4 | 873.56 | 982.46 | 902.78 | 1073.36 | 1430.11 | 1278.33 | 0.46 | 1.83E-03 | 2.81E-02 |
| ENSMUSG00000033396 | Spg11 | 829.09 | 668.30 | 806.80 | 1001.23 | 1020.87 | 1137.91 | 0.46 | 2.21E-03 | 3.22E-02 |
| ENSMUSG00000059811 | Atl2 | 4415.47 | 4341.11 | 3871.48 | 5033.07 | 5042.23 | 7233.30 | 0.46 | 2.72E-03 | 3.69E-02 |
| ENSMUSG00000041439 | Mfsd6 | 698.85 | 735.70 | 695.61 | 923.72 | 1123.85 | 871.63 | 0.45 | 3.69E-03 | 4.54E-02 |
| ENSMUSG00000057406 | Nsd2 | 957.21 | 1024.73 | 898.02 | 1309.14 | 1263.22 | 1371.66 | 0.45 | 4.55E-04 | 1.08E-02 |
| ENSMUSG00000060904 | Arl1 | 5688.23 | 5360.12 | 5425.21 | 6389.58 | 7715.14 | 8448.27 | 0.45 | 5.26E-04 | 1.21E-02 |
| ENSMUSG00000050697 | Prkaa1 | 587.67 | 634.03 | 585.38 | 726.70 | 849.54 | 895.60 | 0.45 | 1.93E-03 | 2.92E-02 |
| ENSMUSG00000027236 | Eif3j1 | 2903.41 | 2351.05 | 3130.26 | 3117.81 | 3851.80 | 4495.99 | 0.45 | 3.95E-03 | 4.76E-02 |
| ENSMUSG00000005483 | Dnajb1 | 1956.78 | 1718.16 | 1729.53 | 2456.79 | 2877.97 | 2053.20 | 0.45 | 3.59E-03 | 4.44E-02 |
| ENSMUSG00000032525 | Nktr | 1640.18 | 1592.50 | 1763.74 | 2263.00 | 2261.01 | 2300.65 | 0.45 | 6.60E-04 | 1.39E-02 |
| ENSMUSG00000022965 | Ifngr2 | 1031.34 | 972.18 | 982.60 | 1388.81 | 1487.81 | 1196.99 | 0.45 | 8.46E-04 | 1.65E-02 |
| ENSMUSG00000048874 | Phf3 | 2203.50 | 2725.76 | 2219.88 | 2944.48 | 3321.83 | 3477.95 | 0.45 | 1.22E-03 | 2.14E-02 |
| ENSMUSG00000050390 | C77080 | 7898.08 | 7394.73 | 6650.13 | 10515.09 | 8391.58 | 11004.93 | 0.45 | 7.19E-04 | 1.49E-02 |
| ENSMUSG00000014077 | Chp1 | 15538.86 | 14229.69 | 15113.41 | 18773.64 | 17717.03 | 24632.45 | 0.45 | 1.55E-03 | 2.53E-02 |
| ENSMUSG00000067851 | Arfgef1 | 3954.86 | 4761.51 | 3615.85 | 4642.27 | 5556.21 | 6592.00 | 0.45 | 3.47E-03 | 4.32E-02 |
| ENSMUSG00000101517 | 4732465J04Rik | 920.15 | 1022.44 | 887.57 | 1191.79 | 1242.80 | 1414.47 | 0.44 | 3.09E-03 | 4.03E-02 |
| ENSMUSG00000045962 | Wnk1 | 4311.70 | 5043.68 | 3983.62 | 5346.36 | 6123.46 | 6669.06 | 0.44 | 1.40E-03 | 2.34E-02 |
| ENSMUSG00000027506 | Tpd52 | 983.69 | 862.51 | 883.77 | 1185.33 | 1231.26 | 1295.45 | 0.44 | 6.37E-04 | 1.36E-02 |
| ENSMUSG00000002881 | Nab1 | 3362.96 | 4118.34 | 3130.26 | 4412.96 | 4837.16 | 5167.26 | 0.44 | 1.08E-03 | 1.96E-02 |
| ENSMUSG00000042772 | Smg7 | 1640.18 | 1598.21 | 1775.14 | 1934.64 | 2171.35 | 2700.50 | 0.44 | 3.35E-03 | 4.22E-02 |
| ENSMUSG00000052151 | Plpp2 | 786.74 | 1061.29 | 830.55 | 1207.94 | 1256.12 | 1167.88 | 0.44 | 3.32E-03 | 4.20E-02 |
| ENSMUSG00000021285 | Ppp1r13b | 649.08 | 817.96 | 806.80 | 1014.15 | 1065.26 | 1003.48 | 0.44 | 1.84E-03 | 2.82E-02 |
| ENSMUSG00000019944 | Rhobtb1 | 2892.82 | 2529.26 | 2071.63 | 3139.35 | 3620.11 | 3394.04 | 0.44 | 1.85E-03 | 2.83E-02 |
| ENSMUSG00000000804 | Usp32 | 1452.76 | 1675.90 | 1417.83 | 1804.37 | 2122.53 | 2230.44 | 0.44 | 1.08E-03 | 1.96E-02 |
| ENSMUSG00000043940 | Wdfy3 | 2057.38 | 2317.92 | 1947.14 | 2491.24 | 2582.37 | 3485.65 | 0.44 | 4.07E-03 | 4.87E-02 |
| ENSMUSG00000027808 | Serp1 | 17718.00 | 15410.92 | 15925.91 | 20494.03 | 22622.55 | 23296.76 | 0.44 | 1.70E-04 | 5.16E-03 |
| ENSMUSG00000073700 | Klhl21 | 4088.28 | 3731.07 | 3725.14 | 5223.63 | 4569.96 | 5835.11 | 0.44 | 4.81E-04 | 1.12E-02 |
| ENSMUSG00000038708 | Golga4 | 4515.00 | 5560.04 | 4374.18 | 5479.86 | 6619.70 | 7453.35 | 0.44 | 2.71E-03 | 3.68E-02 |
| ENSMUSG00000044252 | Osbpl1a | 5047.61 | 5714.27 | 4837.93 | 6822.37 | 6524.71 | 7762.45 | 0.44 | 4.23E-04 | 1.03E-02 |
| ENSMUSG00000049323 | Smcr8 | 1448.53 | 1904.37 | 1652.55 | 2256.54 | 2304.51 | 2209.89 | 0.44 | 1.89E-03 | 2.87E-02 |
| ENSMUSG00000087370 | Tmem170b | 1202.87 | 1033.87 | 969.30 | 1278.99 | 1394.60 | 1661.06 | 0.44 | 4.04E-03 | 4.84E-02 |
| ENSMUSG00000027690 | Slc2a2 | 15752.75 | 12678.31 | 13344.92 | 17502.18 | 17928.31 | 21056.04 | 0.44 | 8.86E-04 | 1.70E-02 |
| ENSMUSG00000029104 | Htt | 1499.35 | 1671.33 | 1319.95 | 1867.89 | 1929.01 | 2268.11 | 0.43 | 3.13E-03 | 4.05E-02 |
| ENSMUSG00000043998 | Mgat2 | 4088.28 | 3863.58 | 3479.01 | 4665.96 | 4799.88 | 5974.67 | 0.43 | 1.50E-03 | 2.47E-02 |
| ENSMUSG00000034109 | Golim4 | 3332.25 | 3397.49 | 2970.61 | 3981.24 | 4224.64 | 4893.27 | 0.43 | 1.37E-03 | 2.31E-02 |
| ENSMUSG00000033161 | Atp1a1 | 22310.30 | 23505.94 | 20092.93 | 28806.41 | 27277.73 | 32729.68 | 0.43 | 2.85E-04 | 7.53E-03 |
| ENSMUSG00000050144 | Slc25a44 | 6738.62 | 6873.80 | 5496.48 | 8954.03 | 8263.75 | 8523.62 | 0.43 | 1.36E-03 | 2.31E-02 |
| ENSMUSG00000041698 | Slco1a1 | 18955.82 | 19271.08 | 17418.81 | 22102.46 | 22467.20 | 30344.26 | 0.43 | 3.36E-03 | 4.23E-02 |
| ENSMUSG00000038485 | Socs7 | 1443.23 | 1217.79 | 1040.57 | 1710.71 | 1644.05 | 1622.53 | 0.43 | 3.50E-03 | 4.34E-02 |
| ENSMUSG00000070738 | Dgkd | 1209.22 | 1234.93 | 1140.35 | 1611.66 | 1414.13 | 1791.20 | 0.43 | 1.41E-03 | 2.36E-02 |
| ENSMUSG00000079215 | Zfp664 | 1700.54 | 1857.54 | 1665.86 | 2033.68 | 2256.57 | 2721.05 | 0.43 | 2.34E-03 | 3.31E-02 |
| ENSMUSG00000020021 | Fgd6 | 2551.87 | 1929.51 | 1893.93 | 2603.20 | 3083.04 | 2870.03 | 0.42 | 3.37E-03 | 4.25E-02 |
| ENSMUSG00000060657 | Marf1 | 3768.50 | 3944.69 | 3270.90 | 4523.85 | 4741.29 | 5471.21 | 0.42 | 9.80E-04 | 1.84E-02 |
| ENSMUSG00000020594 | Pum2 | 2366.57 | 2652.64 | 2463.15 | 3001.54 | 3281.00 | 3747.65 | 0.42 | 8.24E-04 | 1.63E-02 |
| ENSMUSG00000052144 | Ppp4r2 | 1290.76 | 1401.72 | 1316.15 | 1472.78 | 1815.38 | 2083.17 | 0.42 | 3.95E-03 | 4.76E-02 |
| ENSMUSG00000055531 | Cpsf6 | 1242.05 | 1190.38 | 1263.89 | 1551.37 | 1449.64 | 1953.88 | 0.42 | 3.26E-03 | 4.16E-02 |
| ENSMUSG00000037580 | Gch1 | 8711.29 | 8302.93 | 7804.73 | 11456.03 | 10358.76 | 11457.87 | 0.42 | 1.18E-04 | 4.00E-03 |
| ENSMUSG00000021578 | Ccdc127 | 900.04 | 921.91 | 941.74 | 1172.41 | 1201.08 | 1330.56 | 0.42 | 8.80E-04 | 1.69E-02 |
| ENSMUSG00000035247 | Hectd1 | 7473.48 | 8860.42 | 6721.40 | 9419.12 | 9685.87 | 11778.09 | 0.42 | 2.38E-03 | 3.35E-02 |
| ENSMUSG00000032803 | Cdv3 | 6014.36 | 6370.00 | 5879.44 | 7096.90 | 7689.39 | 9675.23 | 0.42 | 1.63E-03 | 2.63E-02 |
| ENSMUSG00000030870 | Ubfd1 | 2559.28 | 2426.45 | 3040.93 | 3420.34 | 3742.61 | 3584.97 | 0.42 | 9.84E-04 | 1.84E-02 |
| ENSMUSG00000022391 | Rangap1 | 4301.11 | 4054.36 | 4046.33 | 5312.99 | 5345.82 | 5942.99 | 0.42 | 2.03E-04 | 5.88E-03 |
| ENSMUSG00000062797 | Hikeshi | 905.33 | 801.96 | 891.37 | 1145.50 | 1237.48 | 1095.96 | 0.42 | 1.58E-03 | 2.56E-02 |
| ENSMUSG00000034066 | Farp2 | 1023.92 | 1095.56 | 932.23 | 1317.75 | 1282.75 | 1480.40 | 0.42 | 1.85E-03 | 2.83E-02 |
| ENSMUSG00000057637 | Prdm2 | 1461.23 | 1977.49 | 1859.72 | 2433.10 | 2300.07 | 2351.17 | 0.42 | 3.38E-03 | 4.25E-02 |
| ENSMUSG00000040213 | Kyat3 | 10556.89 | 10097.64 | 9576.07 | 13568.31 | 15566.10 | 11249.81 | 0.42 | 1.64E-03 | 2.63E-02 |
| ENSMUSG00000041763 | Tpp2 | 1516.30 | 1733.01 | 1518.56 | 1856.05 | 2022.22 | 2477.03 | 0.42 | 3.74E-03 | 4.59E-02 |
| ENSMUSG00000021748 | Pdhb | 9282.02 | 8018.48 | 8447.13 | 12118.14 | 10815.04 | 11400.50 | 0.42 | 1.87E-04 | 5.48E-03 |
| ENSMUSG00000024811 | Tnks2 | 2522.22 | 2464.15 | 2177.11 | 2720.55 | 3159.38 | 3665.46 | 0.41 | 2.61E-03 | 3.59E-02 |
| ENSMUSG00000021690 | Jmy | 1296.05 | 1457.70 | 1200.22 | 1552.45 | 1795.85 | 1917.92 | 0.41 | 2.43E-03 | 3.39E-02 |
| ENSMUSG00000019861 | Gopc | 1571.36 | 1542.23 | 1574.63 | 1877.58 | 2116.31 | 2250.13 | 0.41 | 3.93E-03 | 4.75E-02 |
| ENSMUSG00000060143 | Gm10076 | 2043.61 | 2666.35 | 2820.46 | 3410.65 | 3380.42 | 3219.37 | 0.41 | 2.89E-03 | 3.84E-02 |
| ENSMUSG00000022136 | Dnajc3 | 15732.63 | 17932.19 | 14162.17 | 21712.74 | 23741.96 | 18049.87 | 0.41 | 2.71E-03 | 3.68E-02 |
| ENSMUSG00000041471 | Shld2 | 1539.59 | 1515.96 | 1320.90 | 2049.83 | 1968.95 | 1792.06 | 0.41 | 3.40E-03 | 4.26E-02 |
| ENSMUSG00000026005 | Rpe | 1281.23 | 1385.73 | 1195.46 | 1526.61 | 1694.65 | 1893.09 | 0.41 | 2.59E-03 | 3.57E-02 |
| ENSMUSG00000017776 | Crk | 4274.64 | 4261.14 | 3842.02 | 4779.00 | 5093.71 | 6516.65 | 0.41 | 3.31E-03 | 4.20E-02 |
| ENSMUSG00000027761 | Aadac | 17634.35 | 17460.38 | 16977.88 | 21359.61 | 21601.68 | 25990.41 | 0.41 | 5.61E-04 | 1.25E-02 |
| ENSMUSG00000061731 | Ext1 | 2543.40 | 2697.20 | 2564.83 | 3081.21 | 3369.77 | 3875.23 | 0.40 | 1.05E-03 | 1.92E-02 |
| ENSMUSG00000006705 | Pknox1 | 743.32 | 733.42 | 723.17 | 965.70 | 958.73 | 978.65 | 0.40 | 1.48E-03 | 2.45E-02 |
| ENSMUSG00000031555 | Adam9 | 4758.54 | 5528.05 | 4258.25 | 5811.45 | 6500.74 | 6875.41 | 0.40 | 2.74E-03 | 3.70E-02 |
| ENSMUSG00000066440 | Zfyve26 | 2166.44 | 2356.76 | 1986.11 | 2793.76 | 2893.95 | 2890.58 | 0.40 | 1.54E-03 | 2.52E-02 |
| ENSMUSG00000079057 | Cyp4v3 | 22231.95 | 23365.43 | 21467.05 | 29717.21 | 31210.31 | 27412.58 | 0.40 | 1.90E-03 | 2.87E-02 |
| ENSMUSG00000022200 | Golph3 | 2438.57 | 2500.71 | 2160.96 | 2752.85 | 3064.40 | 3520.76 | 0.40 | 2.60E-03 | 3.58E-02 |
| ENSMUSG00000032633 | Flcn | 1757.72 | 1661.04 | 1540.42 | 2439.56 | 2022.22 | 2062.62 | 0.40 | 2.09E-03 | 3.10E-02 |
| ENSMUSG00000014226 | Cacybp | 1800.07 | 1530.81 | 1707.67 | 1983.09 | 2416.36 | 2203.90 | 0.39 | 2.72E-03 | 3.68E-02 |
| ENSMUSG00000020152 | Actr2 | 6630.62 | 6463.68 | 6266.21 | 6942.95 | 8796.38 | 9555.36 | 0.39 | 3.74E-03 | 4.59E-02 |
| ENSMUSG00000008730 | Hipk1 | 3457.20 | 3364.36 | 2862.27 | 3901.57 | 4129.65 | 4619.28 | 0.39 | 3.54E-03 | 4.38E-02 |
| ENSMUSG00000023068 | Nus1 | 4072.40 | 3559.71 | 3675.72 | 4432.34 | 4772.36 | 5520.87 | 0.38 | 2.22E-03 | 3.22E-02 |
| ENSMUSG00000058446 | Znrf2 | 5445.75 | 4638.13 | 4729.59 | 6181.80 | 6016.05 | 7054.36 | 0.38 | 2.76E-03 | 3.72E-02 |
| ENSMUSG00000021111 | Papola | 4895.14 | 5084.81 | 4886.39 | 5724.25 | 6518.50 | 7054.36 | 0.38 | 1.51E-03 | 2.48E-02 |
| ENSMUSG00000029063 | Nadk | 15195.78 | 13899.53 | 12628.40 | 18363.45 | 16595.85 | 19148.39 | 0.37 | 1.80E-03 | 2.78E-02 |
| ENSMUSG00000022770 | Dlg1 | 2218.32 | 2369.33 | 2062.13 | 2736.70 | 2924.14 | 2959.08 | 0.37 | 2.99E-03 | 3.93E-02 |
| ENSMUSG00000031645 | F11 | 3048.47 | 3177.00 | 2812.86 | 3730.40 | 4052.42 | 3924.03 | 0.37 | 4.19E-03 | 4.98E-02 |
| ENSMUSG00000041891 | Lman1 | 14028.91 | 15008.80 | 11524.17 | 16849.76 | 16907.44 | 18777.65 | 0.37 | 4.13E-03 | 4.93E-02 |
| ENSMUSG00000020883 | Fbxl20 | 1898.55 | 1903.23 | 1830.26 | 2559.06 | 2434.12 | 2290.38 | 0.37 | 2.76E-03 | 3.72E-02 |
| ENSMUSG00000033610 | Pank1 | 12574.03 | 12109.40 | 10802.90 | 14781.63 | 14619.79 | 16350.28 | 0.37 | 2.08E-03 | 3.09E-02 |
| ENSMUSG00000003810 | Mast2 | 1149.93 | 1121.83 | 1055.77 | 1346.82 | 1487.81 | 1438.44 | 0.36 | 3.27E-03 | 4.17E-02 |
| ENSMUSG00000032330 | Cox7a2 | 5535.75 | 6136.95 | 6143.62 | 7569.53 | 7252.64 | 7816.39 | 0.35 | 1.15E-03 | 2.05E-02 |
| ENSMUSG00000061947 | Serpina10 | 9003.54 | 10545.46 | 9172.20 | 11544.31 | 12075.60 | 12316.65 | 0.32 | 3.47E-03 | 4.32E-02 |
| ENSMUSG00000025980 | Hspd1 | 24992.41 | 22577.17 | 24664.78 | 29171.37 | 30614.66 | 29822.83 | 0.31 | 2.41E-03 | 3.37E-02 |

**Table S3. Down-regulated gene list in *Gprc6a^Liver-cko^* mice.**

The complete list of down-regulated genes in livers from *Gprc6a^Liver-cko^* mice compared to controls.

| **Ensembl Gene ID** | **Gene name** | **Wild type (WT)** | | | **Gprc6a-liver-cko** | | | **Gprc6a-liver-cko-vs-WT-log2FoldChange** | **Gprc6a-liver-cko-vs-WT-pvalue** | **Gprc6a-liver-cko-vs-WT-padj** |
| --- | --- | --- | --- | --- | --- | --- | --- | --- | --- | --- |
|  |  | **replicate**  **1** | **replicate**  **2** | **Replicate**  **3** | **replicate 1** | **replicate**  **2** | **Replicate**  **3** |  |  |  |
| ENSMUSG00000079845 | Xlr4a | 8.47 | 31.99 | 186.26 | 0.00 | 0.00 | 0.00 | -8.78 | 1.14E-09 | 2.82E-07 |
| ENSMUSG00000059751 | Rps3a3 | 2289.27 | 2257.37 | 2214.18 | 40.91 | 19.53 | 13.70 | -6.52 | 3.94E-35 | 7.06E-32 |
| ENSMUSG00000094497 | Gm8210 | 196.95 | 148.51 | 158.70 | 10.77 | 4.44 | 1.71 | -4.96 | 5.91E-21 | 7.07E-18 |
| ENSMUSG00000058126 | Tpm3-rs7 | 1012.28 | 975.61 | 1011.11 | 64.60 | 51.49 | 66.78 | -4.04 | 3.61E-45 | 8.64E-42 |
| ENSMUSG00000059058 | Tma7-ps | 587.67 | 519.79 | 640.50 | 51.68 | 32.85 | 32.54 | -3.91 | 5.46E-42 | 1.12E-38 |
| ENSMUSG00000082039 | Gm15653 | 78.36 | 57.12 | 69.37 | 8.61 | 9.76 | 0.00 | -3.50 | 4.61E-06 | 3.15E-04 |
| ENSMUSG00000062611 | Rps3a2 | 654.38 | 1000.74 | 859.06 | 75.36 | 65.69 | 83.91 | -3.48 | 2.08E-52 | 5.97E-49 |
| ENSMUSG00000072572 | Slc39a2 | 40.24 | 203.35 | 210.96 | 29.07 | 2.66 | 11.99 | -3.39 | 6.20E-04 | 1.34E-02 |
| ENSMUSG00000097969 | #N/A | 57.18 | 18.28 | 27.56 | 6.46 | 0.00 | 5.14 | -3.18 | 5.08E-05 | 2.09E-03 |
| ENSMUSG00000103898 | Gm30238 | 27.53 | 15.99 | 38.01 | 6.46 | 0.00 | 3.42 | -3.08 | 5.50E-04 | 1.24E-02 |
| ENSMUSG00000083833 | Gm13841 | 121.77 | 287.88 | 216.67 | 32.30 | 15.98 | 30.82 | -2.99 | 7.75E-15 | 4.83E-12 |
| ENSMUSG00000076617 | Ighm | 1195.46 | 1368.59 | 2273.09 | 90.43 | 405.69 | 137.85 | -2.93 | 2.18E-05 | 1.07E-03 |
| ENSMUSG00000106705 | Gm2602 | 19.06 | 18.28 | 38.01 | 8.61 | 2.66 | 0.00 | -2.81 | 5.15E-04 | 1.19E-02 |
| ENSMUSG00000096964 | Gm6345 | 42.35 | 6.85 | 34.21 | 4.31 | 0.89 | 6.85 | -2.79 | 1.14E-03 | 2.03E-02 |
| ENSMUSG00000022132 | Cldn10 | 46.59 | 36.56 | 15.20 | 6.46 | 7.10 | 1.71 | -2.70 | 3.64E-04 | 9.09E-03 |
| ENSMUSG00000043753 | Dmrta1 | 224.48 | 197.63 | 135.89 | 55.98 | 23.08 | 10.27 | -2.66 | 1.88E-09 | 4.41E-07 |
| ENSMUSG00000085834 | Gm15622 | 55.06 | 27.42 | 58.92 | 15.07 | 4.44 | 3.42 | -2.65 | 1.32E-03 | 2.26E-02 |
| ENSMUSG00000103680 | Gm37072 | 58.24 | 10.28 | 44.66 | 14.00 | 1.78 | 3.42 | -2.60 | 8.60E-04 | 1.67E-02 |
| ENSMUSG00000078249 | Hmga1b | 60.36 | 13.71 | 13.30 | 4.31 | 7.10 | 3.42 | -2.56 | 1.81E-03 | 2.79E-02 |
| ENSMUSG00000028359 | Orm3 | 192.71 | 290.17 | 299.34 | 29.07 | 82.56 | 23.97 | -2.53 | 1.67E-07 | 1.96E-05 |
| ENSMUSG00000086962 | Gm12248 | 43.41 | 42.27 | 77.92 | 15.07 | 0.00 | 14.56 | -2.47 | 2.47E-03 | 3.44E-02 |
| ENSMUSG00000087231 | E230016M11Rik | 33.88 | 12.57 | 23.76 | 4.31 | 2.66 | 5.99 | -2.44 | 9.30E-04 | 1.76E-02 |
| ENSMUSG00000061126 | Cyp4f39 | 37.06 | 36.56 | 28.51 | 11.84 | 7.10 | 1.71 | -2.34 | 8.41E-05 | 3.04E-03 |
| ENSMUSG00000053702 | Nebl | 82.59 | 130.23 | 107.38 | 18.30 | 20.42 | 24.83 | -2.33 | 4.33E-09 | 8.81E-07 |
| ENSMUSG00000003379 | Cd79a | 31.77 | 37.70 | 78.87 | 10.77 | 10.65 | 8.56 | -2.31 | 2.73E-05 | 1.28E-03 |
| ENSMUSG00000092981 | Mir5125 | 105.89 | 47.98 | 99.78 | 30.14 | 5.33 | 17.12 | -2.29 | 4.91E-05 | 2.03E-03 |
| ENSMUSG00000041750 | Cd1d2 | 51.88 | 90.25 | 48.46 | 25.84 | 9.76 | 5.14 | -2.25 | 4.27E-05 | 1.81E-03 |
| ENSMUSG00000054545 | Ugt1a6a | 130.24 | 66.26 | 141.59 | 40.91 | 13.32 | 17.98 | -2.24 | 6.67E-06 | 4.27E-04 |
| ENSMUSG00000038246 | Fam50b | 42.35 | 36.56 | 26.61 | 10.77 | 10.65 | 1.71 | -2.21 | 1.12E-04 | 3.86E-03 |
| ENSMUSG00000106538 | Gm30301 | 54.00 | 11.42 | 38.01 | 17.23 | 3.55 | 2.57 | -2.18 | 3.88E-03 | 4.71E-02 |
| ENSMUSG00000020651 | Slc26a4 | 73.06 | 54.84 | 60.82 | 10.77 | 19.53 | 11.99 | -2.15 | 4.13E-07 | 4.35E-05 |
| ENSMUSG00000022408 | Fam83f | 405.55 | 180.50 | 141.59 | 49.52 | 71.02 | 44.52 | -2.14 | 8.54E-09 | 1.57E-06 |
| ENSMUSG00000072999 | Gm15401 | 25.41 | 23.99 | 21.86 | 9.69 | 5.33 | 1.71 | -2.12 | 4.11E-03 | 4.91E-02 |
| ENSMUSG00000026586 | Prrx1 | 59.30 | 29.70 | 58.92 | 17.23 | 7.10 | 10.27 | -2.12 | 1.05E-04 | 3.63E-03 |
| ENSMUSG00000074213 | Gm10642 | 8.47 | 27.42 | 29.46 | 8.61 | 3.55 | 3.42 | -2.11 | 5.67E-04 | 1.25E-02 |
| ENSMUSG00000087259 | 2610035D17Rik | 30.71 | 38.84 | 46.56 | 9.69 | 7.99 | 10.27 | -2.06 | 6.67E-05 | 2.55E-03 |
| ENSMUSG00000044734 | Serpinb1a | 986.86 | 528.93 | 789.69 | 214.24 | 254.77 | 94.18 | -2.03 | 7.08E-06 | 4.46E-04 |
| ENSMUSG00000074052 | BC048644 | 61.41 | 19.42 | 31.36 | 14.00 | 5.33 | 8.56 | -2.02 | 1.94E-03 | 2.92E-02 |
| ENSMUSG00000040998 | Npnt | 103.77 | 68.54 | 81.72 | 23.69 | 28.41 | 11.99 | -1.99 | 6.85E-07 | 6.69E-05 |
| ENSMUSG00000086938 | 4930481A15Rik | 289.07 | 310.73 | 244.22 | 49.52 | 90.55 | 71.92 | -1.98 | 5.81E-15 | 3.79E-12 |
| ENSMUSG00000104520 | Gm37336 | 55.06 | 50.27 | 58.92 | 19.38 | 23.08 | 0.00 | -1.96 | 1.86E-03 | 2.84E-02 |
| ENSMUSG00000026077 | Npas2 | 154.59 | 116.52 | 117.84 | 38.76 | 58.59 | 6.85 | -1.90 | 3.46E-05 | 1.55E-03 |
| ENSMUSG00000028008 | Asic5 | 1220.87 | 900.21 | 860.01 | 218.55 | 267.20 | 311.66 | -1.90 | 3.83E-22 | 5.00E-19 |
| ENSMUSG00000028238 | Atp6v0d2 | 122.83 | 100.53 | 129.24 | 23.69 | 33.73 | 38.53 | -1.87 | 2.46E-05 | 1.18E-03 |
| ENSMUSG00000019577 | Pdk4 | 656.50 | 379.28 | 547.37 | 111.97 | 186.42 | 136.99 | -1.86 | 4.67E-11 | 1.72E-08 |
| ENSMUSG00000090090 | Mup-ps16 | 96.36 | 25.13 | 79.82 | 30.14 | 8.88 | 17.12 | -1.86 | 1.09E-03 | 1.99E-02 |
| ENSMUSG00000043924 | Ncmap | 59.30 | 68.54 | 70.32 | 20.46 | 14.20 | 20.55 | -1.85 | 1.16E-05 | 6.49E-04 |
| ENSMUSG00000048489 | Depp1 | 8930.47 | 6965.19 | 8231.41 | 2997.24 | 820.25 | 2911.13 | -1.84 | 1.59E-04 | 4.94E-03 |
| ENSMUSG00000038508 | Gdf15 | 1238.87 | 567.77 | 847.66 | 376.81 | 223.70 | 147.27 | -1.83 | 2.79E-04 | 7.47E-03 |
| ENSMUSG00000026610 | Esrrg | 83.65 | 113.10 | 110.23 | 15.07 | 30.18 | 41.10 | -1.81 | 3.59E-07 | 3.90E-05 |
| ENSMUSG00000055818 | A230083G16Rik | 36.00 | 36.56 | 44.66 | 19.38 | 7.99 | 6.85 | -1.80 | 7.10E-04 | 1.48E-02 |
| ENSMUSG00000057836 | Xlr3a | 442.61 | 249.04 | 445.69 | 135.65 | 31.96 | 167.82 | -1.76 | 2.10E-03 | 3.11E-02 |
| ENSMUSG00000067279 | Ppp1r3c | 5182.09 | 3958.40 | 5035.59 | 1221.93 | 1097.22 | 1887.10 | -1.75 | 7.08E-21 | 7.82E-18 |
| ENSMUSG00000055116 | Arntl | 803.68 | 694.58 | 639.55 | 239.00 | 319.58 | 89.05 | -1.72 | 7.09E-09 | 1.34E-06 |
| ENSMUSG00000040592 | Cd79b | 31.77 | 36.56 | 40.86 | 8.61 | 7.10 | 17.12 | -1.72 | 8.43E-04 | 1.65E-02 |
| ENSMUSG00000076612 | Ighg2c | 152.48 | 87.96 | 80.77 | 27.99 | 41.72 | 29.97 | -1.68 | 6.27E-05 | 2.43E-03 |
| ENSMUSG00000069917 | Hba-a2 | 1463.35 | 527.79 | 905.63 | 299.29 | 338.22 | 309.09 | -1.61 | 1.60E-09 | 3.83E-07 |
| ENSMUSG00000022096 | Hr | 150.36 | 83.39 | 198.61 | 49.52 | 76.34 | 18.84 | -1.58 | 1.80E-04 | 5.34E-03 |
| ENSMUSG00000063704 | Mapk15 | 3009.30 | 1632.48 | 2680.77 | 1259.61 | 727.93 | 517.15 | -1.55 | 4.01E-05 | 1.74E-03 |
| ENSMUSG00000025726 | Slc28a1 | 158.83 | 94.82 | 92.18 | 60.29 | 38.17 | 23.12 | -1.52 | 3.43E-04 | 8.67E-03 |
| ENSMUSG00000076609 | Igkc | 591.91 | 857.94 | 948.39 | 188.40 | 452.74 | 201.21 | -1.51 | 2.26E-03 | 3.25E-02 |
| ENSMUSG00000095385 | D630033O11Rik | 110.12 | 103.96 | 103.58 | 43.06 | 52.38 | 17.98 | -1.49 | 2.89E-04 | 7.60E-03 |
| ENSMUSG00000043993 | 2900052L18Rik | 142.95 | 91.39 | 129.24 | 45.22 | 33.73 | 52.23 | -1.47 | 2.11E-06 | 1.65E-04 |
| ENSMUSG00000045776 | Lrtm1 | 620.50 | 292.45 | 445.69 | 150.72 | 195.30 | 147.27 | -1.46 | 1.87E-06 | 1.49E-04 |
| ENSMUSG00000052563 | D930048N14Rik | 277.42 | 151.94 | 191.96 | 130.27 | 49.71 | 47.95 | -1.46 | 1.74E-04 | 5.26E-03 |
| ENSMUSG00000042248 | Cyp2c37 | 13363.95 | 16465.36 | 11909.98 | 8190.72 | 5299.66 | 1849.42 | -1.44 | 2.90E-03 | 3.85E-02 |
| ENSMUSG00000029822 | Osbpl3 | 280.60 | 82.25 | 238.52 | 72.13 | 69.24 | 80.48 | -1.44 | 1.65E-05 | 8.60E-04 |
| ENSMUSG00000052133 | Sema5b | 380.13 | 234.19 | 590.13 | 159.34 | 154.46 | 132.71 | -1.43 | 1.37E-07 | 1.69E-05 |
| ENSMUSG00000038751 | Ptk6 | 52.94 | 63.97 | 90.28 | 27.99 | 31.96 | 17.12 | -1.43 | 1.69E-03 | 2.68E-02 |
| ENSMUSG00000091867 | Cyp2a22 | 184.24 | 167.93 | 142.54 | 59.21 | 53.26 | 72.78 | -1.41 | 6.73E-08 | 9.57E-06 |
| ENSMUSG00000089665 | Fcor | 63.53 | 45.70 | 45.61 | 23.69 | 21.31 | 13.70 | -1.41 | 6.13E-04 | 1.32E-02 |
| ENSMUSG00000027356 | Fermt1 | 67.77 | 49.12 | 42.76 | 25.84 | 8.88 | 25.69 | -1.41 | 3.08E-03 | 4.01E-02 |
| ENSMUSG00000028919 | Arhgef19 | 5801.53 | 5452.66 | 5346.33 | 2519.23 | 2279.65 | 1534.34 | -1.39 | 2.71E-16 | 2.43E-13 |
| ENSMUSG00000028457 | Atp8b5 | 55.06 | 47.98 | 91.23 | 31.22 | 17.75 | 25.69 | -1.39 | 8.21E-04 | 1.63E-02 |
| ENSMUSG00000025202 | Scd3 | 203.30 | 57.12 | 60.82 | 43.06 | 24.86 | 55.65 | -1.38 | 3.11E-03 | 4.04E-02 |
| ENSMUSG00000044916 | 1700029I15Rik | 57.18 | 47.98 | 90.28 | 19.38 | 23.08 | 33.39 | -1.36 | 7.45E-04 | 1.53E-02 |
| ENSMUSG00000026840 | Lamc3 | 167.30 | 73.11 | 132.09 | 32.30 | 56.81 | 55.65 | -1.36 | 5.58E-04 | 1.24E-02 |
| ENSMUSG00000021200 | Asb2 | 153.54 | 171.36 | 135.89 | 48.45 | 55.93 | 76.20 | -1.35 | 7.78E-05 | 2.89E-03 |
| ENSMUSG00000001095 | Slc13a2 | 150.36 | 146.23 | 84.58 | 64.60 | 34.62 | 51.37 | -1.34 | 6.14E-05 | 2.40E-03 |
| ENSMUSG00000037583 | Nr0b2 | 7409.94 | 4774.07 | 4818.92 | 3283.61 | 1270.32 | 2177.35 | -1.34 | 2.11E-04 | 6.04E-03 |
| ENSMUSG00000037827 | Gm5884 | 85.77 | 114.24 | 105.48 | 36.60 | 33.73 | 50.52 | -1.33 | 9.21E-05 | 3.28E-03 |
| ENSMUSG00000044317 | Gpr4 | 114.36 | 91.39 | 83.63 | 49.52 | 31.96 | 34.25 | -1.33 | 8.54E-05 | 3.08E-03 |
| ENSMUSG00000039485 | Tspyl4 | 216.01 | 207.92 | 210.96 | 109.81 | 102.98 | 41.95 | -1.32 | 1.11E-03 | 2.00E-02 |
| ENSMUSG00000068101 | Cenpm | 213.89 | 166.79 | 198.61 | 69.98 | 100.31 | 61.65 | -1.32 | 2.36E-05 | 1.14E-03 |
| ENSMUSG00000040312 | Cchcr1 | 253.07 | 173.64 | 225.22 | 116.27 | 93.21 | 55.65 | -1.31 | 3.77E-06 | 2.66E-04 |
| ENSMUSG00000012187 | Mogat1 | 98.47 | 29.70 | 111.18 | 38.76 | 39.95 | 18.84 | -1.30 | 3.14E-03 | 4.05E-02 |
| ENSMUSG00000022941 | Ripply3 | 93.18 | 105.10 | 157.75 | 43.06 | 39.06 | 66.78 | -1.26 | 2.66E-04 | 7.22E-03 |
| ENSMUSG00000033083 | Tbc1d4 | 494.49 | 450.10 | 421.93 | 187.33 | 164.23 | 220.90 | -1.25 | 3.07E-09 | 6.67E-07 |
| ENSMUSG00000082248 | Gm13161 | 46.59 | 38.84 | 53.22 | 20.46 | 25.74 | 12.84 | -1.24 | 3.19E-03 | 4.09E-02 |
| ENSMUSG00000027478 | Dnmt3b | 122.83 | 97.10 | 141.59 | 45.22 | 52.38 | 56.51 | -1.23 | 3.07E-05 | 1.39E-03 |
| ENSMUSG00000029009 | Mthfr | 1050.39 | 765.41 | 718.42 | 396.19 | 484.69 | 202.07 | -1.23 | 8.85E-06 | 5.36E-04 |
| ENSMUSG00000023017 | Asic1 | 231.89 | 188.50 | 243.27 | 78.59 | 97.65 | 107.88 | -1.22 | 9.47E-08 | 1.23E-05 |
| ENSMUSG00000067225 | Cyp2c54 | 11855.06 | 15771.92 | 9239.67 | 4909.27 | 5788.80 | 5205.79 | -1.21 | 4.29E-10 | 1.21E-07 |
| ENSMUSG00000081208 | Gm11400 | 116.48 | 67.40 | 153.00 | 79.67 | 36.40 | 30.82 | -1.21 | 2.67E-03 | 3.65E-02 |
| ENSMUSG00000096105 | Bhmt-ps1 | 396.02 | 306.16 | 287.94 | 226.08 | 126.94 | 80.48 | -1.20 | 2.21E-04 | 6.22E-03 |
| ENSMUSG00000036957 | Lrfn3 | 1408.29 | 1280.63 | 1412.13 | 792.37 | 658.69 | 344.20 | -1.19 | 1.39E-07 | 1.70E-05 |
| ENSMUSG00000086771 | 1700080G11Rik | 61.41 | 119.95 | 125.44 | 35.53 | 50.60 | 47.95 | -1.19 | 1.01E-03 | 1.87E-02 |
| ENSMUSG00000026489 | Coq8a | 41075.52 | 36898.25 | 36605.17 | 19820.08 | 19083.23 | 11315.74 | -1.19 | 2.14E-11 | 8.28E-09 |
| ENSMUSG00000041827 | Oasl1 | 1645.48 | 1028.16 | 1166.96 | 473.70 | 651.58 | 557.40 | -1.19 | 2.51E-07 | 2.93E-05 |
| ENSMUSG00000044186 | Nkx2-6 | 165.18 | 90.25 | 93.13 | 68.90 | 21.31 | 63.36 | -1.19 | 1.48E-03 | 2.45E-02 |
| ENSMUSG00000020037 | Rfx4 | 314.48 | 364.42 | 221.42 | 190.56 | 159.79 | 46.24 | -1.19 | 9.02E-04 | 1.73E-02 |
| ENSMUSG00000023087 | Noct | 5797.29 | 4294.27 | 5376.74 | 2609.66 | 2219.29 | 1979.57 | -1.18 | 2.48E-12 | 1.32E-09 |
| ENSMUSG00000032065 | Tex12 | 146.12 | 95.96 | 95.98 | 48.45 | 62.14 | 39.39 | -1.17 | 1.96E-03 | 2.93E-02 |
| ENSMUSG00000029003 | Mad2l2 | 1271.70 | 1595.93 | 1261.99 | 735.31 | 710.17 | 393.86 | -1.17 | 7.84E-08 | 1.09E-05 |
| ENSMUSG00000028755 | Cda | 488.14 | 894.50 | 637.64 | 344.51 | 233.47 | 321.94 | -1.17 | 1.60E-06 | 1.35E-04 |
| ENSMUSG00000006711 | D130043K22Rik | 462.72 | 460.39 | 348.76 | 179.79 | 182.87 | 205.49 | -1.16 | 2.75E-10 | 8.96E-08 |
| ENSMUSG00000022129 | Dct | 379.07 | 169.07 | 338.30 | 127.04 | 107.41 | 163.54 | -1.16 | 1.66E-04 | 5.07E-03 |
| ENSMUSG00000008153 | Clstn3 | 3517.55 | 1726.16 | 3253.79 | 1661.18 | 1201.08 | 962.39 | -1.15 | 7.75E-07 | 7.52E-05 |
| ENSMUSG00000005125 | Ndrg1 | 933.92 | 771.12 | 549.27 | 275.61 | 344.43 | 394.72 | -1.15 | 1.16E-06 | 1.03E-04 |
| ENSMUSG00000045193 | Cirbp | 1262.17 | 1051.00 | 1214.47 | 643.80 | 435.87 | 516.30 | -1.15 | 5.13E-12 | 2.37E-09 |
| ENSMUSG00000044854 | 1700056E22Rik | 118.59 | 70.83 | 121.64 | 53.83 | 51.49 | 35.96 | -1.15 | 4.44E-04 | 1.06E-02 |
| ENSMUSG00000022528 | Hes1 | 757.09 | 815.67 | 968.35 | 512.46 | 274.30 | 365.60 | -1.14 | 8.98E-08 | 1.19E-05 |
| ENSMUSG00000025902 | Sox17 | 114.36 | 89.11 | 134.94 | 83.97 | 29.29 | 42.81 | -1.13 | 2.25E-03 | 3.25E-02 |
| ENSMUSG00000022215 | Fitm1 | 6300.25 | 3493.45 | 6650.13 | 3305.14 | 1838.46 | 2434.22 | -1.12 | 2.59E-06 | 1.91E-04 |
| ENSMUSG00000020142 | Slc1a4 | 478.61 | 427.26 | 418.13 | 251.92 | 264.54 | 97.61 | -1.11 | 4.86E-05 | 2.03E-03 |
| ENSMUSG00000026442 | Nfasc | 185.30 | 211.34 | 241.37 | 110.89 | 127.83 | 58.22 | -1.11 | 2.33E-04 | 6.51E-03 |
| ENSMUSG00000086332 | 4930480G23Rik | 159.89 | 118.81 | 151.10 | 41.99 | 58.59 | 98.46 | -1.10 | 5.15E-04 | 1.19E-02 |
| ENSMUSG00000030762 | Aqp8 | 14573.17 | 7008.60 | 9788.94 | 5615.51 | 5359.14 | 3627.78 | -1.10 | 4.86E-07 | 4.91E-05 |
| ENSMUSG00000105095 | 8430422M14Rik | 85.77 | 67.40 | 110.23 | 58.14 | 37.28 | 28.26 | -1.10 | 3.15E-03 | 4.06E-02 |
| ENSMUSG00000020083 | Fam241b | 192.71 | 100.53 | 191.96 | 68.90 | 97.65 | 59.94 | -1.10 | 6.22E-04 | 1.34E-02 |
| ENSMUSG00000029022 | Miip | 526.26 | 555.20 | 658.55 | 310.06 | 230.81 | 276.56 | -1.09 | 8.85E-10 | 2.27E-07 |
| ENSMUSG00000002944 | Cd36 | 2835.64 | 1435.99 | 1881.57 | 732.08 | 854.87 | 1304.02 | -1.09 | 2.11E-06 | 1.65E-04 |
| ENSMUSG00000020926 | Adam11 | 1833.96 | 2252.81 | 1684.86 | 1259.61 | 719.05 | 764.60 | -1.07 | 1.23E-05 | 6.83E-04 |
| ENSMUSG00000023018 | Smarcd1 | 714.73 | 392.98 | 610.09 | 283.14 | 292.95 | 245.73 | -1.06 | 3.26E-06 | 2.34E-04 |
| ENSMUSG00000052957 | Gas1 | 4278.88 | 2303.07 | 2260.74 | 1224.09 | 1244.58 | 1789.49 | -1.05 | 8.37E-06 | 5.18E-04 |
| ENSMUSG00000029001 | Fbxo44 | 163.07 | 149.65 | 167.25 | 99.05 | 71.02 | 63.36 | -1.05 | 2.92E-05 | 1.34E-03 |
| ENSMUSG00000054827 | Cyp2c50 | 29974.37 | 36313.35 | 26786.77 | 16131.68 | 19403.69 | 9615.30 | -1.04 | 4.61E-07 | 4.69E-05 |
| ENSMUSG00000067203 | H2-K2 | 368.49 | 189.64 | 309.79 | 177.64 | 104.75 | 140.42 | -1.04 | 9.68E-04 | 1.82E-02 |
| ENSMUSG00000030806 | Stx1b | 423.55 | 305.02 | 580.63 | 220.70 | 230.81 | 189.22 | -1.03 | 5.40E-06 | 3.54E-04 |
| ENSMUSG00000008348 | Ubc | 14385.75 | 11066.39 | 12281.55 | 6866.51 | 6529.15 | 5097.05 | -1.03 | 3.47E-10 | 1.04E-07 |
| ENSMUSG00000070690 | 5830473C10Rik | 2717.05 | 2450.44 | 2456.50 | 1141.19 | 907.25 | 1686.74 | -1.03 | 4.07E-08 | 5.96E-06 |
| ENSMUSG00000100017 | 2410022M11Rik | 470.14 | 347.29 | 442.84 | 251.92 | 139.37 | 227.75 | -1.03 | 3.94E-05 | 1.71E-03 |
| ENSMUSG00000002083 | Bbc3 | 2269.15 | 1697.60 | 1856.87 | 1395.27 | 743.91 | 724.36 | -1.03 | 5.50E-06 | 3.57E-04 |
| ENSMUSG00000078452 | Raet1d | 2010.79 | 1667.90 | 2387.13 | 579.21 | 1134.50 | 1264.63 | -1.03 | 1.03E-05 | 5.91E-04 |
| ENSMUSG00000078496 | Zfp982 | 214.95 | 265.04 | 195.76 | 124.88 | 123.39 | 84.77 | -1.02 | 1.60E-05 | 8.32E-04 |
| ENSMUSG00000085468 | Gm15343 | 139.77 | 150.80 | 112.13 | 77.51 | 54.15 | 67.64 | -1.02 | 3.93E-04 | 9.71E-03 |
| ENSMUSG00000038884 | A230050P20Rik | 1983.26 | 1321.75 | 1628.80 | 919.41 | 794.51 | 732.06 | -1.01 | 4.57E-08 | 6.63E-06 |
| ENSMUSG00000004098 | Col5a3 | 1689.95 | 771.12 | 1480.55 | 814.98 | 576.13 | 565.96 | -1.01 | 5.91E-05 | 2.34E-03 |
| ENSMUSG00000022010 | Tsc22d1 | 6968.40 | 4616.42 | 5004.23 | 2636.58 | 2995.15 | 2600.33 | -1.01 | 3.40E-10 | 1.04E-07 |
| ENSMUSG00000039620 | Trmt9b | 369.54 | 293.60 | 462.79 | 145.34 | 146.47 | 271.42 | -1.00 | 1.36E-04 | 4.44E-03 |
| ENSMUSG00000044378 | Slc15a5 | 623.67 | 366.71 | 542.62 | 283.14 | 253.89 | 232.03 | -1.00 | 1.70E-06 | 1.40E-04 |
| ENSMUSG00000015357 | Clpx | 14004.56 | 12249.91 | 11837.76 | 6857.90 | 7568.67 | 4655.24 | -1.00 | 3.39E-09 | 7.15E-07 |
| ENSMUSG00000027762 | Sucnr1 | 2676.81 | 2485.85 | 2189.47 | 1533.07 | 1234.81 | 918.72 | -1.00 | 9.46E-09 | 1.70E-06 |
| ENSMUSG00000102375 | A930036K24Rik | 111.18 | 195.35 | 219.52 | 114.12 | 95.87 | 54.80 | -1.00 | 3.28E-03 | 4.17E-02 |
| ENSMUSG00000034917 | Tjp3 | 4002.51 | 3246.69 | 2843.27 | 1905.57 | 2095.01 | 1083.11 | -0.99 | 1.49E-06 | 1.26E-04 |
| ENSMUSG00000094156 | Sult2a7 | 148.24 | 205.63 | 163.45 | 96.89 | 75.46 | 89.05 | -0.99 | 1.03E-03 | 1.90E-02 |
| ENSMUSG00000021379 | Id4 | 121.77 | 93.68 | 173.90 | 64.60 | 67.47 | 65.07 | -0.99 | 1.19E-03 | 2.09E-02 |
| ENSMUSG00000003053 | Cyp2c29 | 62730.39 | 65925.39 | 46105.22 | 25436.67 | 29550.29 | 33298.20 | -0.99 | 7.04E-11 | 2.52E-08 |
| ENSMUSG00000034035 | Ccdc17 | 122.83 | 133.66 | 149.20 | 94.74 | 61.25 | 51.37 | -0.98 | 1.25E-03 | 2.16E-02 |
| ENSMUSG00000027459 | Fam110a | 400.25 | 247.90 | 347.81 | 163.64 | 171.33 | 172.10 | -0.97 | 1.58E-05 | 8.32E-04 |
| ENSMUSG00000062590 | Armc9 | 386.49 | 491.23 | 380.12 | 232.54 | 194.41 | 215.77 | -0.97 | 2.63E-07 | 3.02E-05 |
| ENSMUSG00000055546 | Timd4 | 328.25 | 308.45 | 385.82 | 135.65 | 218.38 | 170.39 | -0.96 | 5.43E-04 | 1.23E-02 |
| ENSMUSG00000025498 | Irf7 | 3722.97 | 2431.02 | 3957.01 | 1225.16 | 1897.94 | 2099.44 | -0.95 | 9.60E-06 | 5.74E-04 |
| ENSMUSG00000058486 | Wdr91 | 1548.06 | 1242.93 | 1209.72 | 853.74 | 727.04 | 488.90 | -0.95 | 5.25E-07 | 5.26E-05 |
| ENSMUSG00000024697 | Gna14 | 349.43 | 498.08 | 515.06 | 348.82 | 205.95 | 151.55 | -0.95 | 1.48E-03 | 2.45E-02 |
| ENSMUSG00000046338 | Gpat2 | 168.36 | 159.94 | 128.29 | 82.90 | 112.74 | 41.10 | -0.95 | 2.67E-03 | 3.66E-02 |
| ENSMUSG00000020381 | Mrnip | 460.61 | 371.28 | 363.96 | 261.61 | 237.91 | 124.15 | -0.94 | 1.37E-04 | 4.46E-03 |
| ENSMUSG00000015217 | Hmgb3 | 676.62 | 654.59 | 649.05 | 331.59 | 374.62 | 331.36 | -0.93 | 1.53E-09 | 3.72E-07 |
| ENSMUSG00000021281 | Tnfaip2 | 1912.31 | 1478.26 | 1564.18 | 772.99 | 820.25 | 1012.90 | -0.93 | 1.49E-08 | 2.49E-06 |
| ENSMUSG00000042115 | Klhdc8a | 247.77 | 290.17 | 233.77 | 133.50 | 158.01 | 114.73 | -0.92 | 6.34E-05 | 2.45E-03 |
| ENSMUSG00000009566 | Fpgs | 13501.60 | 11331.43 | 11093.68 | 7541.54 | 6879.80 | 4711.75 | -0.91 | 3.41E-08 | 5.26E-06 |
| ENSMUSG00000049600 | Zbtb45 | 355.78 | 244.47 | 417.18 | 274.53 | 124.28 | 146.41 | -0.91 | 1.59E-03 | 2.58E-02 |
| ENSMUSG00000043068 | Fam89a | 825.92 | 1108.12 | 1115.64 | 361.74 | 545.06 | 719.22 | -0.91 | 5.20E-05 | 2.13E-03 |
| ENSMUSG00000041378 | Cldn5 | 864.03 | 852.23 | 994.00 | 572.75 | 459.84 | 416.12 | -0.91 | 2.45E-06 | 1.84E-04 |
| ENSMUSG00000046160 | Olig1 | 938.16 | 431.83 | 822.95 | 368.19 | 355.09 | 451.23 | -0.90 | 5.84E-05 | 2.32E-03 |
| ENSMUSG00000022474 | Pmm1 | 459.55 | 310.73 | 363.96 | 228.24 | 227.26 | 154.12 | -0.90 | 1.68E-05 | 8.71E-04 |
| ENSMUSG00000061959 | Ces1e | 16655.96 | 16123.78 | 16372.55 | 9349.14 | 6845.18 | 10284.00 | -0.89 | 6.73E-10 | 1.79E-07 |
| ENSMUSG00000030814 | Bcl7c | 2024.55 | 1087.56 | 1376.97 | 1080.90 | 767.87 | 574.52 | -0.89 | 2.83E-04 | 7.51E-03 |
| ENSMUSG00000045377 | Tmem88 | 180.01 | 139.37 | 184.36 | 93.66 | 94.10 | 85.62 | -0.88 | 7.05E-04 | 1.47E-02 |
| ENSMUSG00000032363 | Adamts7 | 1162.63 | 1077.28 | 510.31 | 550.14 | 502.45 | 440.09 | -0.88 | 2.55E-04 | 7.00E-03 |
| ENSMUSG00000032420 | Nt5e | 787.80 | 801.96 | 546.42 | 282.07 | 301.82 | 580.51 | -0.87 | 8.01E-04 | 1.61E-02 |
| ENSMUSG00000067199 | Frat1 | 634.26 | 584.91 | 511.26 | 411.26 | 167.78 | 367.32 | -0.87 | 5.78E-04 | 1.26E-02 |
| ENSMUSG00000003378 | Grik5 | 888.39 | 568.91 | 1049.12 | 589.97 | 370.18 | 413.55 | -0.87 | 1.43E-04 | 4.59E-03 |
| ENSMUSG00000052632 | Asap2 | 552.73 | 538.07 | 459.94 | 284.22 | 343.55 | 220.90 | -0.87 | 4.53E-06 | 3.11E-04 |
| ENSMUSG00000079434 | Neu2 | 115.42 | 132.52 | 114.03 | 55.98 | 81.67 | 59.94 | -0.87 | 2.16E-03 | 3.17E-02 |
| ENSMUSG00000056749 | Nfil3 | 2638.69 | 2420.74 | 3055.18 | 1633.19 | 1413.24 | 1405.91 | -0.87 | 5.47E-06 | 3.57E-04 |
| ENSMUSG00000078201 | Tmem203 | 565.43 | 384.99 | 469.44 | 324.05 | 248.56 | 208.06 | -0.87 | 5.62E-05 | 2.27E-03 |
| ENSMUSG00000028716 | Pdzk1ip1 | 226.60 | 114.24 | 174.85 | 79.67 | 117.18 | 87.33 | -0.86 | 3.84E-03 | 4.68E-02 |
| ENSMUSG00000073940 | Hbb-bt | 1375.47 | 749.41 | 1124.19 | 458.63 | 657.80 | 675.55 | -0.86 | 9.25E-05 | 3.28E-03 |
| ENSMUSG00000020123 | Avpr1a | 1551.24 | 2673.21 | 1592.69 | 1345.74 | 1273.87 | 597.64 | -0.85 | 7.45E-04 | 1.53E-02 |
| ENSMUSG00000020175 | Rab36 | 411.90 | 450.10 | 450.44 | 335.90 | 240.57 | 152.41 | -0.85 | 2.72E-04 | 7.36E-03 |
| ENSMUSG00000023031 | Cela1 | 3096.12 | 3588.27 | 3329.82 | 2476.16 | 1925.46 | 1148.18 | -0.85 | 2.97E-04 | 7.71E-03 |
| ENSMUSG00000060470 | Adgrg3 | 190.60 | 159.94 | 136.84 | 103.35 | 85.22 | 82.20 | -0.85 | 1.02E-03 | 1.89E-02 |
| ENSMUSG00000037344 | Slc12a9 | 731.68 | 462.67 | 772.59 | 502.77 | 275.19 | 316.80 | -0.85 | 7.61E-04 | 1.55E-02 |
| ENSMUSG00000027956 | Tmem144 | 432.02 | 349.57 | 307.89 | 198.09 | 225.48 | 181.52 | -0.85 | 7.96E-05 | 2.92E-03 |
| ENSMUSG00000042363 | Lgalsl | 1294.99 | 1001.88 | 905.63 | 537.22 | 831.79 | 412.70 | -0.85 | 1.59E-04 | 4.94E-03 |
| ENSMUSG00000009647 | Mcu | 1846.66 | 1530.81 | 1642.10 | 1031.38 | 1025.31 | 738.91 | -0.85 | 7.33E-08 | 1.03E-05 |
| ENSMUSG00000049907 | Rasl11b | 177.89 | 187.35 | 216.67 | 79.67 | 106.53 | 136.99 | -0.84 | 1.50E-03 | 2.47E-02 |
| ENSMUSG00000022948 | Setd4 | 470.14 | 375.85 | 428.58 | 260.54 | 233.47 | 218.33 | -0.84 | 5.51E-05 | 2.24E-03 |
| ENSMUSG00000020538 | Srebf1 | 47008.35 | 39087.08 | 36775.27 | 25123.38 | 18174.21 | 25642.78 | -0.83 | 8.92E-07 | 8.36E-05 |
| ENSMUSG00000067144 | Slc22a7 | 3235.89 | 6138.09 | 5880.39 | 4071.68 | 2361.32 | 2131.12 | -0.83 | 5.17E-04 | 1.19E-02 |
| ENSMUSG00000055652 | Klhl25 | 1781.01 | 1699.89 | 1434.94 | 826.82 | 756.33 | 1174.73 | -0.83 | 2.19E-06 | 1.70E-04 |
| ENSMUSG00000038181 | Chpf2 | 1163.69 | 1192.66 | 1108.04 | 601.82 | 561.92 | 781.72 | -0.83 | 4.86E-06 | 3.29E-04 |
| ENSMUSG00000032349 | Elovl5 | 47553.66 | 28674.14 | 37140.18 | 18733.80 | 20155.59 | 24807.12 | -0.83 | 1.72E-06 | 1.41E-04 |
| ENSMUSG00000038422 | Hdhd3 | 2079.61 | 1992.34 | 2062.13 | 1381.27 | 889.49 | 1186.71 | -0.83 | 1.43E-07 | 1.74E-05 |
| ENSMUSG00000046532 | Ar | 812.15 | 626.03 | 689.91 | 354.20 | 491.79 | 352.76 | -0.83 | 1.03E-05 | 5.91E-04 |
| ENSMUSG00000038188 | Scarf1 | 583.44 | 376.99 | 586.33 | 350.97 | 242.35 | 280.84 | -0.83 | 2.44E-04 | 6.77E-03 |
| ENSMUSG00000089960 | Ugt1a1 | 2717.05 | 3803.04 | 2399.48 | 985.08 | 1686.66 | 2364.87 | -0.82 | 5.89E-04 | 1.28E-02 |
| ENSMUSG00000021287 | Xrcc3 | 339.90 | 246.76 | 275.58 | 193.79 | 151.80 | 142.99 | -0.82 | 9.38E-05 | 3.31E-03 |
| ENSMUSG00000043251 | Exoc3l | 280.60 | 343.86 | 339.25 | 208.86 | 149.14 | 187.51 | -0.82 | 7.87E-05 | 2.90E-03 |
| ENSMUSG00000028214 | Gem | 150.36 | 205.63 | 148.25 | 96.89 | 110.08 | 77.92 | -0.82 | 2.39E-03 | 3.35E-02 |
| ENSMUSG00000057074 | Ces1g | 10447.83 | 18703.31 | 15861.29 | 5227.94 | 10080.01 | 10171.83 | -0.82 | 2.81E-04 | 7.50E-03 |
| ENSMUSG00000053219 | Raet1e | 1055.69 | 903.64 | 1138.45 | 400.49 | 608.97 | 745.76 | -0.82 | 6.07E-05 | 2.38E-03 |
| ENSMUSG00000022615 | Tymp | 7373.94 | 6286.61 | 7550.05 | 4896.35 | 3451.44 | 3710.84 | -0.82 | 3.45E-08 | 5.26E-06 |
| ENSMUSG00000054191 | Klf1 | 316.60 | 191.92 | 214.77 | 129.19 | 124.28 | 157.54 | -0.81 | 1.40E-03 | 2.34E-02 |
| ENSMUSG00000069825 | Gm49340 | 276.36 | 241.05 | 237.57 | 153.95 | 172.22 | 104.46 | -0.81 | 8.16E-04 | 1.62E-02 |
| ENSMUSG00000020108 | Ddit4 | 969.92 | 632.89 | 751.68 | 378.96 | 471.38 | 495.75 | -0.81 | 4.21E-05 | 1.80E-03 |
| ENSMUSG00000069324 | Gm5096 | 1351.11 | 1359.45 | 1686.76 | 1059.37 | 843.33 | 619.90 | -0.80 | 3.55E-05 | 1.58E-03 |
| ENSMUSG00000047230 | Cldn2 | 7545.48 | 9101.47 | 7678.34 | 4460.33 | 3647.62 | 5868.50 | -0.80 | 8.36E-07 | 7.94E-05 |
| ENSMUSG00000031024 | St5 | 3929.45 | 2892.55 | 3399.19 | 2442.79 | 2421.69 | 1020.61 | -0.80 | 8.09E-04 | 1.61E-02 |
| ENSMUSG00000022194 | Pabpn1 | 978.39 | 517.51 | 890.42 | 609.35 | 353.31 | 415.26 | -0.79 | 1.68E-03 | 2.68E-02 |
| ENSMUSG00000055707 | Klhl26 | 702.03 | 698.00 | 555.92 | 473.70 | 405.69 | 250.87 | -0.79 | 2.33E-04 | 6.51E-03 |
| ENSMUSG00000048915 | Efna5 | 412.96 | 378.13 | 437.13 | 248.69 | 265.43 | 196.93 | -0.79 | 5.62E-05 | 2.27E-03 |
| ENSMUSG00000037921 | Ddx60 | 264.72 | 187.35 | 247.08 | 97.97 | 172.22 | 133.57 | -0.79 | 2.32E-03 | 3.29E-02 |
| ENSMUSG00000029804 | Herc3 | 272.13 | 308.45 | 284.14 | 127.04 | 169.55 | 202.07 | -0.79 | 1.80E-04 | 5.34E-03 |
| ENSMUSG00000032125 | Robo4 | 626.85 | 621.46 | 663.30 | 348.82 | 397.70 | 364.75 | -0.78 | 4.88E-05 | 2.03E-03 |
| ENSMUSG00000021259 | Cyp46a1 | 283.78 | 212.49 | 285.09 | 177.64 | 125.17 | 153.26 | -0.78 | 4.91E-04 | 1.14E-02 |
| ENSMUSG00000034687 | Fras1 | 361.07 | 350.72 | 341.15 | 142.11 | 166.00 | 303.96 | -0.78 | 2.09E-03 | 3.09E-02 |
| ENSMUSG00000032012 | Nectin1 | 6117.07 | 4903.16 | 4630.76 | 3673.34 | 3281.00 | 2171.36 | -0.78 | 1.72E-05 | 8.79E-04 |
| ENSMUSG00000056643 | Chst13 | 920.15 | 610.04 | 650.00 | 412.34 | 399.47 | 460.64 | -0.78 | 2.21E-05 | 1.08E-03 |
| ENSMUSG00000027954 | Efna1 | 3775.92 | 3221.56 | 4204.08 | 2296.37 | 1210.84 | 3035.28 | -0.78 | 8.52E-04 | 1.66E-02 |
| ENSMUSG00000026447 | Pik3c2b | 529.43 | 512.94 | 523.61 | 265.92 | 257.44 | 391.29 | -0.77 | 2.08E-04 | 5.99E-03 |
| ENSMUSG00000047656 | Trpt1 | 166.24 | 215.91 | 210.01 | 127.04 | 85.22 | 135.28 | -0.77 | 1.80E-03 | 2.78E-02 |
| ENSMUSG00000020474 | Polm | 275.31 | 233.05 | 186.26 | 132.42 | 136.71 | 137.85 | -0.77 | 1.22E-03 | 2.14E-02 |
| ENSMUSG00000001665 | Gstt3 | 2677.87 | 1911.23 | 2165.71 | 1452.32 | 1379.51 | 1133.63 | -0.77 | 4.06E-06 | 2.84E-04 |
| ENSMUSG00000039395 | Mreg | 3168.13 | 2953.09 | 2822.36 | 1931.41 | 1830.47 | 1500.95 | -0.77 | 6.37E-09 | 1.25E-06 |
| ENSMUSG00000033147 | Slc22a15 | 1443.23 | 1038.44 | 1216.37 | 711.63 | 831.79 | 635.31 | -0.76 | 1.74E-06 | 1.41E-04 |
| ENSMUSG00000041119 | Pde9a | 1503.59 | 1614.21 | 1455.84 | 1108.89 | 1091.00 | 508.59 | -0.76 | 7.66E-04 | 1.56E-02 |
| ENSMUSG00000006356 | Crip2 | 3475.20 | 3780.19 | 4103.35 | 2511.69 | 2594.79 | 1619.96 | -0.76 | 1.45E-05 | 7.79E-04 |
| ENSMUSG00000028464 | Tpm2 | 451.08 | 319.87 | 382.02 | 263.77 | 227.26 | 193.50 | -0.76 | 6.07E-05 | 2.38E-03 |
| ENSMUSG00000026614 | Slc30a10 | 3424.37 | 2772.60 | 2730.18 | 1861.43 | 2016.89 | 1441.87 | -0.75 | 1.81E-06 | 1.45E-04 |
| ENSMUSG00000005320 | Fgfr4 | 5284.80 | 5244.74 | 4885.44 | 3444.02 | 2931.24 | 2819.52 | -0.75 | 1.88E-08 | 3.06E-06 |
| ENSMUSG00000026202 | Tuba4a | 15084.60 | 16792.08 | 12405.09 | 11878.06 | 8965.93 | 5593.65 | -0.74 | 2.94E-04 | 7.68E-03 |
| ENSMUSG00000021338 | Carmil1 | 1832.90 | 1673.61 | 1260.08 | 1093.82 | 910.80 | 849.37 | -0.74 | 9.10E-05 | 3.25E-03 |
| ENSMUSG00000032607 | Amt | 5218.09 | 4856.33 | 4801.82 | 3565.68 | 3172.70 | 2178.21 | -0.74 | 5.36E-06 | 3.53E-04 |
| ENSMUSG00000000552 | Zfp385a | 2082.79 | 2083.73 | 1656.36 | 1376.96 | 1184.21 | 934.99 | -0.74 | 7.43E-05 | 2.80E-03 |
| ENSMUSG00000047617 | Paxx | 1531.12 | 1555.94 | 1908.18 | 1048.60 | 1200.19 | 758.61 | -0.73 | 7.74E-05 | 2.88E-03 |
| ENSMUSG00000036023 | Parp2 | 363.19 | 384.99 | 385.82 | 257.31 | 248.56 | 178.95 | -0.73 | 2.07E-04 | 5.97E-03 |
| ENSMUSG00000064254 | Ethe1 | 8330.10 | 6886.36 | 7760.07 | 5996.63 | 4821.18 | 3069.53 | -0.73 | 1.40E-04 | 4.53E-03 |
| ENSMUSG00000034429 | Zfp707 | 2996.59 | 2710.91 | 2925.94 | 2157.49 | 1456.74 | 1616.53 | -0.72 | 1.63E-06 | 1.36E-04 |
| ENSMUSG00000073434 | Wdr90 | 332.48 | 435.25 | 308.84 | 265.92 | 210.39 | 177.24 | -0.72 | 7.14E-04 | 1.48E-02 |
| ENSMUSG00000004562 | Arhgef40 | 652.26 | 1133.26 | 942.69 | 544.76 | 534.40 | 580.51 | -0.72 | 1.51E-04 | 4.75E-03 |
| ENSMUSG00000018387 | Shroom1 | 3084.48 | 2845.71 | 2519.22 | 1884.04 | 1559.72 | 1701.30 | -0.72 | 9.78E-06 | 5.77E-04 |
| ENSMUSG00000026853 | Crat | 4000.40 | 3502.59 | 2949.70 | 2435.25 | 1864.20 | 2075.47 | -0.71 | 1.59E-05 | 8.32E-04 |
| ENSMUSG00000057342 | Sphk2 | 6366.96 | 6243.19 | 5752.11 | 4646.58 | 3903.29 | 2668.82 | -0.71 | 4.36E-05 | 1.84E-03 |
| ENSMUSG00000028744 | Pqlc2 | 762.38 | 765.41 | 757.38 | 519.99 | 466.94 | 415.26 | -0.71 | 1.14E-06 | 1.02E-04 |
| ENSMUSG00000075324 | Fign | 465.90 | 331.29 | 319.30 | 226.08 | 225.48 | 232.89 | -0.71 | 1.80E-03 | 2.79E-02 |
| ENSMUSG00000020017 | Hal | 25495.37 | 31518.71 | 23875.08 | 17976.96 | 17211.92 | 14552.23 | -0.70 | 2.32E-06 | 1.78E-04 |
| ENSMUSG00000010609 | Psen2 | 19301.01 | 15062.49 | 17455.88 | 13450.96 | 12349.90 | 6107.38 | -0.70 | 1.23E-03 | 2.15E-02 |
| ENSMUSG00000022512 | Cldn1 | 3672.15 | 3371.21 | 3164.47 | 2236.08 | 2760.80 | 1296.31 | -0.70 | 6.24E-04 | 1.34E-02 |
| ENSMUSG00000036073 | Galt | 3375.66 | 3352.93 | 3414.39 | 2506.31 | 1971.62 | 1782.64 | -0.70 | 5.35E-07 | 5.33E-05 |
| ENSMUSG00000072664 | Ugt3a1 | 3494.26 | 3677.37 | 3184.42 | 2317.91 | 2144.72 | 1944.46 | -0.69 | 3.28E-07 | 3.62E-05 |
| ENSMUSG00000036216 | Leap2 | 3768.50 | 2090.58 | 2669.36 | 1790.37 | 1882.85 | 1603.69 | -0.69 | 1.51E-04 | 4.75E-03 |
| ENSMUSG00000040128 | Pnrc1 | 5615.17 | 4956.86 | 4719.14 | 3670.11 | 3285.44 | 2518.13 | -0.69 | 1.93E-05 | 9.60E-04 |
| ENSMUSG00000098176 | Ccdc166 | 309.19 | 223.91 | 275.58 | 183.02 | 150.91 | 168.67 | -0.69 | 1.64E-03 | 2.63E-02 |
| ENSMUSG00000034522 | Zfp395 | 3473.08 | 2909.68 | 2488.81 | 1976.63 | 1596.11 | 1934.19 | -0.69 | 1.57E-05 | 8.29E-04 |
| ENSMUSG00000097867 | Lppos | 399.19 | 299.31 | 364.91 | 234.70 | 222.82 | 203.78 | -0.69 | 2.95E-04 | 7.69E-03 |
| ENSMUSG00000032591 | Mst1 | 11428.34 | 12473.82 | 11034.77 | 8406.04 | 6599.28 | 6705.02 | -0.69 | 1.46E-07 | 1.76E-05 |
| ENSMUSG00000045319 | Proser2 | 2965.88 | 1752.44 | 2152.41 | 1731.16 | 1399.04 | 1144.76 | -0.69 | 4.01E-04 | 9.84E-03 |
| ENSMUSG00000026295 | Spp2 | 4132.75 | 4069.21 | 4474.92 | 2155.34 | 2592.13 | 3162.00 | -0.68 | 7.43E-06 | 4.66E-04 |
| ENSMUSG00000031147 | Magix | 1388.17 | 1425.71 | 1484.35 | 1101.36 | 917.90 | 671.27 | -0.68 | 6.19E-05 | 2.41E-03 |
| ENSMUSG00000026123 | Plekhb2 | 1450.65 | 1493.11 | 1414.03 | 867.73 | 1021.76 | 839.09 | -0.68 | 2.63E-07 | 3.02E-05 |
| ENSMUSG00000058620 | Adra2b | 338.84 | 431.83 | 370.61 | 245.46 | 255.66 | 216.62 | -0.67 | 5.56E-04 | 1.24E-02 |
| ENSMUSG00000036120 | Rfxank | 490.25 | 479.81 | 513.16 | 417.72 | 254.77 | 263.71 | -0.67 | 1.49E-03 | 2.46E-02 |
| ENSMUSG00000071633 | Gm4952 | 8407.39 | 7836.84 | 7474.98 | 4771.46 | 4866.46 | 5347.06 | -0.66 | 8.00E-08 | 1.10E-05 |
| ENSMUSG00000047150 | 1700001C19Rik | 634.26 | 653.45 | 684.21 | 458.63 | 411.01 | 378.45 | -0.66 | 1.32E-05 | 7.16E-04 |
| ENSMUSG00000057933 | Gsta2 | 1847.72 | 2097.44 | 1843.56 | 1212.24 | 1448.75 | 1005.20 | -0.66 | 1.27E-04 | 4.24E-03 |
| ENSMUSG00000015468 | Notch4 | 322.95 | 299.31 | 391.52 | 190.56 | 225.48 | 226.90 | -0.66 | 2.68E-03 | 3.66E-02 |
| ENSMUSG00000038539 | Atf5 | 25443.49 | 18838.11 | 22775.60 | 16807.78 | 16167.08 | 9633.28 | -0.65 | 8.71E-04 | 1.69E-02 |
| ENSMUSG00000101249 | Gm29216 | 620.50 | 595.19 | 495.10 | 412.34 | 291.17 | 386.15 | -0.65 | 1.57E-03 | 2.55E-02 |
| ENSMUSG00000020427 | Igfbp3 | 1073.69 | 1286.34 | 1155.55 | 456.48 | 887.72 | 893.89 | -0.65 | 2.56E-03 | 3.53E-02 |
| ENSMUSG00000003190 | Bcl2l12 | 273.19 | 218.20 | 281.29 | 170.10 | 178.43 | 144.70 | -0.65 | 2.26E-03 | 3.25E-02 |
| ENSMUSG00000037541 | Shank2 | 1414.64 | 1384.58 | 974.05 | 929.10 | 862.86 | 614.76 | -0.65 | 4.21E-04 | 1.03E-02 |
| ENSMUSG00000074768 | Bhmt | 152887.52 | 139097.01 | 187188.12 | 112776.39 | 118197.59 | 74650.82 | -0.65 | 1.25E-04 | 4.21E-03 |
| ENSMUSG00000002661 | Alkbh7 | 1386.06 | 1078.42 | 1258.18 | 1000.16 | 791.84 | 589.08 | -0.65 | 5.43E-04 | 1.23E-02 |
| ENSMUSG00000033066 | Gas7 | 675.56 | 750.55 | 790.64 | 502.77 | 604.53 | 309.95 | -0.65 | 2.47E-03 | 3.43E-02 |
| ENSMUSG00000028494 | Plin2 | 42413.93 | 32839.32 | 39823.80 | 26686.60 | 24816.10 | 22179.39 | -0.64 | 1.34E-05 | 7.25E-04 |
| ENSMUSG00000010651 | Acaa1b | 129119.16 | 111425.89 | 129431.39 | 85899.23 | 62310.55 | 88997.56 | -0.64 | 1.28E-05 | 7.00E-04 |
| ENSMUSG00000000957 | Mmp14 | 2740.34 | 2890.26 | 2408.99 | 1747.31 | 1818.04 | 1591.70 | -0.64 | 4.42E-05 | 1.86E-03 |
| ENSMUSG00000030316 | Tamm41 | 243.54 | 223.91 | 215.72 | 160.41 | 142.03 | 136.99 | -0.64 | 4.04E-03 | 4.84E-02 |
| ENSMUSG00000004043 | Stat5a | 1272.76 | 1176.67 | 964.54 | 761.15 | 863.75 | 570.24 | -0.64 | 5.01E-04 | 1.16E-02 |
| ENSMUSG00000021134 | Srsf5 | 6898.51 | 5701.70 | 6576.01 | 4876.97 | 2892.18 | 4579.89 | -0.64 | 3.04E-04 | 7.85E-03 |
| ENSMUSG00000033022 | Cdo1 | 74384.27 | 78095.34 | 75934.82 | 45908.10 | 52177.27 | 49074.82 | -0.63 | 1.18E-07 | 1.52E-05 |
| ENSMUSG00000030701 | Plekhb1 | 3753.68 | 4293.12 | 3374.48 | 3562.45 | 1970.73 | 1831.44 | -0.63 | 2.25E-03 | 3.25E-02 |
| ENSMUSG00000040234 | Tm7sf3 | 4409.12 | 4239.43 | 3938.00 | 2707.63 | 2799.86 | 2605.46 | -0.63 | 4.69E-08 | 6.74E-06 |
| ENSMUSG00000027559 | Car3 | 280701.10 | 241354.03 | 224673.25 | 180090.39 | 143519.68 | 157942.57 | -0.63 | 1.03E-06 | 9.38E-05 |
| ENSMUSG00000020553 | Pctp | 10302.76 | 8421.74 | 8679.00 | 6376.66 | 5377.78 | 5948.13 | -0.63 | 2.43E-06 | 1.84E-04 |
| ENSMUSG00000031903 | Pla2g15 | 1534.30 | 1543.38 | 1537.57 | 956.01 | 1137.16 | 890.46 | -0.63 | 6.75E-05 | 2.57E-03 |
| ENSMUSG00000004996 | Mri1 | 2479.86 | 1850.68 | 2075.43 | 1635.35 | 1315.59 | 1197.84 | -0.63 | 5.79E-05 | 2.31E-03 |
| ENSMUSG00000085148 | Mir22hg | 1095.93 | 880.79 | 1091.88 | 759.00 | 480.25 | 752.61 | -0.62 | 9.17E-04 | 1.75E-02 |
| ENSMUSG00000037621 | Atoh8 | 1957.84 | 1980.91 | 2557.23 | 1395.27 | 1850.89 | 976.94 | -0.62 | 2.49E-03 | 3.46E-02 |
| ENSMUSG00000055401 | Fbxo6 | 2492.57 | 2431.02 | 2481.21 | 1737.62 | 1707.97 | 1375.08 | -0.62 | 3.51E-06 | 2.51E-04 |
| ENSMUSG00000045594 | Glb1 | 681.91 | 668.30 | 567.32 | 398.34 | 297.38 | 551.40 | -0.62 | 2.55E-03 | 3.53E-02 |
| ENSMUSG00000030795 | Fus | 4089.34 | 4281.70 | 4899.70 | 3017.69 | 3015.57 | 2616.59 | -0.62 | 1.28E-06 | 1.11E-04 |
| ENSMUSG00000045314 | Sowahb | 689.32 | 548.35 | 535.96 | 398.34 | 351.54 | 406.70 | -0.62 | 1.30E-03 | 2.24E-02 |
| ENSMUSG00000003228 | Grk5 | 374.84 | 368.99 | 400.07 | 200.25 | 324.90 | 220.05 | -0.62 | 2.74E-03 | 3.70E-02 |
| ENSMUSG00000036078 | Sigmar1 | 16190.06 | 13789.86 | 13391.49 | 11185.81 | 10270.87 | 6835.16 | -0.62 | 2.11E-04 | 6.04E-03 |
| ENSMUSG00000038351 | Sgsm2 | 1008.04 | 1170.96 | 1011.11 | 680.41 | 728.81 | 671.27 | -0.62 | 1.78E-05 | 8.99E-04 |
| ENSMUSG00000000154 | Slc22a18 | 7993.38 | 8107.59 | 7929.22 | 6277.62 | 5563.32 | 3899.20 | -0.61 | 9.53E-05 | 3.35E-03 |
| ENSMUSG00000053559 | Smagp | 2375.04 | 2465.29 | 2453.65 | 1900.19 | 1750.58 | 1137.05 | -0.61 | 1.75E-03 | 2.73E-02 |
| ENSMUSG00000031217 | Efnb1 | 1814.90 | 1503.39 | 1343.71 | 1108.89 | 1077.69 | 873.34 | -0.61 | 7.83E-05 | 2.89E-03 |
| ENSMUSG00000025200 | Cwf19l1 | 1612.65 | 1589.07 | 1457.74 | 1079.82 | 1122.07 | 857.93 | -0.61 | 2.47E-05 | 1.18E-03 |
| ENSMUSG00000030731 | Syt3 | 698.85 | 664.87 | 519.81 | 535.07 | 393.26 | 309.95 | -0.61 | 3.72E-03 | 4.57E-02 |
| ENSMUSG00000028121 | Bcar3 | 4053.34 | 3544.86 | 3670.97 | 2450.33 | 2464.30 | 2488.16 | -0.61 | 1.38E-06 | 1.18E-04 |
| ENSMUSG00000043681 | Fam25c | 1919.72 | 1190.38 | 1668.71 | 1155.18 | 1000.46 | 986.36 | -0.61 | 1.81E-03 | 2.79E-02 |
| ENSMUSG00000066258 | Trim12a | 372.72 | 323.30 | 360.16 | 207.78 | 271.64 | 214.91 | -0.60 | 3.08E-03 | 4.02E-02 |
| ENSMUSG00000004018 | Fancl | 457.43 | 386.13 | 445.69 | 290.68 | 316.03 | 243.17 | -0.60 | 1.70E-03 | 2.69E-02 |
| ENSMUSG00000034947 | Tmem106a | 454.25 | 494.66 | 480.85 | 328.36 | 276.97 | 337.35 | -0.60 | 5.48E-04 | 1.24E-02 |
| ENSMUSG00000001053 | N4bp3 | 648.03 | 471.81 | 450.44 | 398.34 | 357.75 | 280.84 | -0.60 | 3.00E-03 | 3.94E-02 |
| ENSMUSG00000020743 | Mif4gd | 4196.29 | 4214.30 | 4376.09 | 3326.67 | 2718.19 | 2398.26 | -0.60 | 1.92E-05 | 9.54E-04 |
| ENSMUSG00000006395 | Hyi | 4942.79 | 3589.41 | 5015.63 | 3126.43 | 2367.54 | 3452.26 | -0.60 | 3.13E-04 | 8.03E-03 |
| ENSMUSG00000098747 | Gm27216 | 742.26 | 606.61 | 706.07 | 469.39 | 434.98 | 453.79 | -0.60 | 6.22E-05 | 2.42E-03 |
| ENSMUSG00000033107 | Rnf125 | 4122.16 | 3788.19 | 2683.62 | 2032.61 | 2419.91 | 2552.38 | -0.60 | 4.22E-04 | 1.03E-02 |
| ENSMUSG00000035561 | Aldh1b1 | 6676.15 | 5724.55 | 6565.55 | 4280.54 | 4687.14 | 3589.25 | -0.60 | 1.93E-04 | 5.62E-03 |
| ENSMUSG00000048772 | Tmem53 | 1271.70 | 1326.32 | 1280.99 | 977.55 | 729.70 | 869.06 | -0.59 | 4.97E-05 | 2.05E-03 |
| ENSMUSG00000008167 | Fbxw9 | 2323.15 | 1499.97 | 1992.76 | 1458.78 | 1241.03 | 1162.74 | -0.59 | 4.41E-04 | 1.05E-02 |
| ENSMUSG00000026923 | Notch1 | 1637.01 | 1152.68 | 1023.46 | 717.01 | 1001.34 | 811.69 | -0.59 | 1.78E-03 | 2.76E-02 |
| ENSMUSG00000031451 | Gas6 | 2784.82 | 2405.89 | 2416.59 | 1263.92 | 1556.17 | 2233.01 | -0.59 | 1.73E-03 | 2.71E-02 |
| ENSMUSG00000060216 | Arrb2 | 828.03 | 708.29 | 827.70 | 620.12 | 488.24 | 465.78 | -0.59 | 2.83E-04 | 7.51E-03 |
| ENSMUSG00000046794 | Ppp1r3b | 19855.85 | 13433.44 | 15868.89 | 10868.21 | 9814.59 | 12015.27 | -0.59 | 1.05E-04 | 3.63E-03 |
| ENSMUSG00000023495 | Pcbp4 | 1112.87 | 1246.35 | 1260.08 | 903.26 | 844.22 | 662.71 | -0.59 | 4.69E-04 | 1.10E-02 |
| ENSMUSG00000021376 | Tpmt | 3211.54 | 1729.59 | 3297.51 | 1744.08 | 2012.45 | 1731.27 | -0.59 | 3.32E-03 | 4.20E-02 |
| ENSMUSG00000003849 | Nqo1 | 355.78 | 370.14 | 334.50 | 199.17 | 255.66 | 250.01 | -0.59 | 1.20E-03 | 2.11E-02 |
| ENSMUSG00000064225 | Paqr9 | 35243.29 | 32712.51 | 33735.29 | 22161.68 | 16834.64 | 28779.96 | -0.59 | 7.82E-04 | 1.58E-02 |
| ENSMUSG00000027983 | Cyp2u1 | 3549.32 | 3288.96 | 3452.40 | 2774.38 | 2071.04 | 2022.38 | -0.58 | 4.38E-04 | 1.05E-02 |
| ENSMUSG00000020182 | Ddc | 6495.08 | 6681.87 | 5702.69 | 3871.43 | 3801.20 | 4927.52 | -0.58 | 8.16E-05 | 2.96E-03 |
| ENSMUSG00000075225 | Ccdc162 | 598.26 | 476.38 | 576.83 | 305.75 | 431.43 | 364.75 | -0.58 | 2.83E-03 | 3.77E-02 |
| ENSMUSG00000037103 | Dcaf15 | 399.19 | 314.16 | 420.03 | 290.68 | 229.92 | 238.03 | -0.58 | 4.01E-03 | 4.83E-02 |
| ENSMUSG00000021884 | Hacl1 | 22894.80 | 30106.71 | 22882.03 | 19444.35 | 14034.79 | 17455.66 | -0.58 | 1.58E-04 | 4.93E-03 |
| ENSMUSG00000031775 | Pllp | 1029.22 | 1044.15 | 1107.09 | 700.86 | 659.57 | 777.44 | -0.57 | 5.59E-04 | 1.24E-02 |
| ENSMUSG00000006522 | Itih3 | 36749.00 | 64185.53 | 38355.60 | 30458.98 | 33342.61 | 29842.52 | -0.57 | 1.52E-03 | 2.49E-02 |
| ENSMUSG00000049115 | Agtr1a | 2821.88 | 2877.70 | 2280.70 | 1932.49 | 1787.86 | 1649.93 | -0.57 | 3.12E-05 | 1.41E-03 |
| ENSMUSG00000032492 | Pth1r | 783.56 | 998.45 | 845.76 | 586.74 | 611.64 | 572.81 | -0.57 | 1.87E-04 | 5.48E-03 |
| ENSMUSG00000040413 | Timd2 | 4932.20 | 5285.87 | 4650.72 | 3319.14 | 3432.80 | 3275.02 | -0.57 | 4.29E-07 | 4.46E-05 |
| ENSMUSG00000070576 | Mn1 | 2890.70 | 2081.45 | 1561.33 | 1593.36 | 1530.42 | 1284.32 | -0.57 | 3.04E-03 | 3.98E-02 |
| ENSMUSG00000063511 | Snrnp70 | 4296.88 | 2949.67 | 4196.48 | 3258.85 | 2395.95 | 2075.47 | -0.57 | 1.75E-03 | 2.73E-02 |
| ENSMUSG00000066357 | Wdr6 | 1639.12 | 1796.99 | 1536.62 | 1053.98 | 1027.97 | 1277.47 | -0.57 | 9.10E-05 | 3.25E-03 |
| ENSMUSG00000044876 | Zfp444 | 2391.98 | 1784.42 | 2063.08 | 1674.10 | 1426.56 | 1121.64 | -0.56 | 7.83E-04 | 1.58E-02 |
| ENSMUSG00000045287 | Rtn4rl1 | 1566.06 | 1350.31 | 948.39 | 921.56 | 845.99 | 849.37 | -0.56 | 3.42E-03 | 4.28E-02 |
| ENSMUSG00000036151 | Tm6sf2 | 1727.01 | 1769.57 | 1452.04 | 1289.76 | 1013.77 | 1049.72 | -0.56 | 9.23E-05 | 3.28E-03 |
| ENSMUSG00000055912 | Tmem150a | 6982.16 | 7246.22 | 6997.94 | 6202.25 | 4568.19 | 3623.50 | -0.56 | 6.92E-04 | 1.45E-02 |
| ENSMUSG00000031513 | Leprotl1 | 1230.40 | 1300.05 | 1311.40 | 812.83 | 1065.26 | 727.78 | -0.56 | 4.01E-04 | 9.84E-03 |
| ENSMUSG00000024039 | Cbs | 47094.12 | 50094.07 | 42844.77 | 39360.25 | 34802.90 | 21341.16 | -0.55 | 2.31E-03 | 3.28E-02 |
| ENSMUSG00000041926 | Rnpep | 2653.52 | 2760.03 | 2442.25 | 1708.55 | 1865.98 | 1783.50 | -0.55 | 2.07E-06 | 1.63E-04 |
| ENSMUSG00000042286 | Stab1 | 3160.71 | 3485.45 | 3348.82 | 2383.58 | 2173.13 | 2264.69 | -0.55 | 1.71E-05 | 8.74E-04 |
| ENSMUSG00000044279 | Crb3 | 1128.75 | 961.90 | 921.78 | 784.84 | 571.69 | 701.24 | -0.55 | 1.37E-03 | 2.31E-02 |
| ENSMUSG00000011263 | Exoc3l2 | 633.20 | 581.48 | 658.55 | 454.32 | 386.16 | 440.95 | -0.55 | 1.64E-03 | 2.63E-02 |
| ENSMUSG00000053279 | Aldh1a1 | 88276.59 | 98960.06 | 82186.78 | 63514.70 | 68066.50 | 52737.71 | -0.55 | 2.22E-05 | 1.08E-03 |
| ENSMUSG00000028411 | Aptx | 754.97 | 654.59 | 721.27 | 578.13 | 383.49 | 499.17 | -0.55 | 1.95E-03 | 2.93E-02 |
| ENSMUSG00000024193 | Phf1 | 1053.57 | 1098.99 | 1257.23 | 986.16 | 614.30 | 737.20 | -0.55 | 1.87E-03 | 2.85E-02 |
| ENSMUSG00000002963 | Pnkp | 2746.70 | 2301.93 | 2780.55 | 2078.90 | 1742.59 | 1546.32 | -0.55 | 1.34E-04 | 4.40E-03 |
| ENSMUSG00000078317 | F8a | 1344.76 | 1090.99 | 1195.46 | 1076.59 | 651.58 | 763.74 | -0.54 | 2.99E-03 | 3.94E-02 |
| ENSMUSG00000035413 | Tmem98 | 698.85 | 685.44 | 728.87 | 381.11 | 466.05 | 600.21 | -0.54 | 2.44E-03 | 3.40E-02 |
| ENSMUSG00000030805 | Stx4a | 2148.44 | 2086.02 | 2541.08 | 1929.26 | 1538.41 | 1183.29 | -0.54 | 1.52E-03 | 2.49E-02 |
| ENSMUSG00000073600 | Prob1 | 1200.75 | 941.33 | 815.35 | 686.87 | 651.58 | 691.82 | -0.54 | 2.35E-03 | 3.32E-02 |
| ENSMUSG00000049858 | Suox | 12983.81 | 12791.41 | 11209.62 | 9226.41 | 8770.63 | 7414.82 | -0.54 | 1.13E-05 | 6.34E-04 |
| ENSMUSG00000039646 | Vasn | 501.90 | 579.19 | 666.15 | 414.49 | 388.82 | 398.14 | -0.54 | 9.02E-04 | 1.73E-02 |
| ENSMUSG00000022579 | Gpihbp1 | 927.57 | 796.25 | 1040.57 | 697.63 | 654.25 | 549.69 | -0.54 | 2.48E-03 | 3.44E-02 |
| ENSMUSG00000051768 | Xrcc1 | 1084.28 | 795.11 | 985.45 | 784.84 | 660.46 | 527.43 | -0.54 | 2.78E-03 | 3.73E-02 |
| ENSMUSG00000068762 | Gstm6 | 9150.72 | 6648.75 | 8165.84 | 5359.28 | 5507.39 | 5618.48 | -0.54 | 3.80E-05 | 1.67E-03 |
| ENSMUSG00000027222 | Pex16 | 7864.20 | 7030.31 | 8129.73 | 6127.97 | 4823.85 | 4905.26 | -0.54 | 4.64E-05 | 1.94E-03 |
| ENSMUSG00000061028 | Clasrp | 512.49 | 444.39 | 511.26 | 401.57 | 270.75 | 340.77 | -0.54 | 4.20E-03 | 4.99E-02 |
| ENSMUSG00000048782 | Insc | 1875.25 | 2121.43 | 1703.87 | 1628.89 | 1351.10 | 952.11 | -0.54 | 3.03E-03 | 3.97E-02 |
| ENSMUSG00000020865 | Abcc3 | 23862.60 | 21471.33 | 19919.03 | 12845.91 | 12625.09 | 19518.28 | -0.54 | 1.18E-03 | 2.09E-02 |
| ENSMUSG00000024906 | Mus81 | 672.38 | 586.05 | 629.09 | 467.24 | 406.57 | 428.96 | -0.54 | 2.80E-04 | 7.49E-03 |
| ENSMUSG00000021928 | Ebpl | 7481.95 | 5197.90 | 7941.57 | 4956.64 | 3859.79 | 5409.57 | -0.54 | 1.59E-03 | 2.58E-02 |
| ENSMUSG00000059895 | Ptp4a3 | 549.55 | 466.10 | 590.13 | 329.44 | 411.90 | 366.46 | -0.54 | 1.91E-03 | 2.89E-02 |
| ENSMUSG00000036892 | Prodh2 | 18294.02 | 17422.68 | 19850.61 | 15122.91 | 11174.57 | 12053.80 | -0.54 | 8.41E-05 | 3.04E-03 |
| ENSMUSG00000042289 | Hsd3b7 | 36600.76 | 42196.69 | 39002.75 | 33037.42 | 28787.74 | 19725.48 | -0.53 | 1.14E-03 | 2.03E-02 |
| ENSMUSG00000016520 | Lnx2 | 1699.48 | 1465.69 | 1387.42 | 1028.15 | 1095.44 | 1030.88 | -0.53 | 9.81E-05 | 3.44E-03 |
| ENSMUSG00000027800 | Tm4sf1 | 549.55 | 766.55 | 572.07 | 384.34 | 479.37 | 443.52 | -0.53 | 3.90E-03 | 4.72E-02 |
| ENSMUSG00000026389 | Steap3 | 2160.09 | 2257.37 | 1973.75 | 1492.16 | 1830.47 | 1121.64 | -0.52 | 1.61E-03 | 2.60E-02 |
| ENSMUSG00000035206 | Sppl2b | 1084.28 | 913.92 | 967.40 | 754.69 | 605.42 | 703.81 | -0.52 | 3.37E-04 | 8.56E-03 |
| ENSMUSG00000036986 | Pml | 1329.94 | 1369.73 | 1200.22 | 839.74 | 1088.34 | 786.86 | -0.52 | 2.12E-03 | 3.12E-02 |
| ENSMUSG00000045092 | S1pr1 | 3886.04 | 4161.75 | 3479.96 | 2412.65 | 2474.06 | 3142.31 | -0.52 | 1.77E-04 | 5.32E-03 |
| ENSMUSG00000029810 | Tmem176b | 13323.71 | 17322.15 | 15070.65 | 10391.28 | 12288.65 | 9187.19 | -0.52 | 2.59E-04 | 7.10E-03 |
| ENSMUSG00000028327 | Stra6l | 9363.55 | 10963.58 | 9097.13 | 7907.58 | 7738.22 | 4871.86 | -0.52 | 3.07E-03 | 4.01E-02 |
| ENSMUSG00000019470 | Xab2 | 1439.00 | 1565.08 | 1535.67 | 1087.36 | 949.86 | 1136.20 | -0.52 | 5.52E-05 | 2.24E-03 |
| ENSMUSG00000049482 | Ctu2 | 1087.46 | 984.75 | 1030.11 | 849.43 | 616.96 | 707.23 | -0.51 | 1.00E-03 | 1.87E-02 |
| ENSMUSG00000042487 | Leo1 | 1242.05 | 1265.77 | 1204.97 | 907.57 | 1008.45 | 684.12 | -0.51 | 1.38E-03 | 2.32E-02 |
| ENSMUSG00000030034 | Ino80b | 2638.69 | 2060.88 | 2712.13 | 2139.19 | 1562.38 | 1492.38 | -0.51 | 1.72E-03 | 2.70E-02 |
| ENSMUSG00000026544 | Dusp23 | 1048.28 | 1098.99 | 1179.31 | 948.48 | 722.60 | 664.42 | -0.51 | 1.72E-03 | 2.71E-02 |
| ENSMUSG00000032579 | Hemk1 | 1273.82 | 1175.53 | 1209.72 | 933.41 | 785.63 | 850.22 | -0.51 | 4.66E-04 | 1.10E-02 |
| ENSMUSG00000027649 | Ctnnbl1 | 1436.88 | 1328.61 | 1262.94 | 972.16 | 897.48 | 958.96 | -0.51 | 8.12E-05 | 2.96E-03 |
| ENSMUSG00000031534 | Smim19 | 1154.16 | 1114.98 | 1354.16 | 907.57 | 785.63 | 853.65 | -0.51 | 2.21E-04 | 6.22E-03 |
| ENSMUSG00000028670 | Lypla2 | 6092.71 | 5002.55 | 5945.01 | 5122.43 | 3672.48 | 3181.70 | -0.51 | 3.13E-03 | 4.05E-02 |
| ENSMUSG00000039450 | Dcxr | 10442.53 | 9953.70 | 11073.73 | 9074.61 | 6569.98 | 6485.83 | -0.51 | 3.95E-04 | 9.74E-03 |
| ENSMUSG00000030109 | Slc6a12 | 4740.54 | 5540.62 | 4602.25 | 3945.71 | 3496.71 | 3025.01 | -0.51 | 1.62E-04 | 5.00E-03 |
| ENSMUSG00000023367 | Tmem176a | 8416.92 | 11695.85 | 10261.23 | 6572.60 | 8204.27 | 6589.43 | -0.51 | 5.52E-04 | 1.24E-02 |
| ENSMUSG00000026922 | Agpat2 | 19256.53 | 16591.02 | 18216.11 | 14559.85 | 11900.72 | 11587.16 | -0.51 | 5.72E-05 | 2.30E-03 |
| ENSMUSG00000024325 | Ring1 | 1810.66 | 1391.44 | 1715.27 | 1358.66 | 1070.59 | 1037.73 | -0.51 | 1.72E-03 | 2.71E-02 |
| ENSMUSG00000000555 | Itga5 | 1820.19 | 1522.81 | 1503.36 | 1117.50 | 1178.00 | 1119.93 | -0.50 | 1.05E-04 | 3.63E-03 |
| ENSMUSG00000070473 | Cldn3 | 12055.19 | 10263.29 | 11259.99 | 9667.81 | 6971.23 | 7067.20 | -0.50 | 5.63E-04 | 1.25E-02 |
| ENSMUSG00000040471 | Ggt6 | 1839.25 | 1781.00 | 1546.12 | 1454.48 | 1107.87 | 1086.54 | -0.50 | 1.67E-03 | 2.67E-02 |
| ENSMUSG00000062960 | Kdr | 2425.86 | 3010.21 | 2501.16 | 1599.82 | 1666.24 | 2340.89 | -0.50 | 2.69E-03 | 3.67E-02 |
| ENSMUSG00000018841 | Rad51d | 903.21 | 799.68 | 843.86 | 628.73 | 558.37 | 613.91 | -0.50 | 2.30E-04 | 6.45E-03 |
| ENSMUSG00000017721 | Pigt | 5075.14 | 5398.96 | 4796.11 | 4122.28 | 3580.16 | 3102.07 | -0.50 | 1.35E-04 | 4.42E-03 |
| ENSMUSG00000045867 | Cradd | 752.85 | 763.12 | 665.20 | 553.37 | 504.22 | 486.33 | -0.50 | 9.22E-04 | 1.75E-02 |
| ENSMUSG00000024014 | Pim1 | 2919.29 | 2865.13 | 3038.08 | 2286.68 | 2088.80 | 1878.54 | -0.50 | 1.50E-04 | 4.73E-03 |
| ENSMUSG00000026272 | Agxt | 23082.22 | 24875.68 | 21974.50 | 18152.44 | 15979.77 | 15444.41 | -0.50 | 1.49E-05 | 7.91E-04 |
| ENSMUSG00000031310 | Zmym3 | 628.97 | 731.13 | 595.83 | 521.07 | 415.45 | 451.23 | -0.50 | 2.76E-03 | 3.72E-02 |
| ENSMUSG00000028412 | Slc44a1 | 3482.61 | 3250.12 | 2984.86 | 2072.44 | 2452.76 | 2376.85 | -0.49 | 1.83E-04 | 5.41E-03 |
| ENSMUSG00000036430 | Tbcc | 993.22 | 1003.02 | 1001.61 | 704.09 | 648.03 | 779.16 | -0.49 | 2.64E-04 | 7.19E-03 |
| ENSMUSG00000016382 | Pls3 | 4677.01 | 5730.26 | 4389.39 | 3115.66 | 3740.83 | 3671.45 | -0.49 | 3.80E-04 | 9.41E-03 |
| ENSMUSG00000020211 | Sf3a2 | 1146.75 | 1087.56 | 1102.34 | 891.42 | 742.13 | 748.33 | -0.49 | 1.03E-03 | 1.89E-02 |
| ENSMUSG00000001663 | Gstt1 | 14936.36 | 16307.70 | 17438.77 | 13429.43 | 11175.45 | 10150.43 | -0.49 | 2.15E-04 | 6.11E-03 |
| ENSMUSG00000025279 | Dnase1l3 | 2945.76 | 3908.14 | 3475.21 | 2499.85 | 2472.29 | 2404.25 | -0.49 | 1.12E-03 | 2.01E-02 |
| ENSMUSG00000045316 | Fahd1 | 5133.38 | 4870.03 | 5304.52 | 3835.90 | 3030.66 | 4079.01 | -0.48 | 3.40E-04 | 8.64E-03 |
| ENSMUSG00000026688 | Mgst3 | 973.10 | 816.81 | 1050.07 | 777.30 | 558.37 | 698.67 | -0.48 | 3.73E-03 | 4.59E-02 |
| ENSMUSG00000029111 | Nelfa | 632.14 | 731.13 | 734.57 | 473.70 | 478.48 | 552.26 | -0.48 | 2.31E-03 | 3.28E-02 |
| ENSMUSG00000037204 | Atg101 | 3468.84 | 2705.19 | 3312.71 | 2585.98 | 2256.57 | 1970.15 | -0.48 | 9.13E-04 | 1.74E-02 |
| ENSMUSG00000032531 | Amotl2 | 3550.38 | 3737.92 | 2972.51 | 2567.68 | 2229.05 | 2572.07 | -0.48 | 1.01E-03 | 1.88E-02 |
| ENSMUSG00000040146 | Rgl3 | 2288.21 | 2423.02 | 2116.30 | 1793.60 | 1328.91 | 1790.35 | -0.47 | 1.03E-03 | 1.89E-02 |
| ENSMUSG00000037797 | Adh4 | 11320.33 | 13424.30 | 11510.86 | 8721.48 | 10246.02 | 7154.53 | -0.47 | 1.82E-03 | 2.80E-02 |
| ENSMUSG00000073889 | Il11ra1 | 3194.60 | 2865.13 | 3164.47 | 2694.71 | 2099.45 | 1858.84 | -0.47 | 1.37E-03 | 2.32E-02 |
| ENSMUSG00000025494 | Sigirr | 1785.25 | 1537.67 | 1568.93 | 1332.82 | 1195.75 | 1000.92 | -0.47 | 9.86E-04 | 1.84E-02 |
| ENSMUSG00000025188 | Hps1 | 918.04 | 916.20 | 771.64 | 627.65 | 624.06 | 630.17 | -0.47 | 6.78E-04 | 1.43E-02 |
| ENSMUSG00000028393 | Alad | 11573.40 | 10989.85 | 11145.00 | 9634.43 | 7310.34 | 7418.25 | -0.47 | 3.12E-04 | 8.02E-03 |
| ENSMUSG00000028669 | Pithd1 | 2016.08 | 1918.08 | 2062.13 | 1565.37 | 1351.99 | 1418.75 | -0.47 | 5.43E-04 | 1.23E-02 |
| ENSMUSG00000001670 | Tat | 119200.76 | 83785.62 | 84124.42 | 68786.78 | 74956.95 | 63802.58 | -0.47 | 1.48E-03 | 2.46E-02 |
| ENSMUSG00000035459 | Stab2 | 2187.62 | 2612.66 | 2446.05 | 1806.52 | 1477.16 | 1955.60 | -0.47 | 2.21E-03 | 3.22E-02 |
| ENSMUSG00000025792 | Slc25a10 | 25709.26 | 20793.89 | 24876.69 | 18775.79 | 13580.28 | 19324.77 | -0.47 | 2.42E-03 | 3.39E-02 |
| ENSMUSG00000005681 | Apoa2 | 481023.69 | 427595.44 | 439931.03 | 353945.15 | 296429.63 | 326575.84 | -0.47 | 1.43E-04 | 4.59E-03 |
| ENSMUSG00000005803 | Sqor | 10380.06 | 12395.00 | 10201.36 | 7709.49 | 7234.88 | 8945.73 | -0.46 | 3.30E-04 | 8.45E-03 |
| ENSMUSG00000074457 | S100a16 | 1644.42 | 1429.14 | 1605.99 | 1289.76 | 1095.44 | 1009.48 | -0.46 | 7.43E-04 | 1.53E-02 |
| ENSMUSG00000034274 | Thoc5 | 1080.04 | 1357.17 | 1269.59 | 950.63 | 872.62 | 864.78 | -0.46 | 8.27E-04 | 1.64E-02 |
| ENSMUSG00000026380 | Tfcp2l1 | 450.02 | 454.67 | 421.93 | 315.44 | 324.90 | 321.08 | -0.46 | 3.40E-03 | 4.26E-02 |
| ENSMUSG00000000876 | Pxmp4 | 8263.39 | 7603.79 | 7918.77 | 6412.19 | 5712.45 | 5124.45 | -0.46 | 4.02E-04 | 9.85E-03 |
| ENSMUSG00000022453 | Naga | 1875.25 | 1954.64 | 1760.89 | 1425.41 | 1395.49 | 1243.22 | -0.46 | 1.54E-04 | 4.82E-03 |
| ENSMUSG00000029630 | Cyp3a25 | 25333.36 | 23527.65 | 19794.54 | 16194.12 | 15725.00 | 17984.80 | -0.46 | 2.18E-04 | 6.18E-03 |
| ENSMUSG00000039041 | Adrm1 | 10451.01 | 9158.59 | 10188.06 | 8524.47 | 6311.66 | 6869.41 | -0.46 | 1.94E-03 | 2.92E-02 |
| ENSMUSG00000073609 | D2hgdh | 2024.55 | 2168.27 | 2127.70 | 1459.86 | 1548.18 | 1601.12 | -0.46 | 6.47E-05 | 2.50E-03 |
| ENSMUSG00000038195 | Rilp | 1661.36 | 1680.46 | 1764.69 | 1395.27 | 1101.66 | 1232.09 | -0.45 | 4.67E-04 | 1.10E-02 |
| ENSMUSG00000020592 | Sdc1 | 23595.77 | 19746.32 | 19042.86 | 16771.17 | 13498.61 | 15261.18 | -0.45 | 6.47E-04 | 1.38E-02 |
| ENSMUSG00000024914 | Drap1 | 7329.47 | 6207.78 | 6562.70 | 5857.74 | 4482.08 | 4341.87 | -0.45 | 2.85E-03 | 3.80E-02 |
| ENSMUSG00000022241 | Tars | 11838.12 | 11082.38 | 10758.23 | 8876.51 | 8944.62 | 6778.65 | -0.45 | 5.71E-04 | 1.25E-02 |
| ENSMUSG00000032109 | Nlrx1 | 1591.48 | 1558.23 | 1488.15 | 1118.58 | 1260.56 | 1008.62 | -0.45 | 5.53E-04 | 1.24E-02 |
| ENSMUSG00000015890 | Amdhd1 | 18207.20 | 19417.31 | 17709.60 | 15006.64 | 13205.66 | 12213.05 | -0.45 | 1.63E-04 | 5.00E-03 |
| ENSMUSG00000049940 | Pgrmc2 | 8852.12 | 6613.33 | 7517.74 | 6096.75 | 5571.30 | 5127.02 | -0.45 | 6.92E-04 | 1.45E-02 |
| ENSMUSG00000024007 | Ppil1 | 1270.64 | 951.62 | 1169.81 | 850.51 | 873.51 | 756.89 | -0.45 | 3.09E-03 | 4.02E-02 |
| ENSMUSG00000047423 | AI837181 | 1887.96 | 1707.88 | 1960.45 | 1571.83 | 1238.36 | 1262.06 | -0.45 | 1.16E-03 | 2.06E-02 |
| ENSMUSG00000022098 | Bmp1 | 9607.09 | 9557.29 | 7623.23 | 7075.37 | 5790.57 | 6776.09 | -0.45 | 8.78E-04 | 1.69E-02 |
| ENSMUSG00000037824 | Tspan14 | 2020.32 | 1875.81 | 1919.59 | 1476.01 | 1468.28 | 1323.71 | -0.45 | 8.59E-04 | 1.67E-02 |
| ENSMUSG00000010122 | Slc47a1 | 7125.11 | 6861.23 | 5608.61 | 4994.32 | 4907.29 | 4489.99 | -0.45 | 8.23E-04 | 1.63E-02 |
| ENSMUSG00000027890 | Gstm4 | 6055.65 | 4607.28 | 5784.41 | 4454.94 | 4027.57 | 3611.52 | -0.44 | 1.80E-03 | 2.78E-02 |
| ENSMUSG00000031887 | Tradd | 1411.47 | 1381.16 | 1480.55 | 1178.87 | 988.92 | 976.94 | -0.44 | 7.51E-04 | 1.54E-02 |
| ENSMUSG00000000325 | Arvcf | 984.75 | 1028.16 | 935.09 | 683.64 | 726.15 | 756.89 | -0.44 | 1.49E-03 | 2.46E-02 |
| ENSMUSG00000002769 | Gnmt | 175255.00 | 151829.01 | 152661.23 | 137799.65 | 112194.85 | 102918.76 | -0.44 | 9.71E-04 | 1.83E-02 |
| ENSMUSG00000021179 | Nrde2 | 814.27 | 823.67 | 821.05 | 667.49 | 502.45 | 640.45 | -0.44 | 3.88E-03 | 4.71E-02 |
| ENSMUSG00000052712 | BC004004 | 5795.17 | 5013.98 | 4956.71 | 4394.65 | 4248.61 | 2965.07 | -0.44 | 4.04E-03 | 4.84E-02 |
| ENSMUSG00000026870 | Cutal | 2237.38 | 2106.58 | 2464.10 | 1739.77 | 1690.21 | 1585.71 | -0.44 | 2.62E-04 | 7.15E-03 |
| ENSMUSG00000035824 | Tk2 | 1486.65 | 1479.40 | 1377.92 | 1012.00 | 1092.78 | 1095.10 | -0.44 | 9.03E-04 | 1.73E-02 |
| ENSMUSG00000033880 | Lgals3bp | 5711.52 | 4950.00 | 5283.61 | 3666.88 | 4553.98 | 3541.31 | -0.44 | 1.03E-03 | 1.89E-02 |
| ENSMUSG00000026814 | Eng | 3932.63 | 4398.23 | 4036.83 | 3087.67 | 3197.55 | 2839.21 | -0.44 | 7.15E-04 | 1.49E-02 |
| ENSMUSG00000038224 | Serpinf2 | 58876.12 | 69496.53 | 62618.41 | 53035.14 | 49328.59 | 38576.77 | -0.44 | 1.26E-03 | 2.18E-02 |
| ENSMUSG00000004035 | Gstm7 | 4996.79 | 4307.98 | 4721.99 | 3784.23 | 3599.69 | 2975.35 | -0.44 | 6.42E-04 | 1.37E-02 |
| ENSMUSG00000029059 | Fam213b | 1274.87 | 1279.48 | 1139.40 | 996.93 | 845.11 | 889.61 | -0.44 | 1.01E-03 | 1.87E-02 |
| ENSMUSG00000050373 | Snx21 | 799.44 | 704.86 | 802.04 | 595.36 | 578.79 | 534.28 | -0.43 | 3.32E-03 | 4.20E-02 |
| ENSMUSG00000030382 | Slc27a5 | 68265.08 | 76246.94 | 63837.63 | 55163.57 | 50945.12 | 48168.09 | -0.43 | 2.14E-04 | 6.09E-03 |
| ENSMUSG00000047379 | B4gat1 | 1820.19 | 1838.12 | 1822.66 | 1444.79 | 1297.84 | 1317.71 | -0.43 | 3.90E-04 | 9.65E-03 |
| ENSMUSG00000026411 | Tmem9 | 1338.41 | 1289.77 | 1193.56 | 876.35 | 862.86 | 1090.82 | -0.43 | 2.28E-03 | 3.27E-02 |
| ENSMUSG00000032606 | Nicn1 | 1073.69 | 1128.69 | 1092.83 | 923.72 | 781.19 | 739.77 | -0.43 | 1.45E-03 | 2.42E-02 |
| ENSMUSG00000020534 | Shmt1 | 15715.69 | 13864.12 | 13753.55 | 11981.41 | 9036.06 | 11114.53 | -0.43 | 1.35E-03 | 2.29E-02 |
| ENSMUSG00000014791 | Elmo3 | 1409.35 | 1405.15 | 1420.68 | 1119.66 | 983.59 | 1041.16 | -0.43 | 1.66E-03 | 2.66E-02 |
| ENSMUSG00000031532 | Saraf | 9214.25 | 11341.71 | 10832.36 | 8373.74 | 7879.37 | 7046.65 | -0.43 | 5.02E-04 | 1.16E-02 |
| ENSMUSG00000002550 | Uck1 | 4074.52 | 3453.46 | 4287.71 | 2861.59 | 2543.31 | 3369.21 | -0.43 | 2.23E-03 | 3.23E-02 |
| ENSMUSG00000074207 | Adh1 | 107885.72 | 111833.73 | 105908.87 | 88982.60 | 73763.86 | 79489.29 | -0.43 | 1.31E-04 | 4.34E-03 |
| ENSMUSG00000024981 | Acsl5 | 26170.93 | 21847.18 | 22712.88 | 18130.91 | 17747.22 | 16746.71 | -0.43 | 1.91E-04 | 5.57E-03 |
| ENSMUSG00000055041 | Commd5 | 873.56 | 838.52 | 897.07 | 624.42 | 618.74 | 697.82 | -0.43 | 3.69E-03 | 4.54E-02 |
| ENSMUSG00000051716 | Apon | 26579.65 | 26501.31 | 26897.01 | 20786.87 | 20038.41 | 18703.16 | -0.43 | 2.18E-05 | 1.07E-03 |
| ENSMUSG00000040963 | Asgr2 | 10601.36 | 11730.12 | 10358.16 | 8964.79 | 7563.34 | 7835.22 | -0.42 | 3.46E-04 | 8.73E-03 |
| ENSMUSG00000001552 | Jup | 4208.99 | 4088.64 | 3511.32 | 3247.01 | 2863.77 | 2693.65 | -0.42 | 9.96E-04 | 1.86E-02 |
| ENSMUSG00000026656 | Fcgr2b | 1868.90 | 2257.37 | 2313.96 | 1562.14 | 1519.77 | 1719.28 | -0.42 | 3.40E-03 | 4.26E-02 |
| ENSMUSG00000039616 | Mocos | 4803.02 | 4915.73 | 4489.17 | 3715.32 | 4022.24 | 2863.18 | -0.42 | 3.18E-03 | 4.09E-02 |
| ENSMUSG00000005566 | Trim28 | 5088.91 | 5077.95 | 4736.25 | 4039.38 | 3410.60 | 3678.30 | -0.42 | 4.23E-04 | 1.03E-02 |
| ENSMUSG00000063524 | Eno1 | 37131.25 | 32196.15 | 35506.63 | 27759.96 | 23098.37 | 27441.69 | -0.42 | 5.84E-04 | 1.27E-02 |
| ENSMUSG00000036114 | Rpp25l | 1423.12 | 1255.49 | 1606.94 | 1141.19 | 1029.75 | 1033.45 | -0.42 | 2.28E-03 | 3.27E-02 |
| ENSMUSG00000020097 | Sgpl1 | 4705.60 | 4609.57 | 4202.18 | 3599.05 | 3731.07 | 2772.42 | -0.42 | 2.12E-03 | 3.12E-02 |
| ENSMUSG00000035637 | Grhpr | 28907.04 | 30851.55 | 29832.45 | 26012.65 | 19737.47 | 21217.87 | -0.42 | 1.47E-03 | 2.45E-02 |
| ENSMUSG00000001123 | Lgals9 | 38461.18 | 35101.26 | 35442.01 | 29734.43 | 29416.24 | 22355.78 | -0.42 | 1.36E-03 | 2.31E-02 |
| ENSMUSG00000023045 | Soat2 | 2654.58 | 2525.84 | 2375.72 | 2071.37 | 1601.44 | 1980.43 | -0.42 | 1.58E-03 | 2.56E-02 |
| ENSMUSG00000030934 | Oat | 46321.14 | 39339.55 | 45651.93 | 29990.66 | 31425.14 | 37039.86 | -0.42 | 1.44E-03 | 2.41E-02 |
| ENSMUSG00000022555 | Dgat1 | 1399.82 | 1727.30 | 1693.42 | 1296.22 | 1172.67 | 1150.75 | -0.41 | 2.65E-03 | 3.63E-02 |
| ENSMUSG00000034120 | Srsf2 | 2814.47 | 2291.65 | 2827.11 | 2205.94 | 1889.95 | 1865.69 | -0.41 | 3.06E-03 | 4.00E-02 |
| ENSMUSG00000024975 | Pdcd4 | 8496.34 | 7406.15 | 7368.55 | 6204.41 | 6159.86 | 5127.02 | -0.41 | 1.33E-03 | 2.27E-02 |
| ENSMUSG00000068876 | Cgn | 2282.91 | 2321.35 | 1842.61 | 1638.58 | 1636.95 | 1579.72 | -0.41 | 1.46E-03 | 2.43E-02 |
| ENSMUSG00000022671 | Mzt2 | 1558.65 | 1417.71 | 1405.48 | 1263.92 | 1048.39 | 994.92 | -0.41 | 4.20E-03 | 4.99E-02 |
| ENSMUSG00000020868 | Xylt2 | 1741.83 | 1905.52 | 1765.64 | 1495.39 | 1325.36 | 1263.77 | -0.41 | 1.23E-03 | 2.15E-02 |
| ENSMUSG00000071657 | Bscl2 | 4177.23 | 4002.96 | 4623.16 | 3830.52 | 3107.89 | 2732.18 | -0.41 | 4.16E-03 | 4.95E-02 |
| ENSMUSG00000001054 | Rmnd5b | 1134.05 | 1027.01 | 1072.88 | 799.91 | 813.15 | 829.67 | -0.40 | 1.39E-03 | 2.33E-02 |
| ENSMUSG00000002833 | Hdgfl2 | 2416.33 | 2111.15 | 2221.78 | 1807.60 | 1657.37 | 1639.65 | -0.40 | 6.92E-04 | 1.45E-02 |
| ENSMUSG00000026519 | Tmem63a | 1377.58 | 1376.59 | 1205.92 | 1077.67 | 980.04 | 940.98 | -0.40 | 2.22E-03 | 3.23E-02 |
| ENSMUSG00000029102 | Hgfac | 18711.22 | 15834.75 | 17248.71 | 14545.85 | 11023.66 | 13653.21 | -0.40 | 2.17E-03 | 3.17E-02 |
| ENSMUSG00000032207 | Lipc | 9746.86 | 12891.94 | 9942.88 | 8371.59 | 8208.71 | 8118.63 | -0.40 | 2.19E-03 | 3.20E-02 |
| ENSMUSG00000038150 | Ormdl3 | 9243.90 | 7568.37 | 8568.76 | 7022.62 | 6516.72 | 5755.48 | -0.40 | 2.29E-03 | 3.27E-02 |
| ENSMUSG00000004207 | Psap | 38892.14 | 39188.76 | 38803.19 | 30496.66 | 31734.07 | 26617.16 | -0.40 | 8.33E-04 | 1.64E-02 |
| ENSMUSG00000028737 | Aldh4a1 | 22613.14 | 20038.77 | 21325.46 | 18036.17 | 14158.18 | 16536.08 | -0.39 | 1.30E-03 | 2.23E-02 |
| ENSMUSG00000028857 | Tmem222 | 1498.30 | 1423.43 | 1581.28 | 1200.40 | 1058.16 | 1174.73 | -0.39 | 1.88E-03 | 2.86E-02 |
| ENSMUSG00000073418 | C4b | 84995.16 | 113035.53 | 95870.95 | 70332.77 | 71643.11 | 82023.69 | -0.39 | 2.83E-03 | 3.77E-02 |
| ENSMUSG00000067235 | H2-Q10 | 247223.99 | 209207.00 | 227275.15 | 202823.74 | 168966.95 | 149375.28 | -0.39 | 3.63E-03 | 4.48E-02 |
| ENSMUSG00000023010 | Tmbim6 | 141463.42 | 134642.81 | 142041.74 | 117180.73 | 104723.84 | 97430.42 | -0.39 | 4.33E-04 | 1.04E-02 |
| ENSMUSG00000092545 | Gm20319 | 1482.41 | 1317.18 | 1497.66 | 1139.04 | 1084.79 | 1063.42 | -0.39 | 1.83E-03 | 2.80E-02 |
| ENSMUSG00000001155 | Ftcd | 33948.30 | 30848.12 | 34462.27 | 29585.86 | 21700.21 | 24615.33 | -0.39 | 3.30E-03 | 4.19E-02 |
| ENSMUSG00000023829 | Slc22a1 | 27373.80 | 25205.83 | 29535.01 | 21957.12 | 20441.43 | 20507.21 | -0.38 | 6.52E-04 | 1.38E-02 |
| ENSMUSG00000029716 | Tfr2 | 39855.71 | 45473.08 | 36644.13 | 34315.34 | 28690.09 | 30602.84 | -0.38 | 2.19E-03 | 3.20E-02 |
| ENSMUSG00000016554 | Eif3d | 2596.34 | 2750.89 | 2628.50 | 2213.48 | 1996.47 | 1930.76 | -0.38 | 9.82E-04 | 1.84E-02 |
| ENSMUSG00000024101 | Washc1 | 1378.64 | 1279.48 | 1346.56 | 1029.22 | 1069.70 | 984.65 | -0.38 | 2.78E-03 | 3.73E-02 |
| ENSMUSG00000025791 | Pgm2 | 14699.18 | 14038.91 | 14017.73 | 11193.34 | 9952.18 | 11814.06 | -0.38 | 9.40E-04 | 1.78E-02 |
| ENSMUSG00000031980 | Agt | 34701.15 | 44235.86 | 35120.82 | 31652.92 | 29620.41 | 26722.47 | -0.37 | 3.79E-03 | 4.63E-02 |
| ENSMUSG00000024292 | Cyp4f14 | 21210.14 | 20157.58 | 17533.80 | 15684.89 | 14001.94 | 15766.34 | -0.37 | 1.94E-03 | 2.92E-02 |
| ENSMUSG00000021215 | Net1 | 3809.80 | 3564.28 | 3479.01 | 2482.62 | 3101.68 | 2789.55 | -0.37 | 2.94E-03 | 3.89E-02 |
| ENSMUSG00000028809 | Srrm1 | 3052.71 | 3028.49 | 2759.64 | 2116.58 | 2232.61 | 2482.17 | -0.37 | 1.78E-03 | 2.76E-02 |
| ENSMUSG00000020277 | Pfkl | 4790.31 | 4840.33 | 4591.80 | 3816.52 | 3581.93 | 3673.16 | -0.36 | 9.26E-04 | 1.76E-02 |
| ENSMUSG00000035237 | Lcat | 25531.37 | 27797.92 | 24538.39 | 22281.18 | 19265.21 | 19113.29 | -0.36 | 1.71E-03 | 2.69E-02 |
| ENSMUSG00000024747 | Aldh1a7 | 18864.75 | 19463.00 | 19448.63 | 15148.75 | 16141.34 | 13747.39 | -0.36 | 8.73E-04 | 1.69E-02 |
| ENSMUSG00000029445 | Hpd | 127546.74 | 137448.53 | 118697.87 | 105950.79 | 91675.30 | 102509.49 | -0.35 | 2.01E-03 | 3.00E-02 |
| ENSMUSG00000013622 | Atraid | 3297.31 | 3386.06 | 3269.00 | 2751.77 | 2430.57 | 2618.31 | -0.35 | 3.41E-03 | 4.26E-02 |
| ENSMUSG00000025793 | Hgs | 4222.76 | 4429.07 | 3888.59 | 3540.92 | 3143.40 | 3144.88 | -0.35 | 2.84E-03 | 3.78E-02 |
| ENSMUSG00000021939 | Ctsb | 41072.35 | 43858.87 | 38492.44 | 31996.36 | 33266.26 | 31477.89 | -0.35 | 7.87E-04 | 1.59E-02 |
| ENSMUSG00000056167 | Cnot10 | 1213.46 | 1246.35 | 1175.51 | 944.17 | 931.21 | 974.37 | -0.35 | 3.92E-03 | 4.73E-02 |
| ENSMUSG00000000753 | Serpinf1 | 38150.94 | 38731.80 | 35653.93 | 32139.54 | 28357.19 | 27813.29 | -0.35 | 1.72E-03 | 2.70E-02 |
| ENSMUSG00000027709 | Mccc1 | 3142.71 | 3308.38 | 3220.53 | 2535.38 | 2756.36 | 2298.08 | -0.35 | 4.02E-03 | 4.83E-02 |
| ENSMUSG00000025176 | Hoga1 | 10638.42 | 11299.44 | 10106.33 | 8621.36 | 7747.10 | 8860.97 | -0.34 | 2.38E-03 | 3.35E-02 |
| ENSMUSG00000020476 | Dbnl | 2406.80 | 2247.09 | 2331.06 | 1915.26 | 1812.72 | 1783.50 | -0.34 | 3.03E-03 | 3.97E-02 |
| ENSMUSG00000061740 | Cyp2d22 | 16521.48 | 16030.10 | 15431.76 | 13840.68 | 12483.06 | 11643.67 | -0.34 | 2.70E-03 | 3.67E-02 |
| ENSMUSG00000007564 | Ppp2r1a | 5458.45 | 6034.14 | 5524.99 | 4874.81 | 4474.09 | 4135.52 | -0.34 | 3.77E-03 | 4.61E-02 |
| ENSMUSG00000025934 | Gsta3 | 103179.06 | 93341.76 | 89926.89 | 78486.89 | 74599.20 | 73951.29 | -0.34 | 1.89E-03 | 2.87E-02 |
| ENSMUSG00000020884 | Asgr1 | 32196.93 | 31590.68 | 35031.49 | 28304.72 | 23887.55 | 26296.93 | -0.33 | 2.99E-03 | 3.94E-02 |
| ENSMUSG00000016253 | Nelfcd | 2596.34 | 2423.02 | 2526.82 | 2101.51 | 1986.71 | 1947.89 | -0.32 | 3.90E-03 | 4.72E-02 |
| ENSMUSG00000034285 | Nipsnap1 | 21397.56 | 19757.74 | 20677.36 | 17419.28 | 15906.98 | 16354.56 | -0.32 | 2.24E-03 | 3.24E-02 |
| ENSMUSG00000064120 | Mocs1 | 10876.67 | 10698.54 | 9899.17 | 8701.03 | 8179.41 | 8441.42 | -0.31 | 2.95E-03 | 3.89E-02 |
