# Supplementary material for: Role of GPRC6A in Regulating Hepatic Energy Metabolism in Mice
Source: Sci Rep. 2020 Apr 29;10:7216. doi: 10.1038/s41598-020-64384-8 (PMC7190669; doi:10.1038/s41598-020-64384-8)
Supplement: Supplementary file 2 — Supplementary Information. [file 41598_2020_64384_MOESM2_ESM.pptx]

## Slide 1
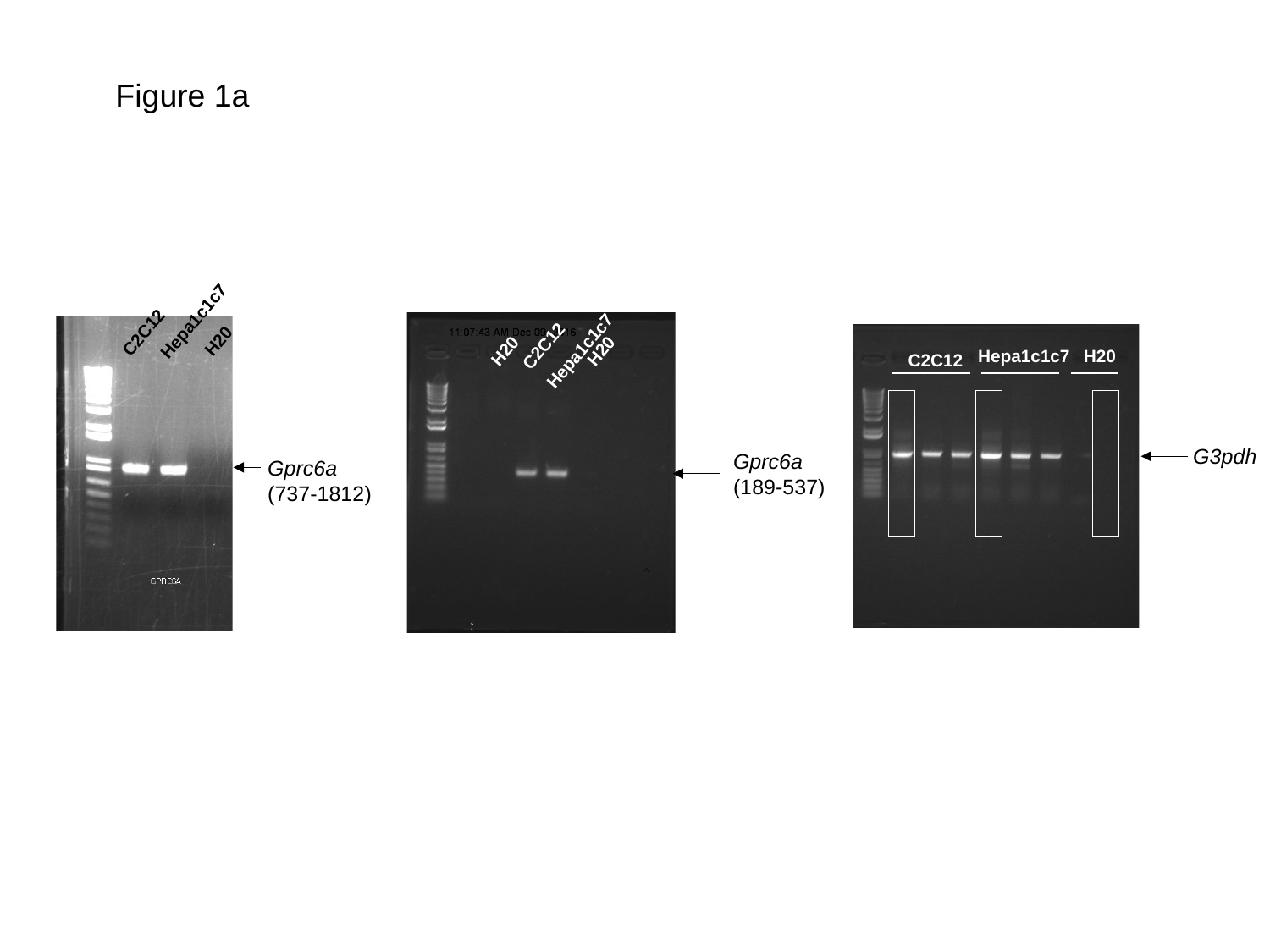

Figure 1a
C2C12
Hepa1c1c7
Hepa1c1c7
C2C12
H20
C2C12
H20
Hepa1c1c7
H20
Hepa1c1c7
H20
C2C12
G3pdh
Gprc6a
(189-537)
Gprc6a
(737-1812)

## Slide 2
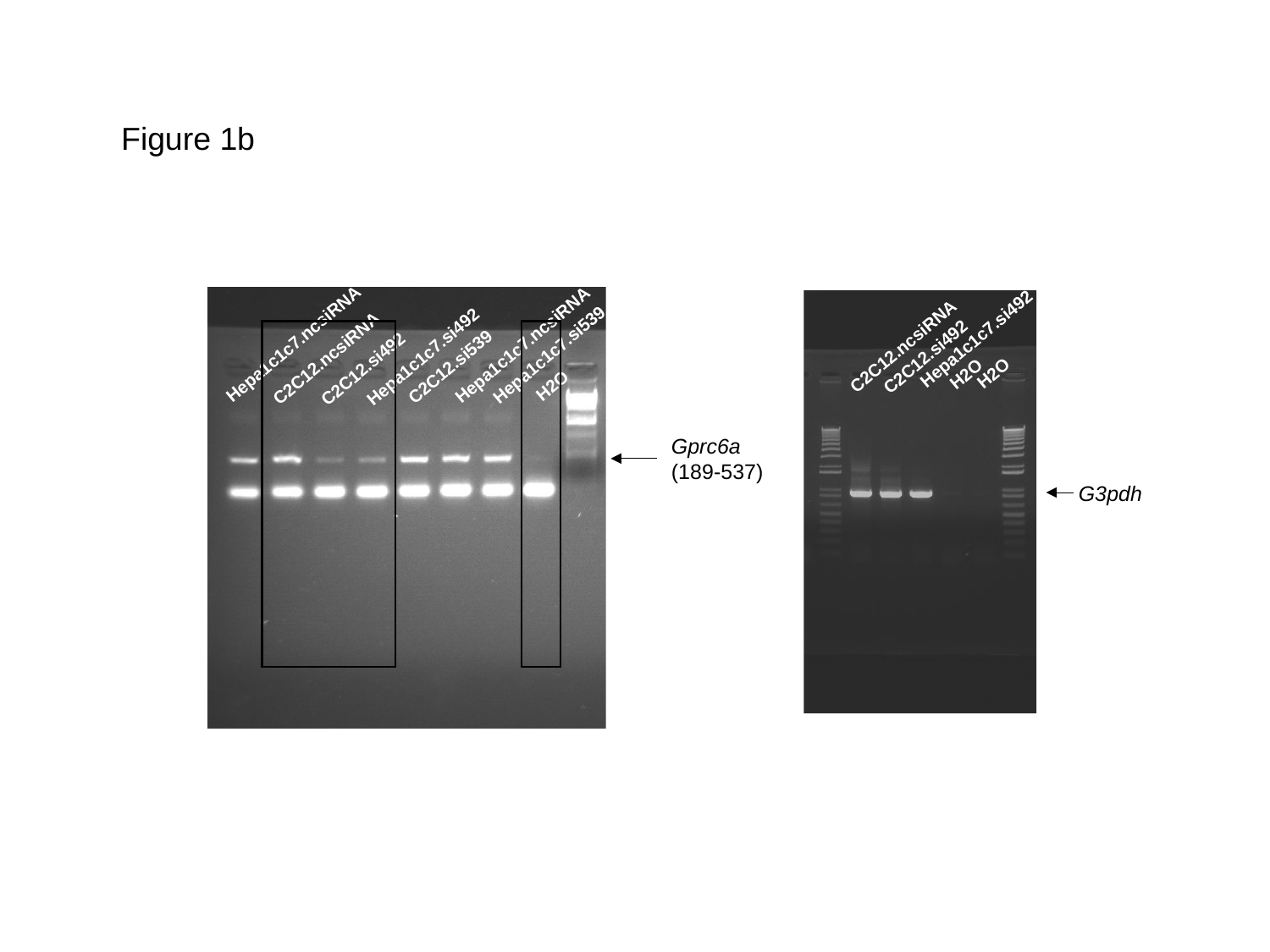

Figure 1b
Hepa1c1c7.si492
Hepa1c1c7.ncsiRNA
Hepa1c1c7.ncsiRNA
C2C12.ncsiRNA
Hepa1c1c7.si539
C2C12.si492
Hepa1c1c7.si492
C2C12.ncsiRNA
C2C12.si539
C2C12.si492
H2O
H2O
H2O
Gprc6a
(189-537)
G3pdh

## Slide 3
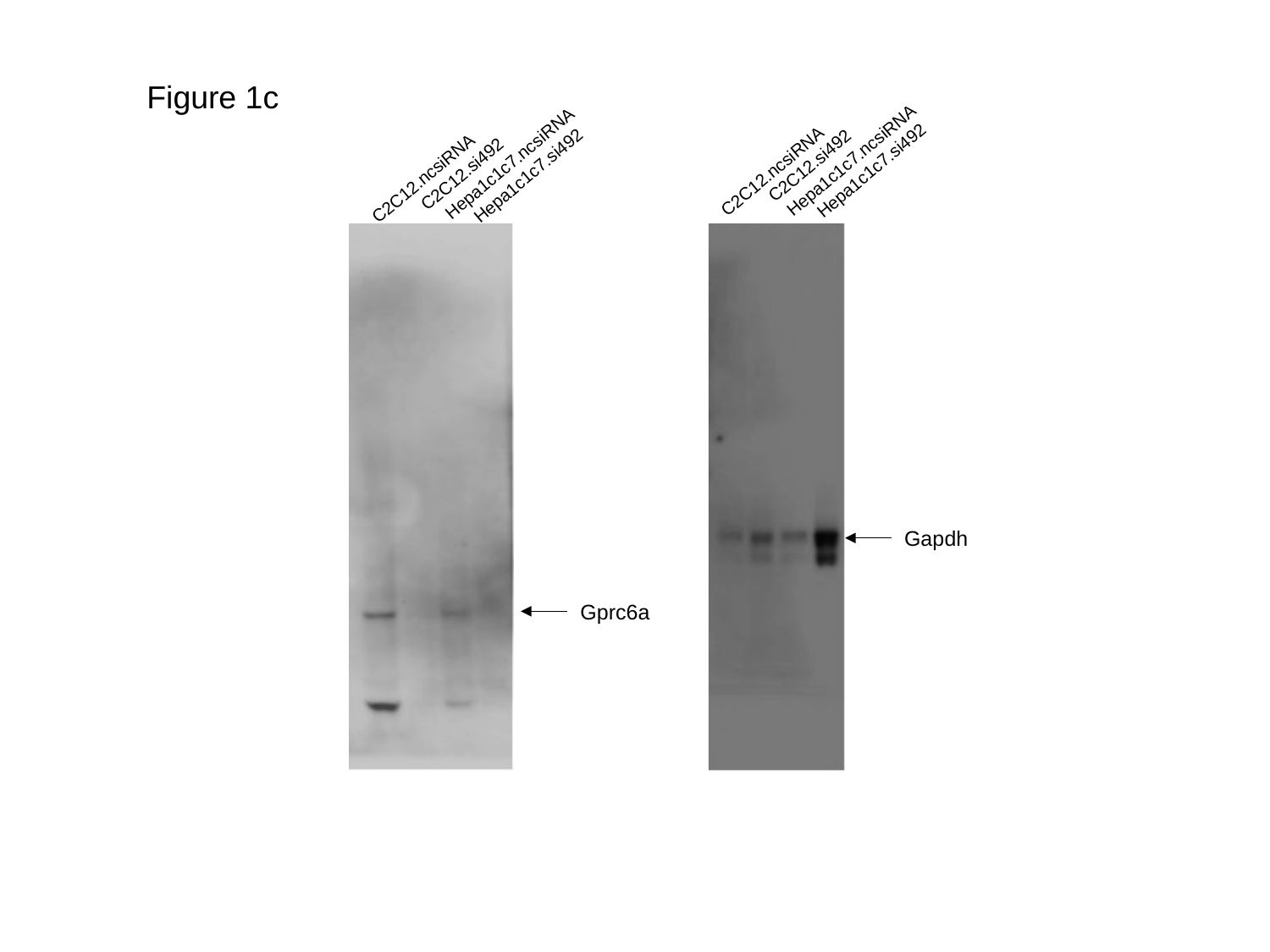

Figure 1c
Hepa1c1c7.ncsiRNA
Hepa1c1c7.ncsiRNA
C2C12.si492
Hepa1c1c7.si492
C2C12.ncsiRNA
C2C12.si492
Hepa1c1c7.si492
C2C12.ncsiRNA
Gapdh
Gprc6a

## Slide 4
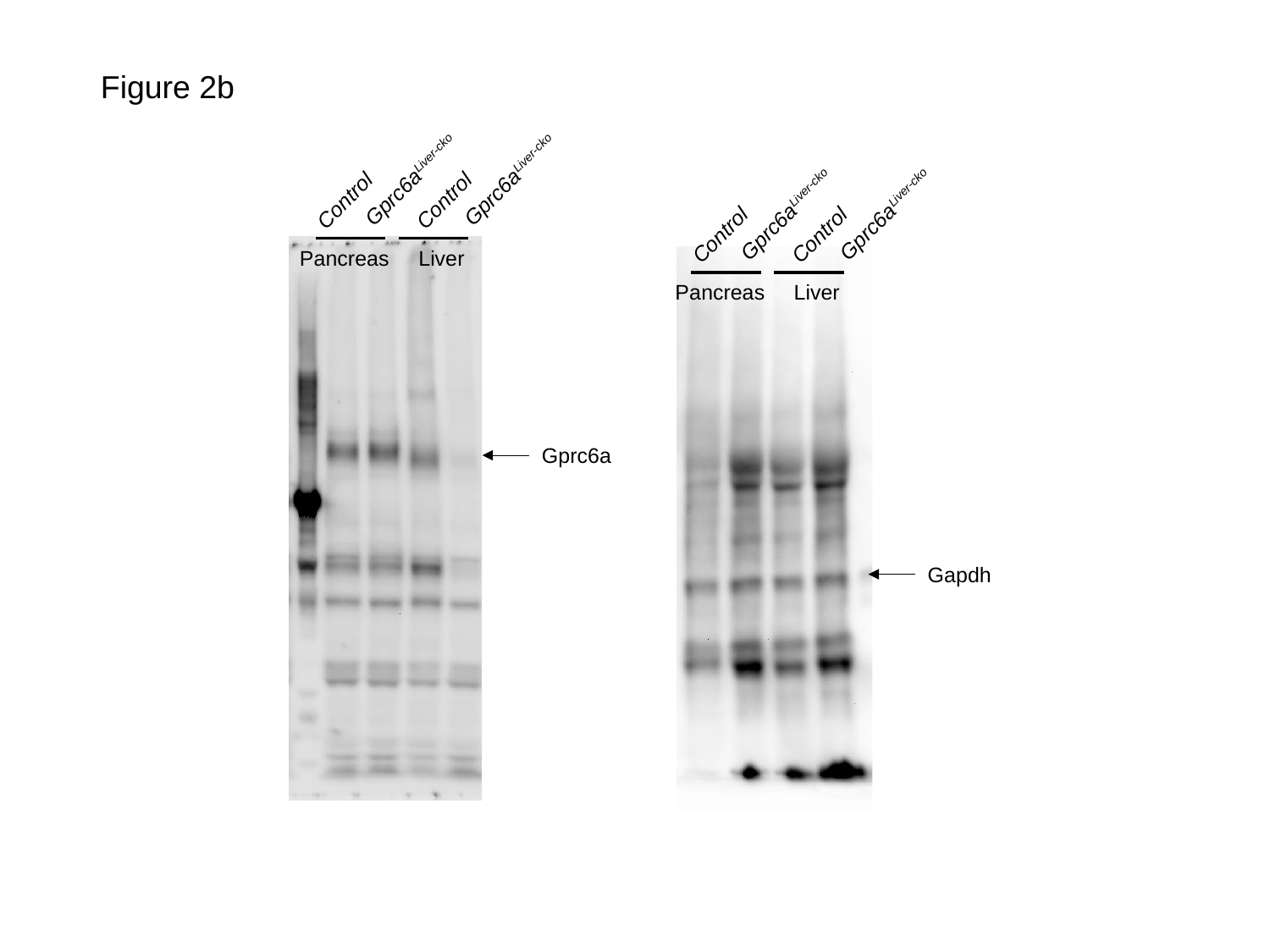

Figure 2b
Gprc6aLiver-cko
Gprc6aLiver-cko
Control
Control
Gprc6aLiver-cko
Gprc6aLiver-cko
Control
Control
Pancreas
Liver
Pancreas
Liver
Gprc6a
Gapdh
